# Rapid discovery and identification of anti-inflammatory constituents from traditional Chinese medicine formula by activity index, LC-MS, and NMR

**DOI:** 10.1038/srep31000

**Published:** 2016-08-08

**Authors:** Shufang Wang, Haiqiang Wang, Yining Liu, Yi Wang, Xiaohui Fan, Yiyu Cheng

**Affiliations:** 1 Pharmaceutical Informatics Institute, College of Pharmaceutical Sciences, Zhejiang University, Hangzhou, P.R. China

## Abstract

The traditional activity-guided approach has the shortcoming of low accuracy and efficiency in discovering active compounds from TCM. In this work, an approach was developed by integrating activity index (AI), liquid chromatography – mass spectrometry (LC-MS), and nuclear magnetic resonance (NMR) to rapidly predict and identify the potential active constituents from TCM. This approach was used to discover and identify the anti-inflammatory constituents from a TCM formula, Gui-Zhi-Jia-Shao-Yao-Tang (GZJSYT). The AI results indicated that, among the 903 constituents detected in GZJSYT by LC-MS, 61 constituents with higher AI values were very likely to have anti-inflammatory activities. And eight potential active constituents of them were isolated and validated to have significant inhibitory effects against NO production on LPS-induced RAW 264.7 cell model. Among them, glycyrrhisoflavone (836), glisoflavanone (893) and isoangustone A (902) were reported to have anti-inflammatory effects for the first time. The proposed approach could be generally applicable for rapid and high efficient discovery of anti-inflammatory constituents from other TCM formulae or natural products.

Traditional Chinese medicine (TCM) is a valuable resource for new drug and lead compound discovery. The most common way to find active compounds from TCM is activity-guided approach. The whole extract of TCM was systematically separated to several fractions, which then were carried out on activity screening. The compounds in active fractions were isolated and evaluated pharmacologically to find the active compounds. Generally one fraction composes of more than ten constituents. Since the activities of constituents are unknown before biological testing, tremendous amount of time and efforts were spent on isolation of inactive compounds, which significantly hampers rapid discovery of compounds of interest, *e.g.* those with appreciable pharmacological effects. To overcome this drawback, in our previous study^1^, activity index (AI) was proposed to predict the contribution of each constituent detected in TCM formula to the anti-inflammatory effect. The constituents with positive AI values might be active, while the ones with negative or near zero AI values might be inactive or had weak activity. This method had been successfully used to find several anti-inflammatory constituents in Ju-Zhi-Jiang-Tang. However, only the constituents detected in the whole extract of Ju-Zhi-Jiang-Tang were evaluated. The trace constituents that were detected only in the fractions were not involved, which might omit some constituents that had significant activity though their contents in the formula were low.

Liquid chromatography – mass spectrometry (LC-MS) is a rapid, sensitive, and high efficient technique, and has been increasingly used for the identification of constituents in TCM formulae. But the constituents in TCM are complex, and their concentrations in TCM differ by several orders of magnitude. Generally 50 to more than 100 constituents are detected in the whole extract of TCM formula by LC-MS^2^,^3^. Preliminary fractionation could enrich the concentrations of trace constituents, which would be helpful for detecting trace constituents in TCM formula by LC-MS analysis^4^,^5^. Thus the constituents in the whole extract and fractions could be evaluated on their AIs. Semi-preparative LC guided by LC-MS was used to prepare the potential active constituents for activity validation, and the structures of potential active constituents could be identified by nuclear magnetic resonance (NMR)^3^.

Gui-Zhi-Jia-Shao-Yao-Tang (GZJSYT), a well-known TCM formula from Shang-Han-Lun, is comprised of *Cinnamomi ramulus* (Guizhi in Chinese), *Paeoniae radix alba* (Baishao in chinese), *Zingiberis rhizoma recens* (Shengjiang in Chinese), *Jujubae fructus* (Dazao in Chinese), and *Glycyrrhizae radix et rhizoma preparata cum melle* (Zhigancao in Chinese). It is often used to treat gastralgia, dysentery, peptic ulcer, tuberculous peritonitis, prosopalgia, and restless legs syndrome in clinic. Pharmacology research indicated that the mechanism of antidiarrheal effect of GZJSYT might be the inhibition of excessively accelerated small intestinal movement and the acetylcholine released by parasympathetic nerves^6^. In addition, it was reported that GZJSYT exerted therapeutic effect against peptic ulcer by alleviating gastro spasm^7^. However, there has been no research on the comprehensive identification of chemical profile of GZJSYT, and the active ingredients are still unclear, which would hamper its broad application^8^.

In this work, the approach based on AI, LC-MS, and NMR was used to discover and identify the anti-inflammatory constituents from GZJSYT. The whole extract and fractions of GZJSYT were analyzed by LC-Q-TOF-MS and LC-IT-MS, and a total number of 903 constituents were detected. In addition, the anti-inflammatory effects of the whole extract and fractions were evaluated on LPS-induced NO production in RAW 264.7 macrophages. The AI method was used to evaluate the contributions of 903 constituents to the anti-inflammatory effects of the whole extract and fractions. Finally, eight potential active constituents were prepared, and their anti-inflammatory activities were validated.

## Results

### Characterization of chemical constituents in the extract of GZJSYT by LC-MS

**Figure 1:**
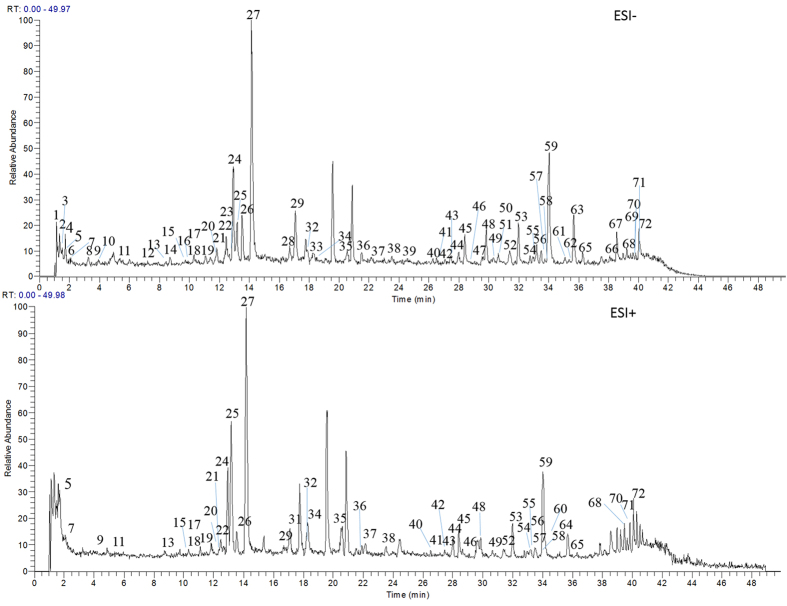
The total ion current (TIC) chromatograms of GZJSYT in negative and positive modes by LC-IT-MS.

**Figure 2:**
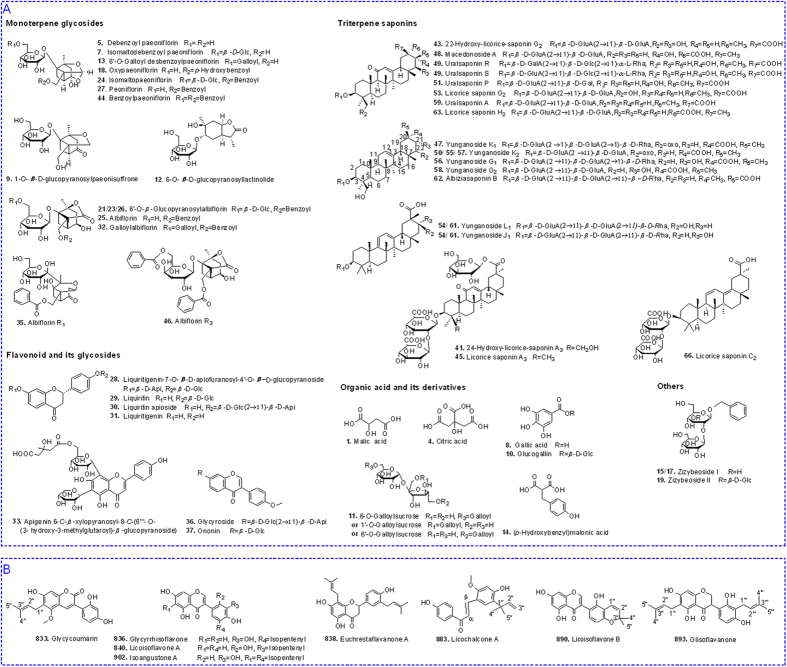
The chemical structures of identified constituents in the whole extract of GZJSYT (**A**) and eight potential active constituents isolated by semi-preparative LC (**B**).

In order to separate as many as possible constituents in one chromatographic separation, chromatographic separation conditions, including the elution gradient and flow rate were optimized to obtain good peak capacity. Finally, the optimum chromatographic conditions were used for LC-MS analysis. The extract of GZJSYT was analyzed by LC-Q-TOF-MS and LC-IT-MS method in negative and positive ion modes to acquire the accurate mass and MS^n^ information that was indispensable for profiling the constituents. The total ions current (TIC) chromatograms obtained by LC-IT-MS are shown in Fig. 1. It was found that most constituents were detected in negative ion mode, except several compounds could only be detected in positive ion mode (Table 1). A total number of 72 constituents, including 34 ones from *Glycyrrhizae radix et rhizoma preparata cummelle*, 29 ones from *Paeoniae radix alba*, ten ones from *Jujubae fructus*, six ones from *Cinnamomiramulus*, and three ones from *Zingiberis rhizoma recens*, were detected in the whole extract of GZJSYT. Among them, 58 constituents were identified by comparing with the retention times and MS^n^ data of the reference standards or by referring to the literatures. As shown in Fig. 2A, the identified constituents mainly included monoterpene glycosides, triterpene saponins, flavonoid and its glycosides, gallic acid derivatives, and organic acids.

A total number of 16 monoterpene glycosides in the root of *Paeoniae radix alba* were observed in negative ion mode. Monoterpene glycosides in the formula mostly possess a “cage-like” pinane skeleton with the hemiketal group or lactone group^9^,^10^. In the present work, the monoterpene with similar pinane skeleton of paeoniflorin was defined as paeoniflorin type (PT), and that with similar skeleton of albiflorin was defined as albiflorin type (AT).

**Figure 3:**
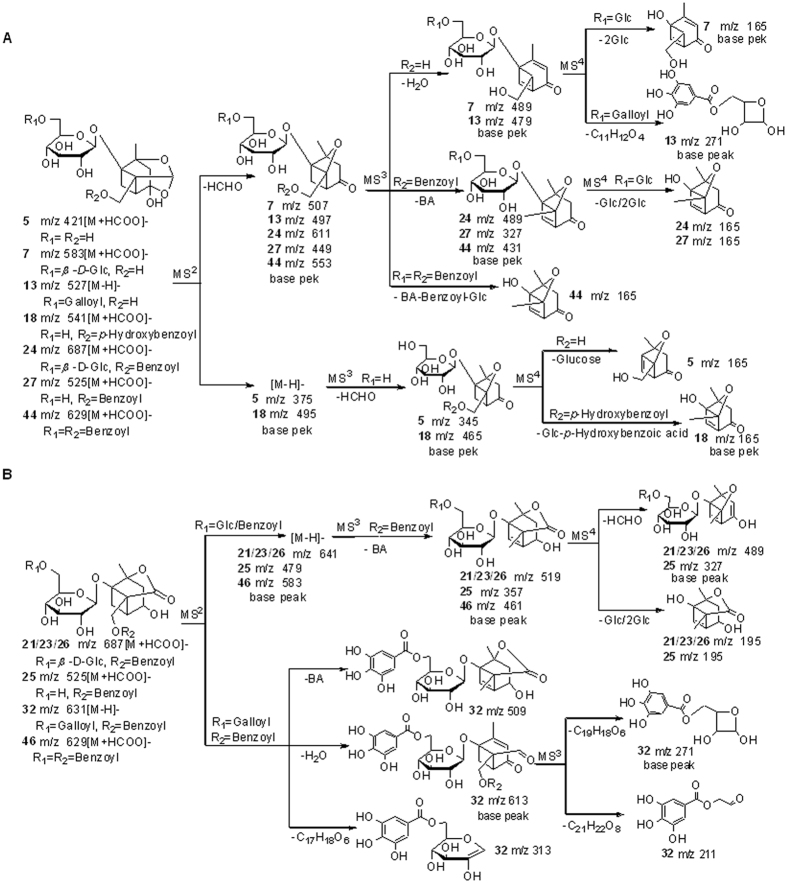
The proposed fragmentation pathway of PTs (**A**) and ATs (**B**) in negative ion mode.

Compounds 7, 13, 24, 27, and 44 were identified as PTs. Their fragmentation pathways were summarized in Fig. 3A. The characteristic fragment ions of PTs were the base peak ion [M − HCHO − H]^−^ in MS^2^ spectra, in which the neutral loss of HCHO (30 Da) was speculatively related to pinane skeleton of PTs due to the instability of hemiketal group. In MS^3^ spectra, compounds 7 and 13 generated base peak ions at *m/z* 489 [M − HCHO − H_2_O − H]^−^ and *m/z* 479 [M − HCHO − H_2_O − H]^−^, respectively. But compounds 24, 27, and 44 produced base peak ions [M − HCHO − BA − H]^−^ in MS^3^ spectra. The characteristic fragment ion at *m/z* 165 was observed in MS^3^ or MS^4^ spectra of compounds 7, 24, 27, and 44. For compound 44, *m/z* 165 [M–HCHO–BA–Benzoyl–Glc–H]^−^ was observed as a fragment ion in MS^3^ spectrum, while for compounds 7, 24, and 27, *m/z* 165 was generated as base peak ions in MS^4^ spectra by the loss of one glucosyl residue (162 Da) (for compound 27) or two glucosyl residues (324 Da) (for compounds 7 and 24) from the precursor ions. For compound 13, the base peak ion in MS^4^ spectrum was at *m/z* 271, which was produced by the loss of pinane skeleton and a glucosyl segment (42 Da) from the precursor ions. Finally, compound 27 was identified as paeoniflorin by comparing with the reference standard. Compounds 7, 13, 24, and 44 were supposed to be isomaltodebenzoyl paeoniflorin, 6′-*O*-galloyl desbenzoyl paeoniflorin, isomaltopaeoniflorin, and benzoylpaeoniflorin, respectively, by referring to the literatures^11^,^12^.

Compounds 5 and 18 both showed adduct ions [M + HCOO]^−^ in MS spectra, as well as base peak ions [M − H]^−^ in MS^2^ spectra. The characteristic fragment ions, including base peak ions [M − HCHO − H]^−^ in MS^3^ spectra and the fragment ion at *m/z* 165 in MS^4^ spectra, manifested the typical fragmentation pattern of PTs, *i.e*, successive loss of unstable hemiketal group and the substituent group from pinane skeleton. Besides, the fragment ion at *m/z* 327 [M − HCHO − 138 − H]^−^ in MS^3^ spectrum of compound 18 was detected. Here, the loss of 138 Da was deduced to be *p*-hydroxybenzoic acid. Base on the MS^n^ fragmentation behaviour mentioned above, compounds 5 and 18 were ascribed to be desbenzoyl paeoniflorin and oxypaeoniflorin, respectively.

Compounds 21, 23, 25, and 26 were identified as ATs with the lactone pinane skeleton. Their fragmentation pathways are shown in Fig. 3B. They all showed adduct ions [M + HCOO]^−^ in MS spectra, and produced deprotonated molecules [M − H]^−^ as base peak ions in MS^2^ spectra. Compounds 21, 23, and 26 shared the identical molecular formula C_29_H_38_O_16_, and gave the similar fragmentation behavior as compound 25. The four compounds all generated the typical base peak ions [M − BA − H]^−^ in MS^3^ spectra and [M − BA − HCHO − H]^−^ in MS^4^ spectra. According to the MS^n^ data, compound 25 was assigned as albiflorin. Nevertheless, compounds 21, 23, and 26 could not be confirmed only by MS^n^ data, they were tentatively deduced to be 6′-*O*-*β*-Glucopyranosyl albiflorin or its isomers.

Compounds 32 and 46 were also identified as ATs. Compound 32, containing a galloyl at C-6′, produced the ion at *m/z* 613 [M − H_2_O − H]^−^ as base peak ion in MS^2^ spectrum, instead of *m/z* 509 [M − BA − H]^−^. In MS^3^ spectrum, the base peak ion was at *m/z* 271 [Gallyol + 120 − H]^−^ by the loss of monoterpenoid skeleton. The reason might be galloyl group was easier to ionize than monoterpenoid skeleton. For compound 46, base peak ion was at *m/z* 583 [M − H]^−^ in MS^2^ spectrum, as well as *m/z* 461 [M − BA − H]^−^ in MS^3^ spectrum. By comparing with the data in literatures^13^,^14^, compounds 32 and 46 were tentatively deduced as galloylalbiflorin and albiflorin R_3_, respectively.

Compounds 9 and 35 possessed another “cage-like” pinane skeleton that was different from PTs and ATs. Compound 9 exhibited adduct ion at *m/z* 405 [M + HCOO]^−^ in MS spectrum and base peak ion at *m/z* 359 [M − H]^−^ in MS^2^ spectrum. Furthermore, its MS^3^ spectrum showed base peak ion at *m/z* 197 [M − Glc − H]^−^, as well as the “cage-like” pinane skeleton ion at *m/z* 179 [M − Glucose − H]^−^ and fragment ion at *m/z* 161 [M − Glucose − H_2_O − H]^−^. Therefore, compound 9 was ascribed to be 1-*O*-*β*-D-Glucopyranosyl paeonisuffrone^15^. For compound 35, it showed adduct ion at *m/z* 525 [M + HCOO]^−^ in MS spectrum and base peak ion at *m/z* 479 [M − H]^−^ in MS^2^ spectrum. In MS^3^ spectrum, base peak ion at *m/z* 449 [M − HCHO − H]^−^ was yielded, together with the fragment ions at *m/z* 461 [M − H_2_O − H]^−^ and *m/z* 357 [M − BA − H]^−^, indicating the existence of “cage-like” pinane skeleton. Additionally, base peak ion at *m/z* 327 in MS^4^ spectrum corresponded to the loss of benzoic acid from the precursor ion at *m/z* 449 [M − HCHO − H]^−^. The predominant fragmentation pathway was similar to that in the literature^15^, so compound 35 was identified as albiflorin R_1_.

Compound 12 produced adduct ion at *m/z* 407 [M + HCOO]^−^ in MS spectrum and base peak ion at *m/z* 361 [M − H]^−^ in MS^2^ spectrum. The fragment ions at *m/z* 199 [M − Glc − H]^−^, *m/z* 163 [M − Glc − 2H_2_O − H]^−^, and *m/z* 155 [M − Glc − CO_2_ − H]^−^ were observed in MS^3^ spectrum. The above MS^n^ data was accordance with that in the literature^15^, so compound 12 was presumed as 6-*O*-*β*-D-glucopyranosyl lactinolide.

Triterpene saponins were the main constituents of *Glycyrrhizae radix et rhizoma*, and performed a variety of pharmacological activities^16^,^17^,^18^. In this work, 22 oleanane triterpene saponins were identified in the extract of GZJSYT. These oleanane-type triterpene saponins were analyzed in negative ion mode. Their fragment patterns were similar to that reported in our previous works^19^,^20^. They were grouped into four types according to base peak ions in MS^2^ spectra: (a) base peak ion at *m/z* 351, deriving from a GluA-GluA unit of C-3 of A ring for compounds 40, 43, 48, 50, 53, 55, 57, 58, 59, 63, 65, and 66; (b) base peak ion at *m/z* 497 [2GluA + Rha − H]^−^, demonstrating the presence of Rha-GluA-GluA group at C-3 of A ring for compounds 47, 54, 56, 61, and 62; (c) base peak ion at *m/z* 485 [Aglycone − H]^−^ for compounds 49 and 51. In MS^3^ spectra, they produced base peak ion at *m/z* 355 via the cleavage of C18-C19 bond and C17-C22 bond at E-ring; (d) base peak ion [M − Glc − H]^−^, indicating the existence of an ester glycoside at C-30 of E ring for compounds 41, 42, and 45. And they generated base peak ions at *m/z* 351 [2GluA − H]^−^ in MS^3^ spectra.

Seven flavonoids were identified in the extract of GZJSYT, which were all from *Glycyrrhizae radix et rhizoma*. By comparison with the reference standards, compounds 28, 29, 30, 31, 36, and 37 were explicitly characterized as liquiritigenin-7-*O*-*β*-D-apiofuranosyl -4′-*O*-*β*-D-glucopyranoside, liquiritin, liquiritin apioside, liquiritigenin, glycyroside, and ononin, respectively. Compound 33 showed *m/z* 707 [M − H]^−^ in MS spectrum, and gave the characteristic fragment ions at *m/z* 605 [M − 102 − H]^−^, *m/z* 563 [M − 144 − H]^−^, *m/z* 473 [M − 144 − 90 − H]^−^, and *m/z* 443 [M − 144 − 120 − H]^−^ (base peak) in MS^2^ spectrum. Here, the loss of 102 Da and 144 Da indicated the presence of 3-hydroxy-3-methylglutaryl, and 90 Da and 120 Da was from glucosyl segment. In MS^3^ spectrum, the ions at *m/z* 425 [M − 144 − 120 − H_2_O − H]^−^, 383 [M − 144 − 120 − 60 − H]^−^, 353 [M − 144 − 120 − 90 − H]^−^, and 323 [M − 144 − 120 − 120 − H]^−^ were detected. So, compound 33 was characterized as apigenin 6-*C*-*β*-xylopyranosyl-8-*C*-(6′″-*O*-(3-hydroxy-3-methylglutaroyl)-*β*-glucopyranoside) by comparing the above MS^n^ data with that in the literature^19^.

Four organic acids (compounds 1, 4, 8, and 14) and two organic acid glycosides (compounds 10 and 11) were identified. The characteristic neutral loss of H_2_O, HCOOH (or CH_3_COOH), and CO_2_ occurred in MS^2^ or MS^3^ spectra of four organic acids. Among them, compounds 4 and 8 were confirmed as citric acid and gallic acid, respectively, by comparing with the reference standards. Compounds 1 (*m/z* 133) and 14 (*m/z* 209) were inferred as malic acid and (*p*-hydroxybenzyl)malonic acid, respectively. Compound 10 exhibited deprotonated molecule [M − H]^−^ at *m/z* 331. It produced base peak ion at *m/z* 271 [M − 60 − H]^−^ in MS^2^ spectrum, as well as the fragment ions at *m/z* 241 [M − 90 − H]^−^ and *m/z* 169 [M − 162 − H]^−^. In MS^3^ spectrum, it generated base peak ion at *m/z* 211 [M − H − 60 − 60]^−^ and fragment ion at *m/z* 169 [M − H − 60 − 102]^−^. Finally, compound 10 was deduced as glucogallin according to the above MS^n^ data. Compound 11 gave deprotonated molecule [M − H]^−^ at *m/z* 493. The base peak ion at *m/z* 313 [M − 180 − H]^−^ in MS^2^ spectrum indicated the existence of glucose or fructose in the molecular structure. In MS^3^ spectrum, the base peak ion at *m/z* 169 [M − 180 − 144 − H]^−^ (gallic acid) and fragment ion at *m/z* 223 [M − 180 − 90 − H]^−^ were observed. Based on the MS^n^ data mentioned above, compound 11 was deduced to be galloylsucrose. However, the linkage position of saccharide groups was not clear only by MS^n^ data.

Besides the above mentioned types of compounds, three other types of compounds (15, 17, and 19) from *Jujubae fructus* were identified. Both compounds 15 and 17 showed molecular formulae of C_19_H_28_O_11_ and gave adduct ions at *m/z* 477 [M + HCOO]^−^ in MS spectra. Though they had identical base peak ions, *i.e.*, deprotonated molecules at *m/z* 431 [M − H]^−^ in MS^2^ spectra and base peak ions at *m/z* 269 [M − Glc − H]^−^ in MS^3^ spectra, they had some different fragment ions in MS^2^ and MS^3^ spectra. By comparing the MS^n^ data with those in the literature^21^, compounds 15 and 17 were deduced to be zizybeoside I or its isomers. Compound 19 gave adduct ion at *m/z* 639 [M + HCOO]^−^ in MS spectrum. It produced base peak ion at *m/z* 593 [M − H]^−^ in MS^2^ spectrum, and base peak at *m/z* 431 [M − Glc − H]^−^ and fragment ion at *m/z* 269 [M − 2Glc − H]^−^ in MS^3^ spectrum. Finally, compound 19 was identified as zizybeoside II^21^.

### Prediction of the potential anti-inflammatory constituents by AI method

**Figure 4:**
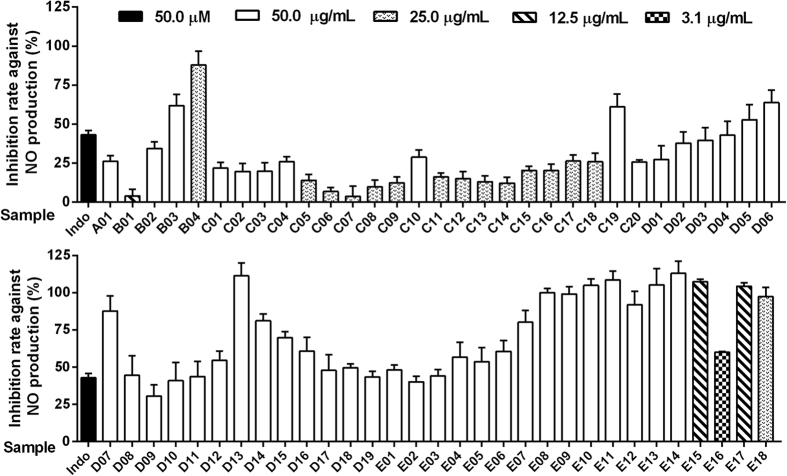
The inhibition rate against NO production of the whole extract of GZJSYT and its fractions at the corresponding concentrations without cytotoxicity.

The whole extract of GZJSYT was separated to 64 fractions by macroporus resin column chromatography and preparative LC. The fractions (except fractions D20, E19, and E20 due to little amount) were analyzed by LC-IT-MS to detect the chemical constituents in these fractions, and to acquire the peak area values of the constituents. As a result, a total number of 903 constituents were detected in the fractions. The anti-inflammatory activities of the whole extract and fractions of GZJSYT were evaluated on LPS-induced RAW 264.7 macrophages at corresponding concentrations without cytotoxicity. As shown in Fig. 4, the whole extract of GZJSYT (A01) exhibited lower inhibition rate against NO production at about 26%. But fractions B03, B04, C19, D04–D08, D11–D19, E01, and E03–E18 performed better inhibition rate than indomethacin (positive control). In particular, fractions B04, D07, D13, D14, E07–E15, E17, and E18 exerted inhibition rate against NO production at more than 80%.

**Figure 5:**
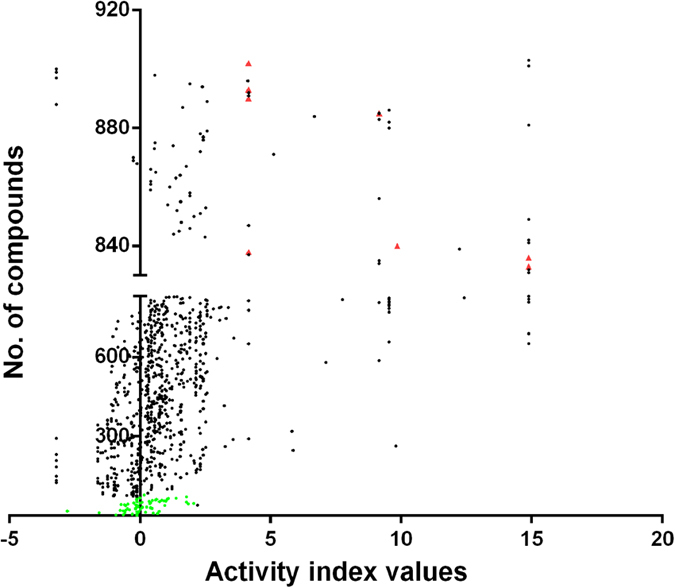
The AIs of 903 constituents. Green dots represented 72 constituents in the whole extract of GZJSYT; red triangles represented eight potential active constituents that were isolated by semi-preparative LC.

The AIs of the constituents in GZJSYT were calculated according to the equation (1). As shown in Fig. 5, 61 constituents had higher AIs, which existed mainly in fractions E15, E16, E17, and E18. The greater the AI value is, the more likely the constituent is active^1^. Therefore, the 61 constituents were speculated to have anti-inflammatory activities.

### Isolation and identification of eight potential active constituents

In order to verify the predicted results of AI method, eight constituents with higher AI values were isolated from *Glycyrrhizae radix et rhizoma preparata cum melle* with semi-preparative LC guided by LC-MS. Based on LC-MS, ^1^H and ^13^C NMR (Supplementary information), they were identified as glycycoumarin (833), glycyrrhisoflavone (836), euchrestaflavanone A (838), licoisoflavone A (840), licochalcone A (883), licoisoflavone B (890), glisoflavanone (893), and isoangustone A (902). Their chemical structures are shown in Fig. 2B.

### Validation of anti-inflammatory activities of eight potential active constituents

**Figure 6:**
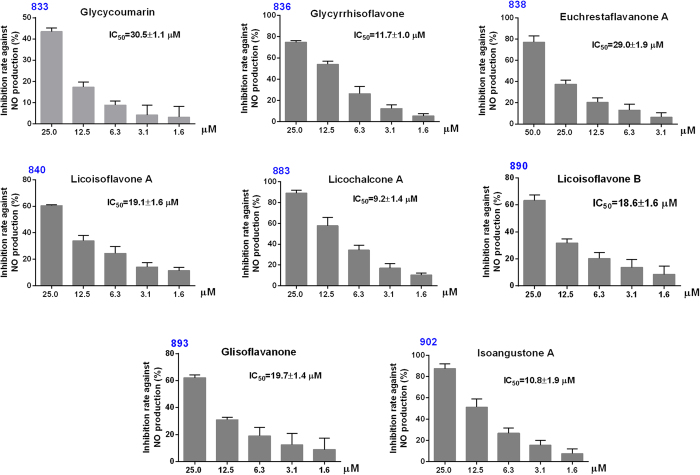
The inhibition rates against NO production of eight isolated compounds in different concentrations. 833: glycycoumarin, 836: glycyrrhisoflavone, 838: euchrestaflavanone A, 840: licoisoflavone A, 883: licochalcone A, 890: licoisoflavone B, 893: glisoflavanone, and 902: isoangustone A.

The anti-inflammatory activities of eight constituents were determined using LPS-induced RAW 264.7 macrophages at a series of concentrations without cytotoxicity. The results are shown in Fig. 6. Glycycoumarin (833), glycyrrhisoflavone (836), euchrestaflavanone A (838), licoisoflavone A (840), licochalcone A (883), licoisoflavone B (890), glisoflavanone(893), and isoangustone A (902) possessed IC_50_ values of 30.5 ± 1.1 μM, 11.7 ± 1.0 μM, 29.0 ± 1.9 μM, 19.1 ± 1.6 μM, 9.2 ± 1.4 μM, 18.6 ± 1.6 μM, 19.7 ± 1.4 μM, and 10.8 ± 1.9 μM, respectively. Compared with indomethacin (positive control, IC_50_: 31.6 ± 2.0 μM)^22^, eight compounds all had high inhibition rates against NO production. Among them, glycycoumarin (833), euchrestaflavanone A (838), licoisoflavone A (840), licochalcone A (883) and licoisoflavone B (890) had been reported to have anti-inflammatory activity^23^,^24^,^25^, but glycyrrhisoflavone (836), glisoflavanone (893), and isoangustone A (902) were reported to have anti-inflammatory activity for the first time in this study.

## Discussion

The chemical profile of GZJSYT was firstly reported in this work. The study of fragmentation rules of MS^n^ spectra for different types of constituents was critical for the rapid identification of their chemical structures by LC-MS. The fragment pathway of monoterpenoids, the main constituents in GZJSYT, was investigated in detail.It was found that paeoniflorin type monoterpenoids mainly eliminated HCHO due to the unstable hemiketal group of pinane skeleton in MS^n^ spectra, and then fragmented the substituent group R_2_, such as BA and Glc, from the mother skeleton under higher collision energy to form typical fragment ion at *m/z* 165. However, albiflorin type monoterpenoids firstly fragmented the substituent group R_2_, and then lost the lactone group of pinane skeleton under higher collision energy, which was different from the fragmentation behavior of paeoniflorin type monoterpenoids.

Prefraction was not only helpful to decrease the chemical complex of TCM formulae, but also necessary to find the trace constituents that had significant activity. In this work, 903 constituents were detected in GZJSYT fractions while only 72 constituents in the whole extract of GZJSYT. Considering that the identification of chemical structures of those 903 constituents in the fractions was time-consuming even by LC-MS, only the potential active constituents were isolated and identified unambiguously by NMR.

Among the 61 fractions, 40 fractions had anti-inflammatory effects. Many constituents with positive AI values might have contributions to the anti-inflammatory effects. The low inhibition rate against NO production of A01 might be due to the weak activity of constituents with higher contents. However, in fractions, the trace active constituents were enriched, so many fractions had higher inhibition rate against NO production.

**Figure 7:**
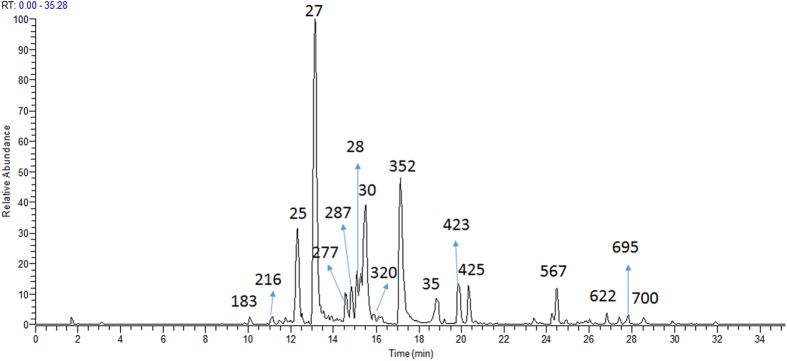
The total ion chromatograms (TICs) of the fraction D05 by LC–IT–MS in negative ion mode.

These active fractions contained hundreds of constituents. It was not easy to find the active constituents rapidly without the guidance of AI method. It is generally considered that the main constituents in active fraction may be active, but sometime it is not the case. Here we take the active fractions D05 as an example. Fractions D05 exhibited 53% of inhibition rate against NO production. The LC-MS chromatogram of D05 is shown in Fig. 7. It was found that paeoniflorin (27) was the main constituent of fraction D05. It was easy to think that paeoniflorin (27) might be active. As shown in Table 2, the AI value of paeoniflorin (27) was −0.18. According to our previous work^1^, negative AI value indicated that the constituents might have no anti-inflammatory activity or weak activity. In fact, paeoniflorin (27) was proved experimentally to have no anti-inflammatory activity at 50 μM, only had weak anti-inflammatory effect at 200 μM (NO inhibition rate of 38%)^26^. The result indicated the contribution of paeoniflorin (27) to the anti-inflammatory of fraction D05 was weak. Therefore, activity index could predict the probability of the constituents being active to some extent.

The AI results indicated that 61 constituents with higher AI values might have anti-inflammatory activities, and most of them were from *Glycyrrhizae radix etrhizoma*. The structures of eight active constituents were confirmed by NMR, which included one coumarin and seven flavonoids. Interestingly, most of their structures contained isopentene groups, which might be important for their significant anti-inflammatory activities.

## Conclusion

In summary, an approach based on AI, LC-MS, and NMR was developed to rapidly discover and identify the anti-inflammatory constituents from GZJSYT in this study. A total number of 903 constituents were detected in the whole extract and fractions by LC-MS. The AI predicted results showed that 61 constituents with higher AIs were very likely to have anti-inflammatory activities. Eight potential active constituents of them were validated to have significant anti-inflammatory activity in LPS-induced RAW 265.7 macrophages. And more importantly, three of them were first reported to have anti-inflammatory activity in this work.

Calculating activity index of constituents requirs the global analysis of the fractions from TCM formulae, not just the active fractions, which is different from the traditional activity-guided method that only focuses in active fractions. The results indicated that the approach was efficient and feasible. This approach would be generally applicable for the rapid discovery of anti-inflammatory constituents from other TCMs or medical plants.

## Methods

### Materials and reagents

*Paeoniae radix alba*, *Cinnamomi ramulus*, *Jujubae fructus* and *Glycyrrhizae radix etrhizoma preparata cum melle*, were obtained from Zhejiang Chinese Medical University Medical Pieces Co., Ltd. (Hangzhou, China), and *Zingiberis rhizome recens* was purchased from the local market.

The sources of reference standards were as follows. Gallic acid and citric acid were purchased from Sinopharm Chemical Reagent Co., Ltd. (Shanghai, China). Succinic acid was acquired from Sangon Biotech (Shanghai) Co., Ltd. (Shanghai, China). Liquiritin apioside and liquiritigenin were obtained from Shanghai Winherb Medical Technology Co., Ltd. (Shanghai, China). Paeoniflorin was obtained from Aladdin Reagent Co., Ltd. (Shanghai, China). Liquiritin, ononin, glycyroside, liquiritigenin-7-*O*-*β*-D-apiofuranosyl-4′-*O*-*β*-D-glucopyranoside, macedonoside A, uralsaponin A, licorice saponin G_2_, licorice saponin H_2_, and licorice saponin A_3_ were isolated from *Glycyrrhizae radix et rhizoma* in our laboratory, and their structures were identified by UV, MS, and NMR. The purity of the each reference standard was greater than 96%.

HPLC grade of acetonitrile and methanol were purchased from Merck (Darmstadt, Germany). Formic acid (HPLC grade) was obtained from ROE Scientific Inc. (Newark, DE, USA). Deionized water was purified by Milli-Q system from Millipore (Molsheim, France). Acetonitrile for preparative HPLC was purchased from Amethyst Chemicals (J&KScientific Ltd., Beijing, China). Anhydrous ethanol and 95% ethanol were purchased from Shanghai Lingfeng Chemical Co., Ltd. (Shanghai, China) and Zhejiang Changqing Chemical Co., Ltd. (Hangzhou, China), respectively. D101 macroporous resin was bought from Tianjin Haiguang Chemical Co. Ltd. (Tianjin, China).

Dulbecco’s modified Eagle’s medium (DMEM), Fetal Bovine Serum (FBS), trypsin-EDTA and the penicillin-streptomycin were purchased from Gibico BRL (Grand Island, NY, USA). Lipopolysaccharides (LPS), dimethylsulfoxide (DMSO) and indomethacin (purity ≥ 99%) were acquired from Sigma-Aldrich (St. Louis, MO, USA).

### Preparation of sample and standard solutions

According to the recipe of GZJSYT in Shang-Han-Lun, *Cinnamomi ramulus* (27.3 g), *Paeoniae radix alba* (54.6 g), *Zingiberis rhizoma recens* (27.3 g), *Jujubae fructus* (72.7 g), and *Glycyrrhizae radix et rhizoma preparata cum melle* (18.2 g) were immersed in 1.2 L water (6-fold) overnight. Then, they were extracted twice for 1 h of each time and the filtrates were combined. Subsequently, the combined solution was concentrated under reduced pressure using a rotary evaporator, and the residue was dissolved in appropriate volume of water. Finally, the solution was analyzed by LC-MS after it was filtered through 0.22 μm filter membrane.

The fractions of GZJSYT were prepared as previously reported^1^. Firstly, the extract of GZJSYT was loaded onto a glass column (4.6 cm × 30 cm) packed with the D101 macroporous resin. The column was rinsed with H_2_O first to get fraction B01, and then in turn eluted with 20% ethanol, 40% ethanol, 95% ethanol to give fractions B02, B03, and B04, respectively. Fractions B02, B03 and B04 were subject to preparative HPLC to obtain new subfractions. Subfractions preparation was carried out on a 1200 series LC system (Agilent, Palo Alto, USA) and a Zorbax SB-C18 column (21.2 mm × 250 mm, 7 μm, Agilent) with the mobile phase consisted of A (H_2_O) and B (acetonitrile) at a flow rate of 10 mL/min. Subfractions were collected each three minutes from the fifth minute to the sixty-fifth minute. The elute gradient used for fraction B02 was as follows: 0 min, 3% B; 50 min, 20% B; 64 min, 50% B; 67 min, 100% B; 70 min, 100% B. The obtained subfractions were named as fractions C01-C20. Fraction B03 was applied to preparative HPLC to yield fractions D01-D20 with the following gradient elution: 0 min, 15% B; 40 min, 30% B; 55 min, 45% B; 60 min, 80% B; 64 min, 100% B; 70 min, 100% B. The elute gradient used for fraction B04: 0 min, 20% B; 20 min, 30% B; 40 min, 45% B; 55 min, 90% B; 60 min, 100% B; 70 min, 100% B. The obtained subfractions were named as fractions E01-E20. All the fractions were concentrated and freeze-dried. Finally, the samples were used for LC-IT-MS and evaluated on anti-inflammatory activity except the fractions D20, E19, E20 due to the very low amount of them.

Reference standards were dissolved in methanol at the concentration of about 0.1 mg/mL and stored at 4 °C. They were filtered through 0.22 μm filter membrane before use.

### LC-IT-MS conditions

HPLC was performed on an Agilent 1100 HPLC system (Agilent, Waldbronn, Germany) containing a binary pump, an auto-sampler, a column compartment and a photo-diodearray (DAD) detector. Separation was carried out on a Zorbax SB-C18 Rapid Resolution HT column (4.6 mm × 50 mm, 1.8 μm, Agilent) at a flow rate of 0.6 mL/min and column temperature of 30 °C. The mobile phase was prepared using two solutions, A and B, where A was 0.05% formic acid in water, and B was acetonitrile. The gradient elution was optimized as follows: 0 min, 3% B; 35 min, 41% B; 40 min, 100% B; 50 min 100% B. The injection volume was 3 μL. The DAD detector scanned range 190–400 nm, and the samples were detected at 254 and 280 nm.

IT-MS analysis was operated on a Finnigan LCQ Deca XP^plus^ ion trap mass spectrometer (Thermo Finnigan, San Jose, CA, USA) with ESI interface and an ion trap mass (IT-MS) analyzer. The analysis was performed on both negative (ESI^−^) and positive (ESI^+^) ion modes under the following operation parameters: auxiliary/sweep gas (N_2_), 20 L/min; sheath gas (N_2_), 60 L/min; collision gas (He); ESI spray voltage, −3 kV (ESI^−^) and 4 kV (ESI^+^); capillary voltage,−15 V (ESI^−^) and 19 V (ESI^+^); capillary temperature, 350 °C; tube lens offset voltage, −30 V (ESI^−^) and 25 V (ESI^+^); scan range, *m/z* 100–1500. The collision energy was set at 35–45% to give appropriate fragmentations.

### LC-Q-TOF-MS conditions

LC-Q-TOF-MS analysis was manipulated on an Acquity^TM^ ultra performance LC system (Waters Corp., Milford, MA, USA) coupled with a high resolution Triple TOF 5600^+^ (AB SCIEX, Framingham, MA, USA), which was equipped with an ESI source. The chromatographic separation conditions were the same as that in LC-IT-MS. The sample was analyzed in both positive and negative mode, and the parameters in the source were set as follows: curtion gas (CUR), 30 psi; ion source GS1, 50 psi; ion source GS2, 50 psi; source temperature, 550 °C (ESI^−^) and 600 °C (ESI^+^); ionspray voltage floating (ISVF), −4500 V(ESI^−^) and 5500 V (ESI^+^); collision energy (CE), 10 V; declustering potential (DP), ±100 V; ion release delay (IRD) at 67; ion release width (IRW) at 25. The mass range was *m/z* 100–1500. The accurate mass acquired were processed by means of the elemental composition calculator incorporated in the PeakView^®^ software (AB SCIEX).

### Isolation and identification of potential active constituents

The *Glycyrrhizae radix et rhizoma preparata cum melle* (5 kg) was extracted with 95% ethanol (8-fold) for 1 h at the first time. Then the extraction was repeated with 95% ethanol (6-fold) for 1 h. The two extracts were mixed together, and condensed to appropriate concentration for separation experiments. The extract was loaded on a glass column (10 cm × 250 cm) packed with the D101 macroporous resin, and eluted with H_2_O, 40% ethanol and 95% ethanol, respectively. Effluent of 95% ethanol was collected and condensed to appropriate volume. Subsequently, the sample was further separated by medium-pressure column chromatography (10 cm × 50 cm) loaded with ODS. The mobile phases consisted of H_2_O (A) and methanol (B) and the flow speed at 60 mL/min. Isocratic elution with 55% B was carried out, and the eluate was collected and dried for semi-preparation.

Semi-preparation was carried out on an Agilent 1100 equipped with a degasser, a quaternary pump, an auto-sampler, a column compartment and a UV detector. The Zorbax SB-C18 column (Semi-Preparative, 9.4 mm × 250 mm, 5 μm, Agilent) at a column temperature of 30 °C was utilized and eluted with the gradient profile of A (water) and B (acetonitrile) at a flow rate of 3.0 mL/min. the gradient elution was: 0 min, 15% B; 40 min, 34% B; 80 min. 37% B; 120 min, 40% B; 150 min, 45% B; 160 min, 48% B; 205 min, 54% B; 206 min, 100% B; 216 min, 100% B. The injection volume was 100 μL and UV detection wavelength at 230 nm. NMR experiments of isolated compounds were dissolved in methanol-*d*_*4*_ and performed on a Bruker Avance III 500 NMR spectrometer (^1^H: 500 MHz, ^13^C: 125 MHz; Bruker Corporation, Billerica, MA, USA).

### Evaluation of the anti-inflammatory activity

The murine macrophage cell line RAW 264.7 (the Type Culture Collection of the Chinese Academy of Sciences, Shanghai, China) was cultured in 90% Dulbecco’s Modification Eagle’s Medium (DMEM) with 4.5 g/L glucose, L-glutamine and sodium pyrurate supplemented with 10% inactivated Fetal Bovine Serum (FBS) and 1% 10000 U/mL of penicillin, 10000 μg/mL of streptomycin at 37 °C in a 5% CO_2_ humidified atmosphere.

To determine the cytotoxicity of each constituent, RAW 264.7 cells (5 × 10^4^ cells/mL, 100 μL/well) plated in 96 well plates were cultured for 24 h at 37 °C. The samples dissolved in DMSO were diluted with DMEM supplemented with 10% inactivated FBS, penicillin G (100 U/mL) and streptomycin (100 μg/mL) before co-incubated with the cells for 24 h. Then, MTT solution (100 μL, 0.5 mg/mL in per well) was added and incubated for 4 h. The solution in per well was drawn and the formazan crystals were adequately dissolved in DMSO (100 μL/well) by shaking for 10 min at 37 °C. The absorbance at 580 nm was measured in a microplate reader (Bio-Tek ELX800, Winooski, VT, USA). Fresh culture medium was used as a blank in every experiment. The experiments were implemented for three times in parallel.

For the determination of the anti-inflammatory activity, RAW 264.7 cells were seeded into a 96-well plate at a density of 2 × 10^4^ cells/well for 24 h. Then, the fresh culture medium containing samples and LPS (200 ng/mL) were added to each well (140 μL/well) and further incubated for 24 h. Subsequently, 100 μL of supernatant from each well was mixed with 100 μL of Griess reagent (0.5% sulfanilamide and 0.05% naphthylene diamide dihydrochloride in 2.5% H_3_PO_4_) in a separate 96-well plate. After an incubation of 10 min, the absorbance of NO was determined at 535 nm with a microplate reader. Indomethacin (50 μΜ) was adopted as a positive control and fresh culture medium was used as the blank in all experiments. The experiments were carried out for three times in parallel.

### Calculation of AI values of constituents

The AI values of constituents were calculated using mathematical equations reported in the previous work^1^.


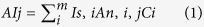


*AI*_*j*_ standed for AI value of constituent *j*.

*I*_*s,i*_ represented standardized value of NO inhibition rate of fraction *i*, it was defined as 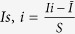. *I*_*i*_ standed for value of NO inhibition rate of fraction *i*; *Ī* represented mean value of NO inhibition rate of all fractions; *S* standed for standard deviation of NO inhibition rate of all fractions.

*A*_*n,i,j*_ represented normalized value of peak area of constituent *j* in fraction *I*, it was defined as 
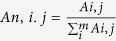
. *A*_*i,j*_ was peak area of constituent *j* in fraction *i*; *m* was the numbers of fractions in the experiment.

*C*_*i*_: weight of the anti-inflammation concentration of fraction *i*, it was defined as *C*_*i*_ = *c*_*max*_*/c*_*i*_. *c*_*max*_ was the highest value of the anti-inflammation concentrations of fractions, and *c*_*i*_ was the anti-inflammation concentrations of fraction *i*.

## Additional Information

**How to cite this article**: Wang, S. *et al.* Rapid discovery and identification of anti-inflammatory constituents from traditional Chinese medicine formula by activity index, LC-MS, and NMR. *Sci. Rep.* **6**, 31000; doi: 10.1038/srep31000 (2016).

## Supplementary Material

Supplementary Information

## Figures and Tables

**Table 1:** The constituents detected in the whole extract of GZJSYT by LC-Q-TOF-MS.

No	t_R_ (min)	Identification	Molecular Formula	Detected ions (*m/z*)	Error (ppm)	Source
1	1.15	Malic acid	C_4_H_6_O_5_	133.0154 [M−H]^−^	8.7	GPJZC
2	1.25	Unknown	—	—	—	G
3	1.53	Unknown	—	—	—	PJ
4*	1.59	Citric acid	C_6_H_8_O_7_	191.0208 [M−H]^−^	5.6	GPJZC
5	1.71	Desbenzoyl paeoniflorin	C_16_H_24_O_10_	421.1344 [M + HCOO]^−^	−1.8	P
6*	2.07	Succinic acid	C_6_H_4_O_6_	—	—	GPJZC
7	2.15	Isomaltodebenzoyl paeoniflorin	C_22_H_34_O_15_	583.1876 [M + HCOO]^−^	−0.6	P
8*	3.26	Gallic acid	C_7_H_6_O_5_	169.0157 [M−H]^−^	8.6	P
9	3.95	1-*O*-*β*-D-Gucopyranosyl paeonisuffrone	C_16_H_24_O_9_	405.1404 [M + HCOO]^−^	0.4	P
10	4.16	Glucogallin	C_13_H_16_O_10_	331.0669 [M−H]^−^	−0.5	P
11	5.57	6-Galloylsucrose or 6′-Galloylsucrose or 1′-Galloylsucrose	C_19_H_26_O_15_	493.1197 [M−H]^−^	0.4	P
12	7.18	6-*O*-*β*-D-glucopyranosyl lactinolide	C_16_H_26_O_9_	407.1559 [M + HCOO]^−^	0	P
13	8.46	6′-*O*-Galloyldesbenzoyl paeoniflorin	C_23_H_28_O_14_	527.1403 [M−H]^−^	0.6	P
14	8.73	(*p*-Hydroxybenzyl)malonic acid	C_10_H_10_O_5_	209.0465 [M−H]^−^	4.6	G
15	9.67	Zizybeoside I or its isomer	C_19_H_28_O_11_	477.1613 [M + HCOO]^−^	−0.1	J
16	9.74	Unknown	C_16_H_26_O_8_	391.1611 [M + HCOO]^−^	0.3	P
17	10.32	Zizybeoside I or its isomer	C_19_H_28_O_11_	477.1603 [M + HCOO]^−^	−2.2	J
18	10.32	Oxypaeoniflorin	C_23_H_28_O_12_	541.1560 [M + HCOO]^−^	−0.5	P
19	11.09	Zizybeoside II	C_25_H_38_O_16_	639.2147 [M + HCOO]^−^	0.8	J
20	11.81	Unknown	—	—	—	P
21	11.85	6′-*O*-*β*-Glucopyranosyl albiflorin or its isomer	C_29_H_38_O_16_	687.2152 [M + HCOO]^−^	1.5	P
22	12.51	Unknown	C_35_H_48_O_21_	849.2685 [M + HCOO]^−^	1.8	P
23	12.69	6′-*O*-*β* -Glucopyranosyl albiflorin or its isomer	C_29_H_38_O_16_	687.2152 [M + HCOO]^−^	1.5	P
24	12.95	Isomaltopaeoniflorin	C_29_H_38_O_16_	687.2146 [M + HCOO]^−^	0.6	P
25	13.19	Albiflorin	C_23_H_28_O_11_	525.1616 [M + HCOO]^−^	0.4	P
26	13.53	6′-*O*-*β*-Glucopyranosyl albiflorin or its isomer	C_29_H_38_O_16_	687.2152 [M + HCOO]^−^	1.5	P
27*	14.21	Paeoniflorin	C_23_H_28_O_11_	525.1619 [M + HCOO]^−^	1.0	P
28*	16.7	Liquiritigenin-7-*O*-*β*-D-apiofuranosyl-4′-*O*-*β*-D-glucopyranoside	C_26_H_30_O_13_	549.1620 [M−H]^−^	1.2	G
29*	16.75	Liquiritin	C_21_H_22_O_9_	419.1338 [M + H]^+^	0.3	G
30*	17.06	Liquiritin apioside	C_26_H_30_O_13_	549.1625 [M−H]^−^	2.1	G
31*	17.09	Liquiritigenin	C_15_H_12_O_4_	257.0812 [M + H]^+^	1.4	G
32	17.93	Galloylalbiflorin	C_30_H_32_O_15_	631.1674 [M−H]^−^	0.9	P
33	18.13	Apigenin 6-*C*-*β*-xylopyranosyl-8-*C*-(6″′-O-(3-hydroxy-3-methylglutaroyl)-*β*-glucopyranoside)	C_32_H_36_O_18_	707.1842 [M−H]^−^	1.9	G
34	18.28	Unknown	—	—	—	—
35	20.55	Albiflorin R_1_	C_23_H_28_O_11_	525.1608 [M + HCOO]^−^	−1.1	P
36*	21.53	Glycyroside	C_27_H_30_O_13_	607.1684 [M + HCOO]^−^	2.6	G
37*	22.17	Ononin	C_22_H_22_O_9_	475.1254 [M + HCOO]^−^	1.7	G
38	23.54	Sacranoside A or its isomer	C_21_H_34_O_10_	491.2143 [M + HCOO]^−^	1.8	P
39	24.53	Sacranoside A or its isomer	C_21_H_34_O_10_	491.2143 [M + HCOO]^−^	1.8	P
40	26.25	Macedonoside A isomer	C_42_H_62_O_17_	837.3939 [M−H]^−^	3.0	G
41	26.52	24-Hydroxy-licorice-saponin A_3_	C_48_H_72_O_22_	999.4474 [M−H]^−^	3.2	G
42	27.08	24-Hydroxy-licorice-saponin A_3_ isomer	C_48_H_72_O_22_	999.4489 [M−H]^−^	4.7	G
43	27.47	22-Hydroxy-licorice-saponin G_2_	C_48_H_72_O_21_	853.3895 [M−H]^−^	3.2	G
44	27.99	Benzoylpaeoniflorin	C_30_H_32_O_12_	629.1891 [M + HCOO]^−^	2.4	P
45*	28.4	Licorice saponin A_3_	C_48_H_72_O_21_	983.4535 [M−H]^−^	4.2	G
46	28.62	Albiflorin R_3_	C_30_H_32_O_12_	629.189 [M + HCOO]^−^	2.3	P
47	29.53	Yunganoside K_1_ or its isomer	C_48_H_72_O_21_	983.4535 [M−H]^−^	3.2	G
48*	29.84	Macedonoside A	C_42_H_62_O_17_	837.3951 [M−H]^−^	4.4	G
49	30.16	Uralsaponin R/S	C_48_H_74_O_20_	969.4723 [M−H]^−^	2.3	G
50	30.60	Yunganoside K_2_ or its isomer	C_42_H_62_O_17_	837.3950 [M−H]^−^	4.3	G
51	30.66	Uralsaponin P	C_42_H_64_O_16_	823.4147 [M−H]^−^	3.1	G
52	31.4	Unknown	C_16_H_32_O_5_	303.2181 [M−H]^−^	1.3	J
53*	31.99	Licorice saponin G_2_	C_42_H_62_O_17_	837.3950 [M−H]^−^	4.3	G
54	32.77	Yunganoside L_1_/J_1_	C_48_H_72_O_20_	967.4576 [M−H]^−^	3.3	G
55	32.96	Yunganoside K_2_ or its isomer	C_42_H_62_O_17_	837.3954 [M−H]^−^	4.7	G
56	33.22	Yunganoside G_1_	C_48_H_74_O_21_	985.4688 [M−H]^−^	3.9	G
57	33.5	Yunganoside K_2_ or its isomer	C_42_H_62_O_17_	837.3954 [M−H]^−^	4.5	G
58	33.79	Yunganoside G_2_ or its isomer	C_42_H_64_O_17_	839.4105 [M−H]^−^	4.1	G
59*	34.03	Uralsaponin A	C_42_H_62_O_16_	821.4012 [M−H]^−^	5.7	G
60	34.04	Unknown	C_30_H_44_O_3_	453.3362 [M + H]^+^	0.3	J
61	35.13	Yunganoside L_1_/J_1_	C_48_H_72_O_20_	967.4590 [M−H]^−^	4.7	G
62	35.37	Albiziasaponin B	C_48_H_74_O_20_	969.4744 [M−H]^−^	4.5	G
63*	35.7	Licorice saponin H_2_	C_42_H_62_O_16_	821.4009 [M−H]^−^	5.3	G
64	35.7	Unknown	C_30_H_44_O_3_	453.3364 [M + H]^+^	0.2	J
65	36.27	Glycyrrhizic acid isomer	C_42_H_62_O_16_	821.4009 [M−H]^−^	5.3	G
66	38.09	Licorice saponin C_2_ or its isomer	C_42_H_62_O_15_	805.4056 [M−H]^−^	5.0	G
67	38.59	Actinopolysporin B	C_16_H_30_O_4_	285.2077 [M−H]^−^	—	—
68	39.21	Unknown	—	—	—	C
69	39.48	Unknown	—	—	—	C
70	39.66	Unknown	—	—	—	—
71	39.82	Unknown	—	—	—	C
72	40.06	Unknown	—	—	—	P

t_R_: retention time; G: *Glycyrrhizae radix et rhizoma preparata cum melle*; P: *Paeoniae radix alba*; J: *Jujubae fructus*; C: *Cinnamomi ramulus*; Z: *Zingiberis rhizoma recens*.

^*^Compared with reference standards.

**Table 2:** Activity indexes of compounds in the fraction D05 of GZJSYT.

No	t_R_ (min)	Detected *m/z*	Compounds	AIs
183	10.07	635.2^a^	1,2,6-Tri-*O*-galloyl-*β*-D-glucose	0.76
216	11.14	635.3^a^	1,3,6-Tri-*O*-galloyl-*β*-D-glucose	0.38
25	12.29	525.3^b^	Albiflorin	0.00
27*	14.15	525.2^b^	Peoniflorin	−0.18
277	14.55	405.2^a^	Unknown	0.20
287	14.82	787.1^a^	1,2,3,6-Tetra-*O*-galloyl-*β*-D-glucose	0.50
28*	15.15	595.0^b^	Liquiritigenin-7-*O*-*β*-D-apiofuranosyl-4′-*O*-*β*-D-glucopyranoside	0.46
320	15.81	786.9^a^	1,2,4,6-Tetra-*O*-galloyl-*β*-D-glucose	0.75
352	17.15	939.2^a^	Pentagalloylglucose	1.14
35	18.82	525.1^b^	Albiflorin R_1_	0.07
423	19.84	549.3^a^	Licuraside	0.49
425	20.33	549.3^a^	2′-O-[*β*-D-Apiofuranosyl-(1→2)-*β*-D-glucopyranosyl] isoliquiritigenin	0.54
567	24.5	999.7^a^	24-Hydroxy-licorice- saponin A_3_ isomer	0.48
622	26.82	837.5^a^	Macedonoside A isomer	−0.22
695	27.84	969.6^a^	Albiziasaponin B isomer	0.66
700	28.53	823.5^a^	Uralsaponin P isomer	0.72

^a^[M−H]^−^.

^b^[M + HCOO]^−^.

^*^Compared with reference standards.

## References

[b1] Wang S. F. *et al.* Identification of the effective constituents for anti-inflammatory activity of Ju-Zhi-Jiang-Tang, an ancient traditional Chinese medicine formula. J. Chromatogr. A 1348, 105–124 (2014). 24813935 10.1016/j.chroma.2014.04.084

[b2] Jiang Z. *et al.* Dose-dependent targeted knockout methodology combined with deep structure elucidation strategies for Chinese licorice chemical profiling. J. Pharm. Biomed. Anal. 115, 130–137 (2015). 26186616 10.1016/j.jpba.2015.06.020

[b3] Wang S. F. *et al.* Identification of chemical constituents in two traditional Chinese medicine formulae by liquid chromatography-mass spectrometry and off-line nuclear magnetic resonance. J. Pharm. Biomed. Anal. 117, 255–265 (2016). 26397205 10.1016/j.jpba.2015.09.007

[b4] Yang W. Z. *et al.* A strategy for efficient discovery of new natural compounds by integrating orthogonal column chromatography and liquid chromatography/mass spectrometry analysis: Its application in Panax ginseng, Panax quinquefolium and Panax notoginseng to characterize 437 potential new ginsenosides. Anal. Chim. Acta 739, 56–66 (2012). 22819050 10.1016/j.aca.2012.06.017

[b5] Yang W. Z. *et al.* Rapid chemical profiling of saponins in the flower buds of Panax notoginseng by integrating MCI gel column chromatography and liquid chromatography/mass spectrometry analysis. Food Chem. 139, 762–769 (2013). 23561171 10.1016/j.foodchem.2013.01.051

[b6] Saitoh K. *et al.* Effects of Keishi-ka-shakuyaku-to (Gui-Zhi-Jia-Shao-Yao-Tang) on diarrhea and small intestinal movement. Biol. Pharm. Bull. 22, 87–89 (1999). 9989669 10.1248/bpb.22.87

[b7] Yuan S. Q., Xia L. N. & Guo Y. L. Experimental study on the dose-effect relationship of Gui-Zhi-Tang and Gui-Zhi-Jia-Shao-Yao-Tang. Nei Mongol Zhong Yi Yao 33, 99–99 (2014).

[b8] Li Z. *et al.* Derivative multiple reaction monitoring and single herb calibration approach for multiple components quantification of traditional Chinese medicine analogous formulae. J. Chromatogr. A 1376, 126–142 (2015). 25541090 10.1016/j.chroma.2014.12.024

[b9] Yan Y. *et al.* HPLC-DAD-Q-TOF-MS/MS analysis and HPLC quantitation of chemical constituents in traditional Chinese medicinal formula Ge-Gen Decoction. J. Pharm. Biomed. Anal. 80, 192–202 (2013). 23584078 10.1016/j.jpba.2013.03.008

[b10] Li R., Wang X. L., Zhou Y., Cai M. & Ding L. S. Analysis of sodium adduct paeoniflorin, albiflorin and their derivatives by (+)ESI-MSn, DFT calculations and computer-aided mass spectrometry analysis program. J. Mass Spectrom. 42, 335–345 (2007). 17199256 10.1002/jms.1164

[b11] Chen L. L., Qi J., Chang Y. X., Zhu D. N. & Yu B. Y. Identification and determination of the major constituents in Traditional Chinese Medicinal formula Danggui-Shaoyao-San by HPLC-DAD-ESI-MS/MS. J. Pharm. Biomed. Anal. 50, 127–137 (2009). 19411155 10.1016/j.jpba.2009.03.039

[b12] Qi Y. *et al.* Chemical profiling of Wu-tou decoction by UPLC-Q-TOF-MS. Talanta 118, 21–29 (2014). 24274266 10.1016/j.talanta.2013.09.054

[b13] Fu Q., Wang S. B., Zhao S. H., Chen X. J. & Tu P. F. Three new monoterpene glycosides from the roots of Paeonia lactiflora. J. Asian Nat. Prod. Res. 15, 697–702 (2013). 23659613 10.1080/10286020.2013.794420

[b14] Liang J. *et al.* The profiling and identification of the absorbed constituents and metabolites of Paeoniae Radix Rubra decoction in rat plasma and urine by the HPLC-DAD-ESI-IT-TOF-MSn technique: A novel strategy for the systematic screening and identification of absorbed constituents and metabolites from traditional Chinese medicines. J. Pharm. Biomed. Anal. 83, 108–121 (2013). 23727363 10.1016/j.jpba.2013.04.029

[b15] Li S. L. *et al.* Chemical profiling of Radix Paeoniae evaluated by ultra-performance liquid chromatography/photo-diode-array/quadrupole time-of-flight mass spectrometry. J. Pharm. Biomed. Anal. 49, 253–266 (2009). 19097720 10.1016/j.jpba.2008.11.007

[b16] Shim S. B., Kim N. J. & Kim D. H. beta-glucuronidase inhibitory activity and hepatoprotective effect of 18 beta-glycyrrhetinic acid from the rhizomes of Glycyrrhiza uralensis. Planta Med. 66, 40–43 (2000). 10705732 10.1055/s-2000-11109

[b17] Song W. *et al.* Uralsaponins M-Y, Antiviral Triterpenoid Saponins from the Roots of Glycyrrhiza uralensis. J. Nat. Prod. 77, 1632–1643 (2014). 24957203 10.1021/np500253m

[b18] Zheng Y. F. *et al.* Hepatoprotective Triterpene Saponins from the Roots of Glycyrrhiza inflata. Molecules 20, 6273–6283 (2015). 25859783 10.3390/molecules20046273 PMC6272637

[b19] Wang S. F. *et al.* Fragment ion diagnostic strategies for the comprehensive identification of chemical profile of Gui-Zhi-Tang by integrating high-resolution MS, multiple-stage MS and UV information. J. Pharm. Biomed. Anal. 98, 22–35 (2014). 24879517 10.1016/j.jpba.2014.05.013

[b20] Wang S. F., Chen P. H., Xu Y. M., Li X. D. & Fan X. H. Characterization of the chemical constituents in Da-Huang-Gan-Cao-Tang by liquid chromatography coupled with quadrupole time-of-flight tandem mass spectrometry and liquid chromatography coupled with ion trap mass spectrometry. J. Sep. Sci. 37, 1748–1761 (2014). 24771648 10.1002/jssc.201400061

[b21] Wang S. *et al.* Identification of Chemical Constituents in the Extract and Rat Serum from Ziziphus Jujuba Mill by HPLC-PDA-ESI-MSn. Iran J. Pharm. Res. 13, 1055–1063 (2014). 25276208 PMC4177628

[b22] Wang S. F., Xu Y. M., Jiang W. & Zhang Y. F. Isolation and Identification of Constituents with Activity of Inhibiting Nitric Oxide Production in Raw 264.7 Macrophages from Gentiana triflora. Planta Med. 79, 680–686 (2013). 23599008 10.1055/s-0032-1328460

[b23] Fu Y., Chen J., Li Y. J., Zheng Y. F. & Li P. Antioxidant and anti-inflammatory activities of six flavonoids separated from licorice. Food Chem. 141, 1063–1071 (2013). 23790887 10.1016/j.foodchem.2013.03.089

[b24] Kolbe L. *et al.* Anti-inflammatory efficacy of Licochalcone A: correlation of clinical potency and *in vitro* effects. Arch. Dermatol. Res. 298, 23–30 (2006). 16552540 10.1007/s00403-006-0654-4

[b25] Tashiro M. *et al.* Effects of isoflavones from Sophora species on the growth and activation of a mouse macrophage-like cell line. Anticancer Res. 22, 2185–2191 (2002). 12174902

[b26] Chen L. L. In Study on the chemical constituents of Gui-Zhi-Tang by LC-MS and evaluation of its anti-inflammatory activity. (Master’s Thesis) 45–49 (Zhejiang University, 2015).

