# Supplementary material for: Rapid discovery and identification of anti-inflammatory constituents from traditional Chinese medicine formula by activity index, LC-MS, and NMR
Source: Sci Rep. 2016 Aug 8;6:31000. doi: 10.1038/srep31000 (PMC4976346; doi:10.1038/srep31000)
Supplement: Supplementary Information [file srep31000-s1.doc]

**Rapid discovery and identification of anti-inflammatory constituents from traditional Chinese medicine formula by activity index, LC-MS, and NMR**

Shufang Wang*, Haiqiang Wang, Yining Liu, Yi Wang, Xiaohui Fan, Yiyu Cheng

Pharmaceutical Informatics Institute, College of Pharmaceutical Sciences, Zhejiang University, Hangzhou, P.R. China

*Correspondence to S. W. [email: wangsf@zju.edu.cn]

**Supplementary materials**

**Table S1** Characterization of constituents in GZJSYT by LC-Q-TOF-MS and LC-IT-MS.

**Table S2** The detected constituents (except the 72 constituents in the whole extract) in the fractions of GZJSYT by LC-IT-MS

**Elucidation of the chemical structures of eight potential active constituents by LC-MS and NMR.**

**Table S3** ^1^H-NMR and ^13^C-NMR spectrum of compounds **833** and **836**.

**Table S4** ^1^H-NMR and ^13^C-NMR spectrum of compounds **840** and **890**.

**Table S5** ^1^H-NMR and ^13^C-NMR spectrum of compounds **893**, **838** and **902**.

**Table S6** ^1^H-NMR and ^13^C-NMR spectrum of compounds **883**.

**Figure S1** ^1^H, ^13^C and DEPT 135 NMR spectrum of compound **833**.

**Figure S2** ^1^H, ^13^C and DEPT 135 NMR spectrum of compound **836**.

**Figure S3** ^1^H, ^13^C and DEPT 135 NMR spectrum of compound **838**.

**Figure S4** ^1^H, ^13^C and DEPT 135 NMR spectrum of compound **840**.

**Figure S5** ^1^H, ^13^C and DEPT 135 NMR spectrum of compound **883**.

**Figure S6** ^1^H, ^13^C and DEPT 135 NMR spectrum of compound **890**.

**Figure S7** ^1^H, ^13^C and DEPT 135 NMR spectrum of compound **893**.

**Figure S8** ^1^H, ^13^C and DEPT 135 NMR spectrum of compound **902**.

**Table S1 Characterization of constituents in GZJSYT by LC-Q-TOF-MS and LC-IT-MS**

| No. | t_R_  (min) | Identification | Molecular Formula | Detected *m/z* | Error  (ppm) | ESI-MS^n^  *m/z* (%) | Source | |
| --- | --- | --- | --- | --- | --- | --- | --- | --- |
| 1 | 1.15 | Malic acid | C_4_H_6_O_5_ | 133.0154  [M-H]ˉ | 8.7 | MS^2^[133.20@35]:133.0(2), 115.1(100), 86.8(4) | | GPJZC |
| 2 | 1.25 | Unknown | ----- | ----- | ---- | MS^2^[711.3@35]: 675.0(3), 665.2(100), 650.6(2), 621.5(3), 620.2(3), 612.5(4), 595.3(2), 590.1(3), 549.8(4), 548.5(6), 537.1(2), 489.2(3), 488.4(2), 436.3(3), 388.7(2), 363.6(2), 303.8(3), 273.4(3) | | G |
| 3 | 1.53 | Unknown | ----- | ----- | ---- | MS^2^[290.3@35]: 272.2(15), 254.2(11), 236.2(2), 230.2(14), 214.2(2), 212.2(5), 200.2(100), 170.3(3), 128.3(10) | | PJ |
| 4* | 1.59 | Citric acid | C_6_H_8_O_7_ | 191.0208  [M-H]ˉ | 5.6 | MS^2^[191.3@35]: 173.0(24), 130.9(2), 111.2(100) | | GPJZC |
| 5 | 1.71 | Desbenzoyl paeoniflorin | C_16_H_24_O_10_ | 421.1344 [M+HCOO]ˉ | -1.8 | MS^2^[421.3@35]: 375.0(100), 345.1(77),  MS^3^[421.3@35-->[375.0@40](mailto:375.0@40)]: 345.1(100), 213.0(10), 195.1(47), 177.1(55), 164.9(19),  MS^4^[421.3@35-->375.0@40-->345.1@45]: 327.3(7), 183.2(100), 165.1(38) | | P |
| 6* | 2.07 | Succinic acid | C_6_H_4_O_6_ | ----- | ----- | MS^2^[117.2@35]: 117.2(30), 99.1(41), 73.2(100) | | GPJZC |
| 7 | 2.15 | Isomaltodebenzoyl paeoniflorin | C_22_H_34_O_15_ | 583.1876 [M+HCOO]ˉ | -0.6 | MS^2^[583.3@35]: 537.1(5), 507.1(100), 489.1(18), 374.9(1), 340.9(3)  MS^3^[583.3@35-->507.1@40]: 489.0(100), 340.8(6), 237.2(4), 178.9(3), 165.1(3)  MS^4^[583.3@35-->507.1@40-->489.0@43]: 165.1(100) | | P |
| 8* | 3.26 | Gallic acid | C_7_H_6_O_5_ | 169.0157 [M-H]ˉ | 8.6 | MS^2^[169.3@35]: 169.2(85), 125.3(100) | | P |
| 9 | 3.95 | 1-*O*-*β*-D-Glucopyranosyl paeonisuffrone | C_16_H_24_O_9_ | 405.1404 [M+HCOO]ˉ | 0.4 | MS^2^[405.2@35]: 359.1(100), 197.2(3),  MS^3^[405.3@35-->[359.1@40](mailto:359.1@40)]: 197.0(100), 179.1(85), 161.2(4), 151.0(2) | | P |
| 10 | 4.16 | Glucogallin | C_13_H_16_O_10_ | 331.0669 [M-H]ˉ | -0.5 | MS^2^[331.3@35]: 313.2(3), 271.2(100), 241.1(3), 169.2(2),  MS^3^[331.3@35-->[271.2@40](mailto:271.2@40)]: 211.1(100), 169.2(11) | | P |
| 11 | 5.57 | 6-Galloylsucrose or 6ˊ-Galloylsucrose or 1ˊ-Galloylsucrose | C_19_H_26_O_15_ | 493.1197 [M-H]ˉ | 0.4 | MS^2^[493.4@35]: 331.3(3), 313.3(100), 223.4(3), 169.2(2),  MS^3^[493.4@35-->[313.3@40](mailto:313.3@40)]: 313.2(12), 223.1(49), 205.2(12), 169.1(100), 151.1(22), 125.2(14) | | P |
| 12 | 7.18 | 6-*O*-*β*-D-glucopyranosyl lactinolide | C_16_H_26_O_9_ | 407.1559 [M+HCOO]ˉ | 0 | MS^2^[407.3@35]: 361.2(100),  MS^3^[407.3@35-->[361.2@40](mailto:361.2@40)]: 199.3(10), 180.9(49), 178.6(8), 163.2(100), 161.2(30), 155.0(5), 119.1(18) | | P |
| 13 | 8.46 | 6ˊ-*O*-Galloyl desbenzoyl paeoniflorin | C_23_H_28_O_14_ | 527.1403[M-H]ˉ | 0.6 | MS^2^[527.3@35]: 509.4(4), 497.3(100), 479.3(51), 451.3(2), 271.3(3),  MS^3^[527.4@35-->[497.3@40](mailto:497.3@40)]: 479.2(100), 451.2(3), 271.2(15), 211.0(2), 167.1(1)  MS^4^[527.4@35-->497.3@40-->479.2@45]: 460.8(3), 451.1(12), 399.3(42), 331.4(2), 328.9(4), 313.2(32), 271.1(100), 253.2(10), 211.2(40), 169.2(21), | | P |
| 14 | 8.73 | (*p*-Hydroxybenzyl)malonic acid | C_10_H_10_O_5_ | 209.0465 [M-H]ˉ | 4.6 | MS^2^[209.2@35]: 165.2(100),  MS^3^[209.2@35-->[165.2@40](mailto:165.2@40)]: 165.1(5), 121.2(100), 93.2(25), 59.1(5) | | G |
| 15 | 9.67 | Zizybeoside I or its isomer | C_19_H_28_O_11_ | 477.1613 [M+HCOO]ˉ | -0.1 | MS^2^[477.2@35]: 431.1(100), 413.5(2),  MS^3^[477.2@35-->[431.1@42](mailto:431.1@42)]: 293.1(3), 269.1(100), 178.1(4), 160.9(25) | | J |
| 16 | 9.74 | Unknown | C_16_H_26_O_8_ | 391.1611 [M+HCOO]ˉ | 0.3 | MS^2^[391.3@35]: 345.1(100),  MS^3^[391.3@35-->[345.1@40]: 207.9(26)](mailto:345.1@40%5D: 207.9(26)), 160.9(100) | | P |
| 17 | 10.32 | Zizybeoside I or its isomer | C_19_H_28_O_11_ | 477.1603 [M+HCOO]ˉ | -2.2 | MS^2^[477.2@35]: 431.1(100), 415.9(2),  MS^3^[477.2@35-->[431.1@42](mailto:431.1@42)]: 416.6(14), 311.1(8), 293.0(8), 269.1(100), 161.1(43), 159.1(11), 143.0(6) | | J |
| 18 | 10.32 | Oxypaeoniflorin | C_23_H_28_O_12_ | 541.1560 [M+HCOO]ˉ | -0.5 | MS^2^[541.1@38]: 495.2(100),  MS^3^[541.1@38-->495.5@42]: 477.2(2), 465.2(100), 333.3(8), 327.3(5), 179.1(4), MS^4^[541.1@38-->495.5@42-->465.2@45]: 164.9(100), 137.1(36) | | P |
| 19 | 11.09 | Zizybeoside II | C_25_H_38_O_16_ | 639.2147 [M+HCOO]ˉ | 0.8 | MS^2^[639.3@35]: 593.3(100)  MS^3^[639.3@35-->[593.3@40](mailto:593.3@40)]: 431.1(100), 269.1(5), | | J |
| 20 | 11.81 | Unknown | ----- | ----- | ---- | ---- | | P |
| 21 | 11.85 | 6'-O-*β*-Glucopyranosyl albiflorin or its isomer | C_29_H_38_O_16_ | 687.2152 [M+HCOO]ˉ | 1.5 | MS^2^[687.3@35]: 641.1(100), 519.3(8)  MS^3^[687.3@35-->[641.1@40](mailto:641.1@40)]: 611.2(16), 519.2(100), 488.7(1)  MS^4^[687.4@35-->641.2@40-->519.2@45]: 489.4(100), 471.6(3), 195.0(2) | | P |
| 22 | 12.51 | Unknown | C_35_H_48_O_21_ | 849.2685 [M+HCOO]ˉ | 1.8 | ---- | | P |
| 23 | 12.69 | 6'-O-*β* -Glucopyranosyl albiflorin or its isomer | C_29_H_38_O_16_ | 687.2152 [M+HCOO]ˉ | 1.5 | MS^2^[687.35@35]: 641.2(100),  MS^3^[687.3@35-->641.1@40]: 611.4(2)), 519.1(100), 489.5(2), 470.9(2), 323.1(3)  MS^4^[687.4@35-->641.2@40-->519.2@45]: 489.3(100), 471.2(10), 194.7(12) | | P |
| 24 | 12.95 | Isomaltopaeoniflorin | C_29_H_38_O_16_ | 687.2146 [M+HCOO]ˉ | 0.6 | MS^2^[687.3@35]: 641.0(32), 622.9(10), 611.1(100) , 593.1(44), 489.3(3), 445.3(1), 323.1(2),  MS^3^[687.4@35-->611.1@40]: 593.2(63), 489.0(100), 470.6(10), 444.2(3), 362.4(2), 323.0(10), 305.1(2), 283.1(11)  MS^4^[687.4@35-->611.1@40-->489.0@45]: 237.2(100), 179.0(69), 165.2(53) | | P |
| 25 | 13.19 | Albiflorin | C_23_H_28_O_11_ | 525.1616 [M+HCOO]ˉ | 0.4 | MS^2^[525.2@35]: 479.1(100), 357.4(6), 283.2(4),  MS^3^[525.3@35-->479.1@40]: 357.1(100), 327.0(2), 299.2(2), 283.0(17), 195.2(2)  MS^4^[525.3@35-->479.1@40-->357.1@45]: 327.0(100), 195.2(30) | | P |
| 26 | 13.53 | 6'-O-*β*-Glucopyranosyl albiflorin or its isomer | C_29_H_38_O_16_ | 687.2152 [M+HCOO]ˉ | 1.5 | MS^2^[687.3@35]: 641.1(100), 611.2(11)  MS^3^[687.3@35-->641.1@40]: 611.0(39), 519.1(100), 491.1(3), 489.1(60), 470.9(2), 375.1(12), 327.1(7), 326.3(5)  MS^4^[687.4@35-->641.2@40-->519.2@45]: 489.3(100), 471.2(10), 245.6(2), 213.0(6), 194.7(12) | | P |
| 27* | 14.21 | Paeoniflorin | C_23_H_28_O_11_ | 525.1619 [M+HCOO]ˉ | 1.0 | MS^2^[525.2@35]: 478.9(38), 449.1(100), 431.1(1),  MS^3^[525.3@35-->[449.1@40](mailto:449.1@40)]: 327.0(100), 309.0(4), 165.2(9)  MS^4^[525.3@35-->449.0@40-->327.0@45]: 267.3(2), 165.0(100), | | P |
| 28* | 16.7 | Liquiritigenin-7-*O*-*β*-D- apiofuranosyl-4'-*O*-*β*-D- glucopyranoside | C_26_H_30_O_13_ | 549.1620 [M-H]ˉ | 1.2 | MS^2^[549.5@35]: 429.2(81), 417.2(4), 387.1(2), 297.2(13), 255.3(100),  MS^3^[549.5@35-->255.3@45]: 255.3(4), 214.3(2), 155.2(3), 153.1(35), 135.1(100), 119.3(11), 93.1(2), 91.1(6) | | G |
| 29* | 16.75 | Liquiritin | C_21_H_22_O_9_ | 419.1338 [M+H]^+^ | 0.3 | (ESI+)MS^2^[419.3@35]: 401.1(1), 365.1(1), 299.1(1), 297.8(1), 281.0(1), 257.1(100) | | G |
| 30* | 17.06 | Liquiritin apioside | C_26_H_30_O_13_ | 549.1625 [M-H]ˉ | 2.1 | MS^2^[549.5@35]: 549.5(2), 429.2(13), 417.2(22), 399.2(3), 327.1(2), 297.2(21), 255.3(100)  MS^3^[549.5@35-->255.3@45]: 161.2(3), 153.1(49), 149.2(5), 135.2(100), 120.2(5), 119.3(18), 91.2(3) | | G |
| 31* | 17.09 | Liquiritigenin | C_15_H_12_O_4_ | 257.0812 [M+H]^+^ | 1.4 | (ESI+)MS^2^[257.4@35]: 257.1(70), 240.1(15), 239.1(100), 229.2(2), 211.2(15), 163.2(10), 147.1(95), 137.1(65), 119.2(2) | | G |
| 32 | 17.93 | Galloylalbiflorin | C_30_H_32_O_15_ | 631.1674 [M-H]ˉ | 0.9 | MS^2^[631.4@35]: 613.2(100), 601.2(6), 585.3(3), 583.2(3), 509.2(13), 491.2(34), 479.4(10), 465.3(33), 313.3(8), 271.2(25), 211.3(5),  MS^3^[631.4@35-->613.2@40]: 583.1(2), 503.1(5), 491.3(61), 435.3(3), 431.0(4), 415.2(4), 399.3(10), 375.3(8), 371.2(5), 313.2(22), 311.2(8), 271.1(100), 241.3(8), 239.5(10), 211.2(26) | | P |
| 33 | 18.13 | Apigenin 6-C-*β* -xylopyranosyl-8-C-(6'''- O-(3- hydroxy-3- methylglutaroyl)-*β*- glucopyranoside) | C_32_H_36_O_18_ | 707.1842 [M-H]ˉ | 1.9 | MS^2^[707.5@35]: 645.3(3), 605.3(5), 563.3(12), 545.3(4), 527.3(3), 473.3(26), 443.3(100), 425.4(2) , 383.3(8) , 353.3(6)  MS^3^[707.5@35-->443.3@45]: 425.2(17), 407.2(4), 395.1(5), 383.2(66), 379.1(3), 365.1(5), 353.2(100), 323.1(3) | | G |
| 34 | 18.28 | Unknown | ----- | ----- | ---- | MS^2^[321.0@35]: 305.3(5), 286.2(4), 284.8(32), 276.9(100), 276.1(9), 275.2(18), 264.6(11), 175.2(25), 147.1(12)  MS^3^[321.0@35-->276.8@40]: 264.8(100), 220.9(21) | | ----- |
| 35 | 20.55 | Albiflorin R_1_ | C_23_H_28_O_11_ | 525.1608 [M+HCOO]ˉ | -1.1 | MS^2^[525.2@38]: 479.1(100), 357.2(3), 283.0(3),  MS^3^[525.2@35-->479.1@40]: 460.9(7), 449.2(100), 357.3(3),  MS^4^[525.2@35-->479.1@40-->449.2@45]: 327.4(100) | | P |
| 36* | 21.53 | Glycyroside | C_27_H_30_O_13_ | 607.1684 [M+HCOO]ˉ | 2.6 | MS^2^[607.1@35]: 561.1(100),  MS^3^[607.1@35-->[561.1@40](mailto:561.1@40)]: 267.3(100), 252.5(5) | | G |
| 37* | 22.17 | Ononin | C_22_H_22_O_9_ | 475.1254 [M+HCOO]ˉ | 1.7 | MS^2^[475.3@35]: 428.8(4), 267.3(100),  MS^3^[475.3@35-->[267.3@40](mailto:267.3@40)]: 267.2(100), 252.3(55) | | G |
| 38 | 23.54 | Sacranoside A or its isomer | C_21_H_34_O_10_ | 491.2143 [M+HCOO]ˉ | 1.8 | MS^2^[491.4@35]: 445.2(100), 293.2(5)  MS^3^[491.3@35-->445.2@40]: 294.0(46), 292.8(100), 233.3(8), 190.7(19) | | P |
| 39 | 24.53 | Sacranoside A or its isomer | C_21_H_34_O_10_ | 491.2143 [M+HCOO]ˉ | 1.8 | MS^2^[491.3@35]: 445.2(100) | | P |
| 40 | 26.25 | Macedonoside A isomer | C_42_H_62_O_17_ | 837.3939 [M-H]ˉ | 3.0 | MS^2^[837.6@35]: 819.3(7), 775.6(4), 661.6(7), 643.6(4), 351.2(100)  MS^3^[837.5@35-->351.2@40]: 333.1(10), 289.0(89), 259.3(8), 193.1(100), 174.9(46), 159.2(7), 131.3(34), 113.1(25), 99.0(13) | | G |
| 41 | 26.52 | 24-Hydroxy-licorice-saponin A_3_ | C_48_H_72_O_22_ | 999.4474 [M-H]ˉ | 3.2 | MS^2^[999.7@35]: 981.5, 879.1(5), 837.4(100), 819.5(9), 761.1(9), 351.1(23)  MS^3^[999.7@35-->837.4@40]: 793.7(4), 775.2(5), 731.6(5), 706.7(4), 644.9(7), 351.2(100), 332.6(5) | | G |
| 42 | 27.08 | 24-Hydroxy-licorice-saponin A_3_ isomer | C_48_H_72_O_22_ | 999.4489 [M-H]ˉ | 4.7 | MS^2^[999.7@35]: 981.5(5), 955.5(3), 864.7(4), 837.4(100) , 819.4(36), 661.2(6), 643.7(2) , 351.2(23)  MS^3^[999.7@35-->837.4@40]: 864.6(5), 775.4(22), 713.3(5), 661.7(6), 351.1(100) | | G |
| 43 | 27.47 | 22-Hydroxy-licorice-saponin G_2_ | C_48_H_72_O_21_ | 853.3895 [M-H]ˉ | 3.2 | MS^2^[853.66@35]: 677.5(34), 659.4(13), 501.4(20), 351.2(100)  MS^3^[853.7@35-->351.2@40]: 289.2(24), 193.1(97), 174.7(36), 173.8(40), 131.1(100), 113.2(42) | | G |
| 44 | 27.99 | Benzoylpaeoniflorin | C_30_H_32_O_12_ | 629.1891 [M+HCOO]ˉ | 2.4 | MS^2^[629.3@35]: 583.0(92), 553.0(100), 534.8(2), 431.0(2),  MS^3^[629.3@35-->[553.0@40](mailto:553.0@40)]: 430.9(100), 403.3(4), 265.2(3), 247.2(2), 245.4(4), 165.1(7) | | P |
| 45* | 28.4 | Licorice saponin A_3_ | C_48_H_72_O_21_ | 983.4535 [M-H]ˉ | 4.2 | MS^2^[983.71@35]: 939.0(33), 821.5(100), 803.4(69), 351.2(26)  MS^3^[983.2@35-->[821.5@40](mailto:821.5@40)]: 803.5(8), 759.2(6), 741.3(4), 715.3(3), 645.5(12), 351.1(100), 288.9(3) | | G |
| 46 | 28.62 | Albiflorin R_3_ | C_30_H_32_O_12_ | 629.189 [M+HCOO]ˉ | 2.3 | MS^2^[629.4@35]: 583.1(100), 553.1(2), 461.3(2), 387.0(4)  MS^3^[629.4@35-->583.1@40]: 553.3(13), 537.5(6), 461.0(100), 386.9(29) | | P |
| 47 | 29.53 | Yunganoside K_1_ or its isomer | C_48_H_72_O_21_ | 983.4535 [M-H]ˉ | 3.2 | MS^2^[983.6@35]: 965.5(25), 921.5(7), 821.5 (44), 661.7(8), 599.3(5), 497.2(100), 434.9(7), 380.9(5), 339.2(24) | | G |
| 48* | 29.84 | Macedonoside A | C_42_H_62_O_17_ | 837.3951 [M-H]ˉ | 4.4 | MS^2^[837.6@35]: 819.5(13), 775.5(17), 703.5(2), 661.5(34), 643.5(15), 485.5(12), 351.2(100), 333.1(2) | | G |
| 49 | 30.16 | Uralsaponin R /S | C_48_H_74_O_20_ | 969.4723 [M-H]ˉ | 2.3 | MS^2^[969.7@35]: 951.5(18), 925.6(3), 907.6(37), 823.6(4), 643.5(28), 553.5(24), 509.3(6), 485.5(100), 441.4(10), 423.5(5), 355.6(11),  MS^3^[969.1@35-->[485.5@40](mailto:485.5@40)]: 441.4(84), 423.5(22), 355.5(100),  MS^4^[969.73@35-->[485.50@40-->355.50@45](mailto:485.50@40-->355.50@45)]: 355.53(100), 340.27(16), 339.34(40), 199.46(17) | | G |
| 50 | 30.60 | Yunganoside K_2_ or its isomer | C_42_H_62_O_17_ | 837.3950 [M-H]ˉ | 4.3 | MS^2^[837.6@35]: 819.3(16), 775.6(10), 757.7 (2) 661.4 (13), 643.5(9), 485.5(7), 351.3 (100), 307.4(2), 289.3(3), 261.3(2) | | G |
| 51 | 30.66 | Uralsaponin P | C_42_H_64_O_16_ | 823.4147 [M-H]ˉ | 3.1 | MS^2^[823.7@35]: 805.4(4), 779.5(5), 761.5(10), 643.4(36), 599.6(3), 485.5(100), 441.3(7), 423.5(2), 355.5(8)  MS^3^[823.7@35-->485.5@40]: 441.8(21), 423.3(7), 355.4(100) | | G |
| 52 | 31.4 | Unknown | C_16_H_32_O_5_ | 303.2181 [M-H]ˉ | 1.3 | MS^2^[303.5@35]: 303.3(100), 292.4(12), 284.8(11), 258.7(16) | | J |
| 53* | 31.99 | Licorice saponin G_2_ | C_42_H_62_O_17_ | 837.3950 [M-H]ˉ | 4.3 | MS^2^[837.6@35]: 819.3(9), 793.5(5), 775.8 (3), 661.5 (13), 351.2 (100), 333.1(3) | | G |
| 54 | 32.77 | Yunganoside L_1_/J_1_ | C_48_H_72_O_20_ | 967.4576 [M-H]ˉ | 3.3 | MS^2^[967.7@35]: 949.5(8), 905.7(7), 497.3 (100), 435.3(4), 409.2(2), 321.2(9) | | G |
| 55 | 32.96 | Yunganoside K_2_ or its isomer | C_42_H_62_O_17_ | 837.3954 [M-H]ˉ | 4.7 | MS^2^[837.7@35]: 819.3(11), 793.5(5), 775.6 (6), 661.6(4), 643.5(3), 351.2(100) | | G |
| 56 | 33.22 | Yunganoside G_1_ | C_48_H_74_O_21_ | 985.4688 [M-H]ˉ | 3.9 | MS^2^[985.6@35]: 967.5(63), 923.2(23), 905.5 (27), 663.7(63), 497.0(100)  MS^3^[985.8@35-->497.4@40]: 453.0(5), 435.2(21), 417.1(28), 339.0(100), 321.1(62), 155.3(5) | | G |
| 57 | 33.5 | Yunganoside K_2_ or its isomer | C_42_H_62_O_17_ | 837.3954 [M-H]ˉ | 4.5 | MS^2^[837.7@35]: 819.6(4), 775.5(7), 713.3 (2), 661.6(21), 351.2 (100), 333.2(2) | | G |
| 58 | 33.79 | Yunganoside G_2_ or its isomer | C_42_H_64_O_17_ | 839.4105 [M-H]ˉ | 4.1 | MS^2^[839.6@35]: 821.5(14), 95.6(3), 777.5 (6), 663.5(18), 351.2(100) | | G |
| 59* | 34.03 | Uralsaponin A | C_42_H_62_O_16_ | 821.4012 [M-H]ˉ | 5.7 | MS^2^[821.7@35]:803.6(6), 777.3(2), 759.6 (11), 645.5(8), 469.7(2), 351.2(100)  MS^3^[821.8@35-->351.2@40]: 289.0(49), 193.2(100), 174.6(38), 130.8(13), 113.1(30) | | G |
| 60 | 34.04 | Unknown | C_30_H_44_O_3_ | 453.3362 [M+H]^+^ | 0.3 | (ESI+)MS^2^[453.9@35]: 453.3(7), 435.3(81), 407.3(100), 389.3(23), 343.2(8), 331.2(6), 299.2(7), 285.3(11), 267.3(6), 245.2(7), 241.1(17), 227.2(6), 217.1(6), 189.1(11) | | J |
| 61 | 35.13 | Yunganoside L_1_/J_1_ | C_48_H_72_O_20_ | 967.4590 [M-H]ˉ | 4.7 | MS^2^[967.7@35]: 949.5(12), 923.5(2), 905.5 (4),584.5(2), 538.8(2), 645.4(2), 497.3(100), 435.3(6), 339.2(18), 321.3(10), | | G |
| 62 | 35.37 | Albiziasaponin B | C_48_H_74_O_20_ | 969.4744 [M-H]ˉ | 4.5 | MS^2^[969.7@35]: 952.5(5), 951.4(13), 926.8(2), 908.7(6), 907.7(4), 835.7(2), 647.5(7), 555.2(2), 498.3(12), 497.3(100) 471.5(3), 453.5(3), 435.3(2), 407.3(4), 381.1(2), 339.2(14), 321.5(3) | | G |
| 63* | 35.7 | Licorice saponin H_2_ | C_42_H_62_O_16_ | 821.4009 [M-H]ˉ | 5.3 | MS^2^[821.7@35]: 803.5(7), 759.6(8), 645.6 (9), 627.5(3), 469.5(3), 351.2(100), 333.1(2), 289.2(4)  MS^3^[821.8@35-->351.2@40]: 333.6(16), 289.2(33), 193.1(100), 175.1(25), 130.8(24), 113.1(57) | | G |
| 64 | 35.7 | Unknown | C_30_H_44_O_3_ | 453.3364 [M+H]^+^ | 0.2 | (ESI+)MS^2^[453.6@35]: 435.3(100), 417.2(6), 407.3(66), 389.3(33), 311.2(6), 285.1(8), 273.2(7), 267.2(9), 257.1(8), 245.1(9), 241.1(10), 235.1(36), 231.1(7), 217.1(24), 203.1(6), 189.1(23), 175.2(12) | | J |
| 65 | 36.27 | Glycyrrhizic acid isomer | C_42_H_62_O_16_ | 821.4009 [M-H]ˉ | 5.3 | MS^2^[821.7@35]:803.6(11), 777.6(6), 759.6 (20), 741.6(2), 645.4(2), 627.5(20), 351.2(100), 289.2(4)  MS^3^[821.8@35-->351.2@40]: 289.3(34), 200.9(30), 193.1(100), 113.1(80) | | G |
| 66 | 38.09 | Licorice saponin C_2_ or its isomer | C_42_H_62_O_15_ | 805.4056 [M-H]ˉ | 5.0 | MS^2^[805.7@35]: 787.4(7), 743.6(4), 629.5 (4), 351.2(100), 333.1(2) | | G |
| 67 | 38.59 | Actinopolysporin B | C_16_H_30_O_4_ | 285.2077 [M-H]ˉ | --- | MS^2^[285.5@35]: 285.3(100), 267.4(41), 249.4(5), 187.3(12), 157.3(19), 141.3(4) | | --- |
| 68 | 39.21 | Unknown | ----- | ----- | ---- |  | | C |
| 69 | 39.48 | Unknown | ----- | ----- | ---- |  | | C |
| 70 | 39.66 | Unknown | ----- | ----- | ---- |  | | --- |
| 71 | 39.82 | Unknown | ----- | ----- | ---- |  | | C |
| 72 | 40.06 | Unknown | ----- | ----- | ---- |  | | P |

t_R_: retention time; G: *Glycyrrhizae radix et rhizoma preparata cum melle*; P: *Paeoniae radix alba* ; J: *Jujubae fructus*; C: *Cinnamomi ramulus*; Z: *Zingiberis rhizoma recens*.

* Compared with reference standards

**Table S2** The detected constituents (except the 72 constituents in the whole extract) in the fractions of GZJSYT by LC-IT-MS

| **No.** | **t_R_(min)** | | **[M-H]^-^** | **MS^2^** | **No.** | **t_R_(min)** | **[M-H]^-^** | **MS^2^** |
| --- | --- | --- | --- | --- | --- | --- | --- | --- |
| 73 | 1.16 | 195.0 | | MS2[195.0@35]: 177.1(100), 97.2(76) | 82 | 2.31 | 358.1 | MS2[358.0@35]: 311.9(100), 310.9(3), 134.3(26) |
| 74 | 1.52 | 481.2 | | MS2[481.2@35]: 421.2(2), 301.2(100), 275.2(25) | 83 | 2.54 | 312.1 | MS2[312.1@35]: 265.9(100), 265.0(5), 134.3(51) |
| 75 | 1.63 | 583.2^a^ | | MS2[583.1@35]: 536.8(3), 507.0(100), 489.2(14), 341.1(2) | 84 | 2.59 | 384.2 | MS2[384.2@35]: 420.3(1), 384.0(3), 367.1(3), 366.1(100), 340.2(45), 322.1(3), 314.0(3), 308.1(4), 252.1(9), 234.1(8), 208.2(82) |
| 76 | 1.65 | 379.1 | | MS2[379.1@35]: 308.8(1), 281.1(100), 280.4(2), 233.5(1), 150.2(1) | 85 | 2.83 | 331.2 | MS2[331.1@35]: 271.1(100), 169.1(3) |
| 77 | 1.84 | 331.2 | | MS2[331.2@35]: 313.1(3), 295.1(3), 241.1(100), 240.4(2), 223.2(2), 169.1(36) | 86 | 2.89 | 347.2 | MS2[347.2@35]: 348.2(12), 347.2(100), 346.3(24), 303.4(5), 302.2(1) |
| 78 | 1.97 | 421.1 ^a^ | | MS2[421.1@35]: 438.4(2), 387.1(5), 375.0(100), 194.9(4) | 87 | 2.97 | 421.2 ^a^ | MS2[421.2@35]: 375.1(100), 213.1(3), 194.6(3) |
| 79 | 1.98 | 493.3 | | MS2[493.3@35]: 493.1(5), 331.1(51), 313.2(100), 295.2(1), 271.8(1), 271.2(5), 265.0(1), 253.2(4), 233.4(1), 223.3(1), 221.0(1), 211.3(2), 193.1(2), 169.2(22), 143.0(1) | 88 | 3.18 | 527.3 | MS2[527.2@35]: 528.2(3), 527.2(10), 497.1(88), 418.2(2), 365.3(2), 313.2(100), 217.1(2), 211.1(2), 169.1(13) |
| 80 | 2.02 | 481.2 | | MS2[481.2@35]: 302.2(2), 301.2(100), 275.2(9) | 89 | 3.29 | 657 | MS2[567.2@35]: 521.1(100), 520.4(1), 323.0(1) |
| 81 | 2.02 | 493.3 | | MS2[493.4@35]: 475.3(2), 331.2(54), 314.2(5), 313.2(100), 271.1(9), 269.4(4), 223.4(3), 193.3(6), 169.2(29), 163.0(3) | 90 | 3.29 | 405.2 ^a^ | MS2[405.2@35]: 359.0(75), 358.0(3), 341.0(2), 197.0(100), 179.0(6), 161.1(17), 153.1(7), 143.0(1), 135.2(7), 124.0(1) |
| 91 | 3.53 | 567.2 ^a^ | | MS2[567.2@35]: 521.1(100), 520.4(1), 323.0(1) | 98 | 4.16 | 407.1 | MS2[407.1@35]: 407.1(1), 381.8(1), 369.4(1), 361.1(100), 360.2(1),  346.0(1), 345.3(1), 180.3(1), 178.9(15), 160.8(1) |
| 92 | 3.69 | 650.2 | | MS2[650.2@35]: 632.2(2), 611.9(2),  607.2(2), 606.6(2), 538.0(100), 537.3(2), 519.4(1), 499.0(41), 489.8(10), 471.1(2), 424.2(13), 405.4(3), 386.9(8), 385.1(4), 369.0(3), 344.0(15), 326.0(12), 323.0(5), 305.1(2), 273.2(12), 246.2(4), 211.3(4) | 99 | 4.38 | 403.2 | MS2[403.1@35]: 357.0(100), 356.1(2), 267.1(8), 178.9(6) |
| 93 | 4.00 | 673.3 | | MS2[673.3@35]: 673.3(1), 655.2(1), 630.0(4), 583.3(1), 541.1(11), 538.0(100), 522.0(60), 520.0(11), 490.0(11), 425.8(4), 424.0(16), 408.2(2), 386.7(5), 369.0(2), 346.1(2), 344.1(24), 328.1(3), 326.0(7), 211.0(5) | 100 | 4.43 | 493.3 | MS2[493.3@35]: 403.2(5), 331.1(34), 313.2(100), 312.6(3), 283.2(25), 271.2(2), 241.1(26), 223.0(2), 211.1(4), 169.2(14) |
| 94 | 3.95 | 405.3 ^a^ | | MS2[405.1@35]: 359.1(100), 358.5(8), 197.1(1) | 101 | 4.59 | 475.3 | MS2[475.3@35]: 457.0(11), 415.1(100), 283.0(2), 252.8(3), 163.1(8) |
| 95 | 3.86 | 729.2 ^a^ | | MS2[729.2@35]: 669.0(100), 487.0(60), 431.0(80), 299.2(94) | 101 | 4.59 | 475.3 | MS2[475.3@35]: 457.0(11), 415.1(100), 283.0(2), 252.8(3), 163.1(8) |
| 96 | 4.06 | 384.3 | | MS2[384.3@35]: 366.2(100), 340.2(73), 283.0(2), 252.2(5), 234.2(25), 209.2(2), 208.2(86), 207.6(3), 190.2(3), 133.2(2) | 102 | 4.84 | 463.2 | MS2[463.2@35]: 445.1(8), 433.2(5), 427.0(7), 403.1(100), 373.1(100), 343.2(29), 301.2(41) |
| 97 | 4.12 | 891 ^a^ | | MS2[729.2@35]: 669.0(100), 487.0(60), 431.0(80), 299.2(94) | 103 | 4.89 | 1017.3 | ---- |
| 104 | 4.87 | 493.3 | | MS2[493.3@35]: 425.4(2), 331.0(40), 313.2(6), 271.2(100), 270.4(2),  211.2(3) | 111 | 5.58 | 465 | ---- |
| 105 | 4.71 | 655.3 | | MS2[655.3@35]: 595.2(1), 535.2(1), 493.7(1), 493.1(63), 475.2(100), 474.3(1), 403.3(9), 373.5(1), 331.1(5), 313.1(25), 295.1(11), 283.2(1), 271.0(1), 265.1(1), 241.1(38), 223.2(6), 219.0(1), 211.1(4), 205.1(2) | 112 | 5.71 | 919.4 | MS2[919.3@35]: 873.7(2), 859.8(3), 716.4(100) |
| 106 | 5.06 | 345 | | MS2[345.0@35]: 316.1(2), 299.0(100), 137.3(4) | 113 | 5.72 | 483.2 | MS2[483.3@35]: 331.1(100), 313.2(20), 295.1(2), 271.1(2), 193.2(2), 169.2(26) |
| 107 | 5.22 | 653.5 | | ---- | 114 | 5.73 | 527.3 | MS2[527.3@35]: 528.4(3), 527.2(46), 498.4(4), 497.2(100), 479.2(8), 313.2(66), 283.2(2), 270.8(2), 169.2(10) |
| 108 | 5.26 | 389.2 | | MS2[389.2@35]: 342.9(100), 313.0(3), 181.1(21), 163.2(3), 161.0(31), 151.1(9), 142.8(2) | 115 | 5.91 | 817.4 | MS2[817.4@35]: 799.4(13), 787.3(8), 702.3(100), 701.7(2), 684.3(5), 672.4(9), 671.3(17), 653.3(2), 598.2(2), 290.4(2) |
| 109 | 5.41 | 331.3 | | MS2[331.3@35]: 331.2(100), 313.1(29), 287.2(3), 211.1(5), 169.3(11), 168.2(27), 167.2(6), 125.1(3), 123.4(2) |  |  |  |  |
| 110 | 5.62 | 153.2 | | MS2[153.1@35]: 153.1(100), 110.3(4), 109.2(82) | 116 | 5.37 | 655.3 | MS2[655.3@35]: 595.2(8), 577.3(2), 535.0(4), 493.1(62), 475.2(24), 457.1(2), 433.1(61), 432.3(5), 415.0(16), 403.1(32), 373.2(43), 372.4(2), 331.1(100),  271.1(45) |
| 117 | 5.41 | 817.3 | | MS2[817.3@35]: 757.1(14), 739.2(16), 697.1(5), 655.1(100), 637.4(6), 595.1(20), 594.3(2), 577.1(15), 535.3(3), 493.2(76), 433.1(16), 415.1(3), 331.0(30), 271.1(9) | 125 | 6.51 | 520.1 ^a^ | MS2[520.1@35]: 474.1(100), 471.6(1) |
| 118 | 5.64 | 225.1 | | MS2[225.1@35]: 180.8(56), 180.1(10), 179.5(5), 178.6(3), 109.2(100),  103.1(2) | 126 | 6.75 | 520.1 ^a^ | MS2[520.1@35]: 474.1(100) |
| 119 | 6.06 | 904.5 | | MS2[904.5@35]: 886.2(14), 874.3(10), 856.3(4), 790.4(6), 789.4(100), 788.7(3), 771.4(7), 759.3(17), 741.4(2), 740.7(3), 728.4(6) | 127 | 6.78 | 266.3 ^b^ | (ESI+)MS2[266.3@35]: 267.2(1), 266.1(24), 249.1(1), 248.1(100), 238.2(3), 236.1(9), 220.1(42), 202.1(6), 196.1(2), 195.4(1), 80.1(2) |
| 120 | 6.02 | 688.2 ^a^ | | MS2[688.2@35]: 658.3(19), 634.4(31), 627.8(14), 626.0(10), 625.1(11), 612.0(100) | 128 | 7.05 | 625.3 | MS2[625.3@35]: 625.3(38), 605.1(1), 582.1(3), 565.2(1), 562.9(1), 490.1(100), 487.2(1), 474.0(1), 472.0(10), 463.1(2), 460.5(1), 362.1(31), 360.0(1), 277.4(1), 225.0(2), 211.1(9), 207.1(2) |
| 121 | 6.22 | 526.1 | | MS2[526.1@35]: 479.8(60), 450.0(100), 449.4(2) | 129 | 7.10 | 479 ^a^ | MS2[479.0@35]: 442.1(16), 432.1(12), 419.2(13), 418.1(25), 415.5(10), 347.1(100), 316.1(9), 293.2(15), 285.1(45), 275.0(26), 190.2(8), 160.3(23) |
| 122 | 6.33 | 493.1 | | MS2[493.2@35]: 446.8(100), 293.0(99), 149.1(3) | 130 | 7.33 | 543.1 ^a^ | MS2[543.1@35]: 507.2(13), 497.2(100), 363.9(18) |
| 123 | 6.47 | 463.3 | | ---- | 131 | 7.60 | 497.3 | ---- |
| 124 | 6.71 | 387.1 ^a^ | | MS2[387.1@35]: 341.0(100), 179.1(8) | 132 | 7.27 | 527.2 | MS2[527.2@35]: 509.1(2), 499.7(1), 497.2(100), 479.2(48), 331.2(1), 271.1(2) |
| 133 | 7.35 | 475.2 | | MS2[475.2@35]: 431.1(100), 429.7(1), 323.1(1), 269.0(23), 268.2(1), 263.3(1), 221.0(1), 178.9(1), 161.0(9),  142.8(1) | 141 | 8.26 | 757.4 | MS2[757.4@35]: 739.2(5), 697.3(3), 679.2(2), 667.2(38), 637.3(100), 595.2(3), 547.4(2), 517.3(7), 475.2(4) |
| 134 | 7.38 | 483.2 | | MS2[483.2@35]: 465.0(10), 447.2(5), 423.2(3), 331.1(16), 313.1(28), 271.1(100), 235.2(3), 211.1(40), 193.2(73), 169.2(4), 168.2(2), 165.0(2) | 142 | 8.38 | 451.2 | MS2[451.2@35]: 289.1(100) |
| 135 | 7.49 | 511.2 | | MS2[511.2@35]: 511.1(15), 493.1(10), 475.2(13), 451.2(6), 433.2(3), 422.2(6), 421.2(100), 391.2(28) | 143 | 8.99 | 553.2 ^a^ | MS2[553.2@35]: 507.1(100), 354.9(1), 341.0(1), 281.0(1), 178.6(1) |
| 136 | 7.66 | 633.2 | | MS2[633.2@35]: 481.2(46), 463.1(22), 301.3(100), 300.4(3), 275.3(7), 273.3(2) | 144 | 8.21 | 593.3 | MS2[593.2@35]: 593.2(75), 503.4(2), 473.2(100), 431.3(36), 430.4(11), 311.2(11), 310.3(5) |
| 137 | 7.81 | 609.2 | | MS2[609.2@35]: 591.1(3), 565.0(20), 547.0(7), 489.4(2), 482.7(6), 466.9(3), 457.0(41), 455.0(10), 447.1(30), 439.1(100), 413.3(3), 395.1(4), 365.4(2), 329.1(3), 287.0(2), 276.9(12), 259.5(2), 196.6(2) | 145 | 8.14 | 877.1 | MS2[877.1@35]: 876.9(33), 859.4(51), 841.2(80), 835.1(63), 834.1(54), 797.0(47), 790.1(23), 789.4(100), 721.3(27), 688.4(53), 338.0(66) |
| 138 | 7.52 | 389.1 | | MS2[389.1@35]: 343.0(100), 181.1(2) | 146 | 8.23 | 463.1 | MS2[463.1@35]: 416.9(100), 293.0(23) |
| 139 | 7.84 | 446.2 | | MS2[446.2@35]: 284.2(100), 161.1(3) | 147 | 8.36 | 715.1 | ---- |
| 140 | 7.96 | 483.2 | | MS2[483.2@35]: 423.1(100), 405.2(2), 331.2(5), 313.1(3), 271.2(9), 211.2(3), 193.2(2), 169.2(3) | 148 | 8.52 | 539.2 ^a^ | MS2[539.2@35]: 493.1(100), 492.5(2), 306.9(2), 163.0(2) |
| 149 | 8.63 | 461.4 | | MS2[461.4@35]: 461.2(1), 366.8(1), 315.2(100), 314.2(1), 205.1(1), 179.2(4), 143.2(2), 135.3(31), 134.1(3) | 155 | 8.85 | 483.3 | MS2[483.3@35]: 423.1(100), 405.3(3), 331.2(6), 313.0(2), 271.1(11), 211.2(2), 193.2(4), 169.2(3) |
| 150 | 8.68 | 391.2 | | MS2[391.1@35]: 326.8(100), 314.9(5), 179.0(17), 165.3(2), 161.0(25), 159.4(2), 143.2(2) | 156 | 8.83 | 635.2 | MS2[635.2@35]: 483.1(100), 465.2(17), 331.2(4), 320.9(3) |
| 151 | 8.70 | 443.7 | | ---- | 157 | 8.49 | 816 | MS2[816.0@35]: 798.2(100), 797.4(36), 786.3(63), 654.1(69), 597.3(81) |
| 152 | 8.76 | 475.3 | | MS2[475.2@35]: 431.1(100), 323.0(2), 321.8(1), 269.0(39), 263.2(2), 245.2(1), 232.8(1), 178.9(3), 161.0(12), 159.1(1) | 158 | 8.78 | 577.1 | MS2[577.1@35]: 559.2(5), 451.1(31), 425.1(100), 424.2(3), 407.3(44), 406.6(4), 289.1(17), 287.2(2), 273.1(3), 245.2(4) |
| 153 | 8.83 | 643.3 | | MS2[643.3@35]: 643.2(20), 625.3(4), 607.0(4), 511.1(3), 475.2(2), 451.2(3), 434.5(2), 433.1(2), 421.2(74), 391.2(100), 349.2(5), 282.3(2) | 159 | 8.90 | 527.4 | MS2[527.3@35]: 527.2(12), 509.2(33), 497.2(100), 491.2(65), 483.3(8), 479.1(10), 465.3(5), 447.3(17), 399.4(3), 375.3(27), 346.2(3), 345.3(12), 339.1(8), 313.2(73), 271.2(32), 211.3(8), 177.1(6), 169.2(10) |
| 154 | 8.83 | 527.3 | | MS2[527.2@35]: 527.2(8), 509.2(41), 498.2(2), 497.2(100), 491.2(88), 483.4(6), 479.3(10), 465.1(6), 447.3(30), 436.2(5), 435.2(5), 405.1(2), 375.3(40), 357.3(3), 345.3(16), , 313.2(94), 312.5(3), 295.2(2), 285.3(2), 271.0(46), 169.2(23), 168.0(2) | 160 | 8.91 | 728.2 | MS2[728.2@35]: 728.5(2), 710.1(4), 655.8(4), 630.1(5), 593.3(3), 461.1(70), 443.1(6), 408.1(22), 399.1(20), 381.1(100), 357.1(4), 346.1(39), 339.3(33), 273.2(9), 260.9(3) |
| 161 | 9.02 | 511.2 | | MS2[511.2@35]: 493.1(69), 475.0(4), 457.0(2), 433.1(2), 431.1(7), 421.1(16), | 162 | 9.12 | 540.8 | ---- |
|  |  |  | | 413.1(6), 391.1(100), 341.2(7), 311.2(15) |  |  |  |  |
| 163 | 9.17 | 709.3 | | MS2[709.3@35]: 709.1(1), 589.3(1), 487.2(5), 457.2(100), 456.6(2), 439.3(1), 429.2(3), 367.5(1), 337.0(1), 266.2(1), 249.2(1) | 172 | 9.59 | 709.3 | MS2[709.4@35]: 709.3(2), 547.2(1), 487.2(3), 457.2(100), 456.3(1), 439.3(1), 429.3(1) |
| 164 | 9.13 | 607.2 | | MS2[607.2@35]: 607.2(84), 589.2(3), 487.2(100), 485.3(3), 445.2(2), 325.3(5) | 173 | 9.97 | 431.3 | MS2[431.3@35]: 431.2(22), 311.2(100), 310.5(2), 283.2(3) |
| 165 | 9.26 | 577.1 | | MS2[577.1@35]: 559.1(5), 451.2(25), 450.4(3), 425.0(100), 407.2(46), 381.1(2), 299.1(3), 289.1(13) | 174 | 9.50 | 483.2 | MS2[483.2@35]: 483.2(9), 465.1(2), 439.3(4), 423.1(4), 331.1(25), 313.2(14), 271.1(100), 211.2(13), 193.2(2),  169.3(4) |
| 166 | 9.20 | 553.4 | | MS2[553.0@35]: 535.1(3), 507.1(100), 477.3(2), 468.7(2), 400.2(2), 236.9(2) | 175 | 9.60 | 167.2 | MS2[167.1@35]: 167.1(85), 152.1(100) |
| 167 | 9.26 | 407.1 | | MS2[407.1@35]: 397.6(8), 389.1(24), 361.1(100), 156.8(8) | 176 | 9.71 | 623.1 | MS2[520.1@35]: 474.1(100), 471.6(1) |
| 168 | 9.22 | 401.0 | | MS2[401.0@35]: 391.9(2), 383.4(2), 382.6(2), 355.0(100), 351.5(3), 193.2(18), 152.0(2) | 177 | 10.36 | 784.2 | MS2[623.1@35]: 576.6(11), 575.3(5), 415.3(100) |
| 169 | 9.29 | 404.1 | | MS2[404.1@35]: 357.8(100), 312.2(16) | 178 | 9.34 | 327.1 | MS2[327.2@35]: 305.9(1), 281.6(1), 205.1(100), 169.0(1), 160.9(1), 143.1(2), 125.1(3) |
| 170 | 9.30 | 327.1 | | MS2[327.1@35]: 205.0(100), 204.0(2), 169.0(1), 161.0(1), 143.0(2), 142.0(1), 125.0(3) | 179 | 9.64 | 349.3 | MS2[520.1@35]: 474.1(100), 471.6(1) |
| 171 | 9.40 | 391.1 | | MS2[391.1@35]: 345.1(100), 344.1(1), 179.0(1) | 180 | 9.74 | 334.9 ^a^ | MS2[334.9@35]: 289.1(100) |
| 181 | 9.86 | 540.9 | | MS2[540.8@35]: 409.2(37), 379.1(100), 160.9(50) | 188 | 10.55 | 664.2 | MS2[664.1@35]: 574.5(29), 549.1(100) |
| 182 | 9.93 | 611.2 | | MS2[611.3@35]: 593.1(10), 575.1(3), 557.2(1), 521.2(15), 520.6(2), 503.1(5), 491.1(100), 490.3(2), 485.3(1), 473.1(7), 467.2(1), 455.1(1), 443.2(3), 437.2(1), 431.2(9), 413.0(5), 401.2(36), 385.4(1), 371.2(67), 370.4(1), 305.1(1), 278.8(1), 235.1(1), 188.9(1) | 189 | 10.25 | 431.0 | MS2[431.0@35]: 431.1(13), 413.1(6), 386.9(8), 385.5(2), 341.4(4), 324.3(2), 311.2(100), 310.3(7), 264.0(7) |
| 183 | 10.17 | 635.2 | | MS2[635.2@35]: 635.1(2), 576.6(2), 483.1(27), 465.0(100), 423.1(6), 421.2(2), 313.2(8), 295.2(6), 235.1(2) | 190 | 10.24 | 653.1 | MS2[653.1@35]: 533.2(3), 445.2(100) |
| 184 | 10.33 | 643.2 | | ---- | 191 | 10.35 | 449.2 | MS2[449.2@35]: 421.1(2), 287.1(100), 286.2(3), 269.2(7), 259.3(10) |
| 185 | 10.40 | 664.1 | | MS2[664.0@35]: 735.4(32), 619.2(100), 518.1(36), 501.3(27) | 192 | 10.59 | 594.3 | MS2[594.3@35]: 577.3(100), 462.0(2) |
| 186 | 10.46 | 635.3 | | MS2[635.2@35]: 635.2(3), 616.9(2), 483.2(100), 482.4(2), 466.2(2), 465.1(59), 423.2(21), 405.2(6), 313.1(12), 295.1(14), 271.3(3), 235.3(2), 233.1(2), 211.2(5), 205.1(2), 193.2(4) | 193 | 10.49 | 639.1 | MS2[639.1@35]: 587.1(100) |
| 187 | 10.53 | 563.2 | | MS2[563.2@35]: 341.2(4), 312.3(1), 311.2(100), 309.5(1), 293.2(1),  283.3(12) | 194 | 10.62 | 539.3 | MS2[539.3@35]: 963.7(2), 595.2(3), 493.1(100), 306.9(5) |
| 195 | 10.62 | 737.4 | | MS2[737.4@35]: 707.4(2), 615.2(100), 471.3(2), 309.2(5), 291.1(2) | 204 | 11.15 | 687.2 ^a^ | MS2[687.2@35]:641.1(100), 626.6(2), 598.8(4), 596.8(7), 525.0(7), 519.0(4), 492.1(8), 476.0(6), 454.9(10) |
| 196 | 10.67 | 329.0 | | MS2[329.0@35]: 282.8(100), 281.8(7), 210.3(2), 192.9(6), 160.8(13), 143.2(2), 121.1(5), 119.2(4) | 205 | 11.32 | 631.2 | MS2[631.2@35]: 594.4(1), 549.2(100), 544.3(1), 255.2(1) |
| 197 | 10.77 | 725.3 | | MS2[725.3@35]: 724.9(1), 563.3(1), 539.2(1), 503.4(2), 473.2(100), 311.1(2) | 206 | 11.33 | 575.3 | ---- |
| 198 | 10.88 | 437.2 | | MS2[437.2@35]: 408.6(1), 292.5(32), 290.5(2), 286.2(1), 275.0(100), 271.2(1), 231.2(82), 229.2(1), 189.3(2), 148.8(3) | 207 | 11.36 | 433.9 | MS2[434.0@35]: 434.3(100), 387.4(48) |
| 199 | 10.96 | 563.2 | | MS2[563.2@35]: 341.1(3), 312.2(5), 311.2(100), 283.3(13), 227.3(2) | 208 | 11.58 | 502.0 | MS2[502.0@35]: 456.0(100), 455.4(3), 322.8(1) |
| 200 | 11.03 | 492.2 | | MS2[492.2@35]: 473.9(2), 464.2(1), 446.0(100), 445.1(1), 285.1(1), 284.1(7) | 209 | 11.72 | 502.1 | MS2[502.0@35]: 456.0(100), 454.7(1), 323.1(1) |
| 201 | 11.05 | 965.3 | | ---- | 210 | 11.79 | 415.3 | MS2[415.3@35]: 415.2(19), 325.3(2), 296.1(11), 295.2(100), 267.4(3) |
| 202 | 11.09 | 883.2 | | MS2[883.2@35]: 901.7(43), 864.9(31), 836.9(36), 807.4(22), 789.0(17), 788.2(44), 677.2(100) | 211 | 11.34 | 595.3 | MS2[595.3@35]: 577.2(16), 505.1(35), 487.1(21), 475.1(100), 457.2(18), 415.2(25), 397.2(5), 385.2(79), 355.2(78), 343.3(2) |
| 203 | 11.09 | 527.4 | | MS2[527.3@35]: 527.1(11), 497.2(7), 481.2(2), 375.2(75), 365.2(16),  347.2(100), 345.2(9), 335.1(3),  169.3(3) | 212 | 11.23 | 625 | MS2[625.0@35]: 605.7(100), 348.8(62) |
| 213 | 11.39 | 434.1 | | MS2[434.1@35]: 433.3(36), 425.0(77), 416.5(100) | 222 | 12.4 | 585.2 | MS2[585.2@35]: 567.1(3), 566.2(3), 541.1(2), 525.2(2), 523.5(2), 495.1(2), 423.1(100), 421.7(5), 400.7(2), 381.1(3), 378.3(3), 375.3(4), 225.1(5), 222.0(4), 180.2(6) |
| 214 | 11.72 | 386.1 ^a^ | | MS2[386.1@35]: 340.2(100), 326.4(4) | 223 | 12.05 | 711.2 | ---- |
| 215 | 11.73 | 1139.4 | | MS2[1139.4@35]: 1109.3(4), 1079.3(100), 1019.3(4), 1007.2(26), 988.7(2), 947.4(2), 901.3(3), 861.2(32), 815.4(3), 801.3(3) | 224 | 12.13 | 547.3 | MS2[547.2@35]: 325.2(4), 295.1(100), 267.3(12) |
| 216 | 11.42 | 635.3 | | MS2[635.2@35]: 617.2(4), 483.1(100), 482.3(2), 465.2(36), 423.1(16), 405.1(4), 313.2(8), 295.1(8), 271.2(5), 210.9(4) | 225 | 12.25 | 373.1 ^a^ | MS2[373.1@35]: 327.0(100), 326.2(7), 165.2(4), 160.8(6) |
| 217 | 11.84 | 329.0 | | MS2[329.0@35]: 308.1(1), 282.9(100), 281.6(10), 278.8(1), 259.1(8), 222.8(7), 211.0(2), 192.2(4), 161.2(6), 160.4(2), 159.4(3), 121.2(4), 113.0(2) | 226 | 12.61 | 449.3 | MS2[449.3@35]: 431.1(3), 311.1(2), 287.1(100), 286.4(4), 269.2(37), 259.3(37) |
| 218 | 11.95 | 612.9 | | MS2[612.9@35]: 582.2(90), 551.3(100) | 227 | 12.57 | 445.3 | MS2[445.2@35]: 445.2(28), 391.2(1), 367.3(1), 355.2(2), 325.2(100), 307.2(1) |
| 219 | 11.98 | 756.9 | | ---- | 228 | 12.86 | 295.2 | MS2[295.2@35]: 295.0(3), 251.1(100), 232.9(1), 207.1(1), 189.2(11), 183.1(1) |
| 220 | 11.99 | 635.1 | | MS2[635.2@35]: 616.8(3), 483.3(5), 473.5(3), 465.1(100), 284.9(5), 271.1(2), 242.2(2), 241.2(2) | 229 | 12.93 | 577.3 | MS2[577.3@35]: 355.0(3), 326.0(4), 325.0(100), 310.0(3), 297.0(9), 282.0(4) |
| 221 | 12.03 | 631.3 | | MS2[631.3@35]: 631.2(2), 435.0(9), 313.2(100) | 230 | 12.95 | 345.3/391 ^a^ | ---- |
| 231 | 13.00 | 371.2 | | MS2[371.2@35]: 353.0(3), 249.2(100), 248.6(3), 231.1(5), 129.4(1), 121.2(4), 113.1(4) | 240 | 13.05 | 635.2 | MS2[635.2@35]: 483.1(68), 465.2(100), 446.9(3), 313.2(19), 271.2(3), 235.1(4) |
| 232 | 13.00 | 595.3 | | MS2[595.3@35]: 577.1(7), 516.8(2), 505.1(31), 487.0(17), 475.1(100), 457.2(18), 427.2(3), 421.3(2), 415.2(23), 385.2(58), 367.3(2), 355.2(61) | 241 | 13.44 | 631.3 | MS2[631.3@35]: 631.2(11), 509.2(21), 481.2(21), 463.1(3), 435.0(7), 313.2(100), 312.6(2) |
| 233 | 13.33 | 340.1 | | MS2[340.1@35]: 340.2(5), 325.2(3), 293.9(88), 292.5(2), 187.9(100), 186.7(3), 161.1(24) | 242 | 13.37 | 635.3 | MS2[635.2@35]: 635.2(19),617.1(20), 483.1(100), 465.2(98), 423.2(73), 405.1(8),331.2(4), 321.2(2), 313.2(23), 295.2(5),271.2(20), 221.1(12), 211.2(5) |
| 234 | 13.80 | 756.8 | | ---- | 243 | 13.07 | 611.4 | MS2[611.3@35]: 611.4(2), 593.3(4), 581.3(9), 567.3(100), 566.7(2), 537.3(9), 510.4(7) |
| 235 | 13.03 | 463.3 | | MS2[463.3@35]: 463.1(7), 302.3(3), 301.3(100), 300.3(21) | 244 | 13.41 | 389.0 | ---- |
| 236 | 12.56 | 547.3 | | MS2[547.2@35]: 325.2(3), 295.2(100), 267.3(14) | 245 | 13.90 | 391.0 | MS2[391.0@35]: 376.2(2), 345.3(100), 275.6(2) |
| 237 | 13.13 | 539.1 | | MS2[539.3@35]: 493.2(100), 325.1(3) | 246 | 13.95 | 787.2 | MS2[787.2@35]: 635.1(100), 634.5(2), 617.2(30), 483.2(2), 465.1(6), 447.2(3) |
| 238 | 12.90 | 391.0 | | MS2[391.1@35]: 409.3(9), 390.6(2), 389.9(5), 355.2(14), 345.2(100), 329.1(5), 316.3(6), 229.0(6), 193.9(12), 193.0(7) | 247 | 14.00 | 415.3/461.1 ^a^ | MS2[415.2@35]: 415.2(5), 296.2(8), 295.1(100), 267.5(4) |
| 239 | 12.95 | 363.3 | | MS2[363.3@35]: 364.3(8), 363.2(100), 345.3(9), 241.2(4), 201.3(15), 183.2(4) | 248 | 14.14 | 635.2 | MS2[635.2@35]: 483.1(67), 465.1(100), 423.1(5), 405.3(3), 313.2(11), 295.2(3) |
| 249 | 14.28 | 540.9 | | MS2[540.9@35]: 603.3(21), 497.1(100), 495.3(18), 493.4(9), 481.2(14), 479.2(14), 466.0(12), 459.4(24), 453.4(24), 422.3(21), 379.3(28), 336.9(37), 197.3(47) | 257 | 14.65 | 665.3 | MS2[665.3@35]: 798.4(3), 664.8(8), 642.3(5), 619.9(5), 603.1(68), 563.1(75), 544.9(10), 521.1(100), 517.1(6), 503.1(5), 471.0(2), 415.3(5), 353.0(13), 313.1(4), 257.1(7), 249.4(5), 239.1(8) |
| 250 | 14.32 | 529.3 | | MS2[529.3@35]: 529.2(7), 511.2(19), 499.1(2), 353.1(2), 279.1(9), 267.2(100), 266.4(3), 261.1(2), 249.2(33), 231.2(7), 205.3(3) | 258 | 14.67 | 523.3 | MS2[523.2@35]: 505.1(2), 361.0(25), 360.3(3), 343.1(12), 341.1(80), 270.9(2), 253.0(15), 235.2(3), 225.1(4), 207.0(2), 199.2(100), 181.2(3), 178.8(10), 169.2(1) |
| 251 | 14.38 | 525.2 ^a^ | | MS2[525.3@35]: 479.2(100) | 259 | 14.69 | 348.9 | ---- |
| 252 | 14.55 | 787.2 | | MS2[787.1@35]: 635.1(12), 617.1(100), 465.2(2) | 260 | 14.72 | 565.3 | MS2[565.3@35]: 445.1(6), 419.0(5), 272.3(2), 271.2(100), 270.5(2), 177.1(2) |
| 253 | 14.27 | 387.2 | | MS2[387.2@35]: 405.7(1), 386.6(1), 369.2(1), 340.9(1), 253.3(1), 249.1(100), 248.5(3), 137.1(48), 113.0(1) | 261 | 14.76 | 429.3 | MS2[429.3@35]: 429.1(100), 411.4(2), 385.2(11), 369.1(4), 340.3(2), 325.0(3), 307.3(1), 264.8(2), 178.8(4), 177.8(2) |
| 254 | 14.57 | 563.3 | | MS2[563.2@35]: 563.0(2), 503.2(8), 473.2(8), 443.1(6), 383.2(2), 353.2(4), 341.1(4), 312.1(2), 311.2(100), 283.3(8) | 262 | 14.84 | 491 ^a^ | MS2[491.1@35]: 445.1(22), 444.2(1), 283.2(100) |
| 255 | 14.62 | 635.2 | | MS2[635.2@35]:635.1(5), 483.1(100), 465.3(45), 423.2(4), 313.2(11), 271.2(3) | 263 | 14.85 | 849.3 | MS2[849.3@35]: 849.3(76), 831.2(15), 759.2(2), 717.2(31), 627.1(2), 597.2(4), 555.3(100), 537.2(19), 435.1(5),  409.4(12), 391.4(3), 341.3(2), 299.1(11), 271.2(3) |
| 256 | 14.63 | 769.4 | | MS2[769.4@35]: 707.1(2), 667.2(17), 625.3(100), 607.3(27) | 264 | 14.98 | 431.3 | MS2[431.3@35]: 431.1(12), 311.2(100), 310.6(2) |
| 267 | 15.35 | 519.3 | | MS2[519.3@35]: 473.0(100), 307.1(8), 163.1(2) | 274 | 15.29 | 463.1 | MS2[463.2@35]: 437.9(1), 343.2(1), 339.1(1), 300.8(2), 283.0(100), 239.2(22), 225.3(6), 219.2(1), 217.1(1), 191.3(1), 177.2(18), |
| 268 | 15.03 | 563.4 | | MS2[595.2@35]: 551.1(100), 532.9(2), 469.2(7), 297.2(5), 253.3(53), 178.9(3) | 275 | 15.33 | 477.2 | MS2[477.1@35]: 477.0(4), 433.0(100), 389.1(5), 387.1(5), 371.0(4), 266.6(6) |
| 269 | 15.24 | 497.4 ^a^ | | MS2[497.4@35]: 451.5(100), 450.8(1) | 276 | 15.34 | 707.2 | MS2[707.2@35]: 698.4(2), 645.0(2), 605.2(2), 563.1(88), 545.2(100), 442.8(2) |
| 270 | 15.03 | 595.2 | | MS2[595.2@35]: 551.1(100), 532.9(2), 469.2(7), 297.2(5), 253.3(53), 178.9(3) | 277 | 15.39 | 405.2 | MS2[405.1@35]: 243.2(100) |
| 271 | 15.24 | 687.2 ^a^ | | MS2[687.2@35]: 687.9(6), 669.1(5), 641.1(100), 639.6(4), 623.2(7), 610.9(81), 592.9(44), 535.0(7), 525.6(4), 519.0(8), 514.6(4), 488.6(7), 444.6(3), 425.8(8), 398.1(5), 396.1(11), 357.1(5), 282.8(5) | 278 | 15.14 | 565.3 | MS2[565.3@35]: 547.3(2), 445.2(4), 419.0(9), 313.2(2), 295.1(2), 272.1(5), 271.2(100) |
| 272 | 15.27 | 569.3 | | MS2[569.3@35]: 509.2(33), 479.3(5), 449.1(7), 407.2(100), 405.2(8), 389.3(50), 387.3(10), 373.3(3), 363.2(42), 361.3(8), 359.3(3), 345.3(15), 343.3(4), 341.8(2), 335.4(8), 318.8(6), 317.2(3), 301.3(15), 299.3(2), 285.3(2) | 279 | 15.37 | 787.2 | MS2[787.2@35]: 635.1(100), 617.2(28), 483.1(2), 465.2(6), 447.2(3), 423.0(2), 295.1(2) |
| 273 | 15.28 | 737.3 | | MS2[737.3@35]: 719.5(1), 692.2(1), 690.6(1), 675.5(1), 647.3(2), 635.2(9), 617.1(4), 593.2(100), 515.1(5), 503.2(3), 489.0(1), 485.3(7), 473.2(12), 455.2(3) | 280 | 15.38 | 543.2 | MS2[543.2@35]: 542.2(1), 403.2(100), 257.3(1), 256.4(1), 255.3(1) |
| 281 | 15.59 | 433.2 | | MS2[433.2@35]: 313.2(9), 271.2(100), 151.2(2) | 290 | 15.86 | 415.2 | MS2[415.3@35]: 416.1(2), 415.2(13), 319.4(2), 296.2(17), 295.1(100), 282.9(2) |
| 282 | 15.35 | 309.1 | | MS2[519.3@35]: 473.0(100), 307.1(8), 163.1(2) | 291 | 16.02 | 737.3 | MS2[737.3@35]: 675.1(2), 635.1(3), 617.1(2), 593.2(13), 575.2(9), 557.2(4), 503.2(18), 473.2(100), 383.3(5), 353.2(6) |
| 283 | 15.54 | 793.4 | | ---- | 292 | 16.18 | 623.3 | MS2[623.3@35]: 487.1(6), 461.2(100), 443.2(5), 315.2(2), 297.1(7), 279.0(2), 261.2(3), 249.2(4) |
| 284 | 15.63 | 461.3 | | MS2[461.3@35]: 462.4(2), 461.2(10), 417.5(2), 371.1(1), 342.2(5), 341.2(100), 340.4(2), 328.8(1), 326.1(1), 313.3(1), 307.4(1), 293.2(1), 266.0(5) | 293 | 16.19 | 737.3 | MS2[737.3@35]: 719.2(10), 677.1(3), 661.2(3), 659.0(2), 657.3(2), 647.1(81), 635.1(7), 617.1(88), 593.3(30), 575.2(14), 503.3(9), 485.3(8), 473.2(100), 467.4(2), 455.2(11), 413.3(13), 383.2(41), 353.2(39) |
| 285 | 15.64 | 625 ^a^ | | MS2[625.0@35]: 589.0(28), 503.3(32), 454.9(26), 448.2(37), 380.7(100) | 294 | 15.81 | 417.2 | MS2[417.2@35]: 255.2(100), 254.2(2) |
| 286 | 15.69 | 711.3 | | ---- | 295 | 16.24 | 257.2 | MS2[417.2@35]: 255.2(100), 254.2(2) |
| 287 | 15.77 | 787.1 | | MS2[787.1@35]: 635.1(100), 617.2(26), 483.1(3), 465.2(5), 447.1(2), 423.2(2), 295.0(2) | 296 | 16.19 | 525.2 ^a^ | MS2[525.1@35]: 479.0(100), 478.3(3), 435.1(3), 357.2(9), 317.1(2), 283.0(5) |
| 288 | 15.81 | 605.3 | | MS2[605.2@35]: 331.2(1), 297.1(100), 296.3(1), 253.2(21), 211.1(1) | 297 | 16.29 | 417.2 | MS2[417.2@35]: 255.2(100), 135.2(3) |
| 289 | 15.80 | 433.2 | | MS2[433.2@35]: 313.1(10), 271.1(100), 151.2(3) | 298 | 16.26 | 707.2 | MS2[707.3@35]: 645.3(6), 616.9(2), 605.2(13), 563.2(69), 545.2(18), 527.1(8), 503.1(6), 485.2(2), 473.2(23), 443.2(100), 442.6(6), 425.1(4), 383.2(8), 353.3(8) |
| 299 | 16.30 | 787.2 | | MS2[787.2@35]: 635.1(47), 617.1(100), 573.3(2), 465.2(11), 447.2(2), 403.3(2) | 307 | 17.00 | 687.2 ^a^ | MS2[687.2@35]: 641.1(100), 640.2(3), 519.0(11), 465.1(2), 427.0(2), 375.1(3) |
| 300 | 16.39 | 687.3 ^a^ | | MS2[687.3@35]: 668.9(3), 641.1(100), 640.4(3), 519.1(4), 485.6(2), 357.3(2) | 308 | 16.14 | 674.3 | MS2[674.3@35]: 590.9(3), 549.2(100), 531.2(52), 417.1(8), 399.1(5), 255.2(9) |
| 301 | 16.43 | 783.3 | | MS2[783.3@35]: 661.2(29), 643.0(3), 631.2(2), 617.2(100), 465.1(90), 313.2(9) | 309 | 16.17 | 577.3 | MS2[577.3@35]: 577.2(34),559.2(17), 517.2(3) 503.2(35), 499.3(2), 488.2(10), 487.2(30), 474.2(7), 473.2(41), 457.2(100), 439.4(3), 413.2(6), 383.2(24),353.3(17) |
| 302 | 16.55 | 563.2 | | MS2[563.2@35]: 564.4(3), 563.2(13), 546.4(4), 545.2(20), 533.3(3), 515.1(3), 504.3(5), 503.2(47), 485.3(4), 474.3(7), 473.2(92), 455.2(4), 444.2(11), 443.2(100), 425.2(5), 412.9(2), 383.2(26), 379.2(2), 365.3(2), 354.3(3), 353.2(20) | 310 | 17.07 | 786.9 | ---- |
| 303 | 16.57 | 707.3 | | MS2[707.3@35]: 645.0(3), 605.1(4), 563.1(20), 545.0(6), 527.2(4), 473.2(26), 443.1(100), 442.3(2), 383.2(9), 353.2(8) | 311 | 16.12 | 542.3 | MS2[542.3@35]: 459.1(3), 435.9(1), 417.2(29), 399.2(15), 255.2(100) |
| 304 | 16.63 | 449.4 | | MS2[449.4@35]: 287.2(6), 269.1(100), 251.3(4), 224.9(3), 209.2(3), 189.2(2) | 312 | 16.38 | 542.3 | MS2[542.3@35]: 459.1(3), 417.1(41), 399.3(7), 255.2(100), 254.5(1) |
| 305 | 16.64 | 787.2 | | MS2[787.1@35]: 635.1(5), 617.1(100) | 313 | 16.46 | 579.4 | MS2[579.4@35]: 459.1(16), 429.1(19), 327.1(15), 309.1(2), 286.1(12), 285.2(100) |
| 306 | 16.94 | 361.1 | | MS2[361.1@35]: 317.1(100), 305.0(2) | 314 | 16.32 | 577.3 | MS2[577.3@35]: 578.3(17), 577.2(40), 560.1(4), 559.2(15), 503.2(21), 499.3(4), 487.2(33), 474.3(4), 473.2(23), 458.2(21), 457.2(100), 383.2(16), 353.2(15) |
| 315 | 16.54 | 674.4 | | MS2[674.4@35]: 674.3(15), 591.2(3), 554.2(13), 549.2(100), 542.2(15), 531.2(17), 429.0(3), 386.9(2), 297.1(6), 255.2(6) | 323 | 16.90 | 828.7 ^a^ | MS2[783.3@35]: 661.1(28), 643.2(4), 631.3(4), 617.2(100), 616.5(2), 569.3(2), 465.2(96), 313.2(10) |
| 316 | 16.65 | 787.2 | | MS2[787.1@35]: 635.1(10), 617.1(100), 465.1(2) | 324 | 17.15 | 525.1 | MS2[525.1@35]: 525.2(3), 478.9(49), 477.7(3), 449.0(100), 411.9(7), 161.0(3) |
| 317 | 16.75 | 674.4 | | MS2[674.4@35]: 674.2(5), 591.1(6), 554.3(3), 549.2(100), 542.3(10), 531.2(14), 429.2(3), 417.3(2), 399.3(2), 297.2(4), 255.2(11) | 325 | 17.15 | 939.2 | MS2[939.2@35]: 787.0(9), 769.1(100), 768.4(2), 617.1(6), 601.3(2), 599.1(3) |
| 318 | 16.82 | 565.3 | | MS2[565.3@35]: 547.3(19), 434.1(4), 433.1(100), 416.3(2), 415.2(23), 403.3(2), 272.1(2), 271.1(95) | 326 | 17.17 | 887.2 | MS2[887.2@35]: 725.2(100), 693.4(2), 549.2(8), 531.2(4) |
| 319 | 16.87 | 415.2 | | MS2[415.2@35]: 296.2(12), 295.2(100) | 327 | 17.13 | 903.1 | ---- |
| 320 | 16.91 | 787.2 | | MS2[787.1@35]: 635.2(46), 617.2(100), 616.5(2), 465.2(16), 447.1(7), 295.1(2) | 328 | 17.27 | 315.2 | MS2[315.2@35]: 315.1(3), 271.2(100) |
| 321 | 16.93 | 631.3 | | MS2[631.3@35]: 613.2(100), 612.6(4), 601.2(7), 585.2(2), 583.3(2), 509.3(14), 491.2(21), 479.2(13), 465.3(19), 463.2(3), 399.2(7), 375.2(3), 313.3(7), 271.2(16) | 329 | 17.34 | 631.4 | MS2[631.2@35]: 631.2(64), 613.1(24), 600.0(3), 587.1(3), 508.8(5), 465.1(4), 417.3(8), 313.2(100), 295.3(2), 271.0(4), 223.2(2) |
| 322 | 16.93 | 509.1 | | MS2[509.1@35]: 463.2(100), 462.5(5), 357.0(2) | 330 | 17.36 | 721.3 | MS2[721.3@35]: 659.2(6), 619.1(4), 577.2(10), 559.2(5), 541.1(3), 487.2(20), 457.2(100), 383.3(5), 353.2(6) |
|  |  |  | |  |  |  |  |  |
|  |  |  | |  |  |  |  |  |
| 331 | 17.48 | 519.3 | | MS2[519.5@35]: 501.2(100) | 338 | 17.56 | 707.3 | MS2[707.2@35]: 645.2(4), 605.2(2), 563.2(19), 545.3(5), 527.1(6), 473.2(24), 443.2(100), 413.3(2), 383.3(5), 353.1(10) |
| 332 | 17.49 | 681.0 | | MS2[681.0@35]: 682.2(16), 643.9(50), 637.0(36), 597.5(29), 510.7(100), 401.4(31) | 339 | 17.68 | 671.2 | ---- |
| 333 | 17.63 | 861.2 | | MS2[861.2@35]: 843.0(2), 741.3(2), 699.1(100), 693.2(4), 591.0(2), 549.2(5), 531.3(7), 279.1(2) | 340 | 17.87 | 491.3 | MS2[491.3@35]: 447.1(100), 324.7(3), 300.0(2), 264.9(13), 214.5(2), 205.1(13), 165.1(3), 162.9(79), 144.7(2),  142.9(2) |
| 334 | 17.08 | 857.2 | | MS2[857.3@35]: 695.2(100), 549.2(3), 531.1(2) | 341 | 18.11 | 721.3 | MS2[721.3@35]: 703.1(2), 659.1(5), 619.3(6), 577.2(16), 559.2(6), 541.1(6), 487.2(20), 457.2(100), 383.3(5), 353.2(4) |
| 335 | 17.17 | 887.3 | | MS2[887.2@35]: 725.2(100), 693.4(2), 549.2(8), 531.2(4) | 342 | 17.88 | 465.1 ^a^ | MS2[465.1@35]: 419.0(100), 418.0(2), 257.2(15) |
| 336 | 17.52 | 475.3 | | MS2[475.3@35]: 493.2(2), 475.4(4), 474.5(6), 460.3(2), 457.2(2), 437.2(3), 431.0(24), 429.9(2), 413.0(49), 412.3(6), 401.1(3), 372.9(100), 331.3(18), 313.2(4), 142.7(11), 141.7(3) | 343 | 17.96 | 591.3 | MS2[591.2@35]: 549.2(100), 531.2(50), 513.1(3), 473.3(2), 417.3(10), 399.2(7), 255.3(8), 254.6(4) |
| 337 | 17.46 | 783.2 | | MS2[783.2@35]: 765.1(76), 737.2(8), 661.0(13), 643.1(42), 631.2(11), 617.2(10), 615.2(12), 587.4(2), 551.0(2), 465.2(100), 313.1(6) | 344 | 17.99 | 561.3 | MS2[561.3@35]: 375.2(1), 339.1(2), 310.2(3), 309.1(100), 294.0(1), 281.2(10), 266.1(4) |
| 345 | 18.02 | 429.2 | | MS2[429.2@35]: 429.1(23), 339.0(2), 309.1(100), 308.5(2), 281.2(4) | 351 | 18.28 | 469.2 | MS2[469.2@35]: 770.0(22), 769.0(62), 618.0(4), 616.9(14), 601.1(2), 469.2(11), 468.2(9), 393.3(100), 384.4(3), 362.3(2), 317.5(17), 169.2(26) |
| 346 | 18.22 | 721.3 | | MS2[721.3@35]: 659.2(5), 619.2(10), 577.2(27), 559.1(9), 541.3(6), 487.2(17), 457.2(100), 383.3(4), 353.2(7) | 352 | 18.27 | 939.2 | MS2[939.2@35]: 787.0(10), 770.1(7), 769.0(100), 617.2(8), 599.1(2) |
| 347 | 18.06 | 903.2 | | MS2[903.2@35]: 1191.2(5), 893.0(10), 871.8(9), 867.1(28), 859.3(100), 852.1(12), 822.8(6), 821.1(9), 817.5(5), 796.2(13), 787.3(16), 782.1(19), 740.8(39), 697.0(5), 662.7(9), 651.0(8), 494.0(12), 451.4(12), 353.2(12) | 353 | 18.32 | 569.4 | MS2[569.3@35]: 569.3(100), 555.2(4), 554.2(10), 509.2(5), 450.3(2), 449.2(3), 419.1(2), 417.2(4), 408.1(5), 407.3(38), 406.4(2), 392.2(4), 381.3(5), 312.1(2), 311.1(12), 301.1(2), 269.2(2),  229.0(2) |
| 348 | 18.15 | 417.2 | | MS2[417.1@35]: 373.1(100), 270.1(2) | 354 | 18.36 | 137.2 | MS2[137.1@35]: 137.1(100), 93.3(20) |
| 349 | 18.22 | 137.0 | | MS2[137.2@35]: 137.1(100), 93.2(26) | 355 | 18.30 | 301.2 | MS2[301.2@35]: 301.1(94), 286.0(54), 283.2(4), 270.1(2), 269.3(100), 257.2(13), 242.3(32), 225.1(2) |
| 350 | 18.25 | 465.1 | | MS2[465.1@35]: 418.9(100), 418.1(5), 257.2(18) | 356 | 18.39 | 579.3 | MS2[579.2@35]: 562.2(2), 323.0(2), 285.4(2), 284.4(9), 282.9(2), 255.2(100) |
|  |  |  | |  | 357 | 18.46 | 631.3 | MS2[631.3@35]: 632.3(3), 631.2(100), 613.2(5), 601.3(14), 585.1(2), 509.3(29), 503.3(2), 481.3(17), 479.4(2), 465.4(2), 463.2(12), 435.3(3), 417.4(2), 399.3(3), 313.2(70), 273.1(2), 271.2(3) |
|  |  |  | |  |  |  |  |  |
| 358 | 18.5 | 609.4 | | MS2[469.2@35]: 770.0(22), 769.0(62), 618.0(4), 616.9(14), 601.1(2), 469.2(11), 468.2(9), 393.3(100), 384.4(3), 362.3(2), 317.5(17), 169.2(26) | 363 | 18.63 | 707.2 | MS2[707.2@35]: 689.5(13), 680.9(8), 647.2(67), 617.1(100), 605.2(18), 563.3(14), 555.6(10), 544.8(6), 515.0(4), 503.2(19), 473.3(19), 466.9(21), 443.0(60), 431.6(5), 425.2(7), 413.3(10), 387.3(3), 383.1(55), 364.7(7), 353.4(19) |
| 359 | 18.51 | 553.3 | | MS2[137.1@35]: 137.1(100), 93.3(20) | 364 | 18.94 | 721.3 | MS2[721.3@35]: 659.3(3), 647.2(9), 619.2(10), 617.1(48), 577.3(28), 559.3(21), 541.4(3), 515.2(2), 487.3(9), 485.2(3), 473.2(7), 457.2(100), 455.2(6), 413.3(3), 383.3(11), 353.3(21) |
| 360 | 18.54 | 917.2 | | MS2[917.2@35]: 899.1(2), 755.2(100), 754.4(6), 711.7(2), 693.0(4), 573.4(4), 549.3(10), 531.3(9), 504.6(2) | 365 | 18.82 | 965.6 | MS2[965.5@35]: 965.4(10), 947.5(76), 938.4(6), 921.5(100), 905.4(5), 904.3(13), 903.4(15), 877.4(4), 860.4(4), 856.2(2), 833.5(3), 824.6(3), 813.5(3), 811.5(3), 787.5(4), 786.3(4), 785.3(23), 741.5(12), 715.2(3), 706.4(4), 688.3(3), 661.3(4), 630.4(9), 579.4(10), 535.4(13), 381.3(4) |
| 361 | 18.55 | 691.1 | | MS2[691.0@35]: 691.7(100), 690.9(33), 641.2(27), 634.7(40), 488.0(32) | 366 | 18.77 | 537.5 | MS2[537.5@35]: 537.4(100), 519.5(1), 418.2(2), 417.3(7), 389.3(2), 376.4(13), 375.4(79), 374.4(11), 373.5(9), 355.4(2) |
| 362 | 18.57 | 632.9 ^a^ | | MS2[632.8@35]: 632.6(23), 609.9(86), 584.6(33), 517.4(68), 511.2(58), 504.2(44), 271.2(100) | 367 | 18.98 | 565.4 | MS2[565.4@35]: 565.3(99), 522.2(3), 521.1(15), 504.2(4), 497.2(12), 479.1(8), 445.3(3), 438.7(2), 434.3(6), 433.1(43), 389.1(6), 313.2(14), 299.1(4), 271.1(100), |
|  |  |  | |  |  |  |  | 228.3(5), 227.2(45), 177.1(11) |
| 368 | 18.77 | 631.3 | | MS2[631.3@35]: 631.2(100), 613.2(8), 601.3(11), 509.2(41), 503.2(2), 491.3(3), 481.3(19), 465.2(4), 463.2(8), 399.3(3), 331.2(2), 313.2(81), 273.1(2), 271.2(2) | 377 | 19.48 | 635.1 | MS2[635.2@35]: 634.2(8), 603.1(10), 591.2(100), 574.1(19), 572.9(15), 553.6(20), 547.5(14), 482.8(9), 465.1(35), 444.8(9), 420.8(12), 401.1(10), 378.0(22), 294.6(8), 289.1(13) |
| 369 | 18.85 | 433.3 | | MS2[433.3@35]: 271.2(100) | 376 | 19.20 | 187.3 | ---- |
| 370 | 18.98 | 687.1 ^a^ | | MS2[687.0@35]: 687.3(100), 641.5(27), 424.5(34), 337.2(28) | 378 | 19.46 | 645.3 | MS2[645.3@35]: 627.1(100), 615.2(5), 597.2(4), 523.2(8), 505.2(8), 493.2(24), 477.3(2), 327.2(5), 285.2(5) |
| 371 | 19.04 | 981.2 | | MS2[981.0@35]: 924.9(40), 853.4(100), 818.9(15), 784.4(75), 673.3(46) | 379 | 19.47 | 1131.7 | MS2[1131.6@35]: 1113.6(5),497.2(100), 452.6(7), 435.2(4), 405.0(14),  320.9(20) |
| 372 | 19.11 | 301.2 | | MS2[301.2@35]: 301.1(38), 300.5(2), 286.1(17), 269.2(60), 191.2(100), 177.3(6), 161.3(2), 151.3(5) | 380 | 19.53 | 509.2 ^a^ | MS2[509.2@35]: 464.0(2), 463.0(100), 341.3(12) |
| 373 | 19.18 | 733.1 | | MS2[733.2@35]: 714.9(1), 557.1(100), 513.3(1) | 381 | 19.54 | 630.2 | MS2[630.1@35]: 629.4(37), 599.2(14), 584.0(100), 405.5(16), 374.4(8), 297.3(15), 287.0(17) |
| 374 | 19.15 | 509.1 ^a^ | | MS2[509.2@35]: 509.1(25), 464.9(4), 463.1(100), 462.3(10), 461.4(4), 436.2(5), 399.4(16), 341.1(5), 328.1(5),  179.1(5) | 382 | 19.58 | 591.2 | MS2[591.2@35]: 549.2(100), 548.6(4), 531.2(62), 459.1(2), 417.2(7), 399.2(7), 255.2(12), 254.5(3) |
| 375 | 19.20 | 269.3 | | MS2[269.2@35]: 270.2(14), 269.2(100), 241.2(2) | 383 | 19.63 | 711.3 | MS2[711.3@35]: 591.1(2), 565.3(100), 547.3(28), 415.5(2) |
| 384 | 19.67 | 269.3 | | MS2[269.3@35]: 270.2(12), 269.2(100), 225.4(2) | 392 | 19.95 | 509.4/555 ^a^ | MS2[509.4@35]: 509.3(46), 492.3(23), 491.5(7), 470.6(3), 465.3(35), 448.3(11), 447.4(4), 404.2(2), 361.3(2), 346.2(7), 344.1(14), 335.4(5), 334.4(6), 318.1(4), 266.2(100), 249.2(15), 232.1(2), 205.3(4) |
| 385 | 19.67 | 723.6 ^a^ | | MS2[723.6@35]: 677.6(100) | 393 | 19.98 | 799.3 | MS2[799.3@35]: 725.0(33), 605.2(7), 549.3(3), 531.2(100), 417.2(6), 399.3(14), 255.3(10), 254.1(2) |
| 386 | 19.68 | 509.4 ^a^ | | MS2[509.1@35]: 509.3(22), 463.0(100), 448.3(5), 346.2(4), 344.3(3), 341.1(13), 335.2(2), 266.3(40), 249.3(6) | 394 | 19.79 | 719.8 | MS2[719.9@35]: 626.1(100), 520.7(83) |
| 387 | 19.71 | 799.3 | | MS2[799.3@35]: 725.1(25), 605.1(2), 549.3(2), 531.2(100), 459.4(2), 417.1(6), 399.3(18), 255.2(10), 253.2(3) | 395 | 20.22 | 719.9 | MS2[720.0@35]: 714.1(100), 701.0(51), 631.2(57) |
| 388 | 19.93 | 464.9 ^a^ | | MS2[465.0@35]: 418.9(100), 257.2(13) | 396 | 20.13 | 713.3 | ---- |
| 389 | 19.97 | 743.3 | | MS2[743.2@35]: 743.2(1), 699.2(100), 698.5(3), 671.3(13), 549.4(1), 531.2(3), 399.2(1), 387.1(1), 377.0(2), 368.9(1) | 397 | 20.14 | 661.6 | MS2[953.2@35]: 801.2(4), 783.0(13), 769.1(100), 617.2(6), 599.0(2), 447.1(2) |
| 390 | 20.06 | 963.2 | | MS2[963.2@35]: 944.9(2), 837.1(7), 753.0(7), 735.1(5), 675.2(6), 633.0(14), 631.3(3), 591.2(100), 549.3(66),  531.0(9) | 398 | 20.19 | 985.5 | MS2[985.5@35]: 1505.3(8), 1020.0(5), 967.4(5), 923.2(12), 879.3(11), 851.0(10), 351.1(100) |
| 391 | 19.72 | 631.3 | | MS2[631.3@35]: 613.2(100), 601.2(4), 585.2(2), 509.3(9), 491.3(12), 479.2(9), 465.2(7), 399.2(4), 313.3(4), 271.2(10) | 399 | 20.14 | 953.2 | MS2[953.2@35]: 801.2(4), 783.0(13), 769.1(100), 617.2(6), 599.0(2), 447.1(2) |
| 400 | 20.22 | 471 ^a^ | | MS2[471.1@35]: 425.1(100), 402.9(9), 338.8(5) | 410 | 20.50 | 507.2 ^a^ | MS2[507.2@35]: 461.0(100), 460.3(7), 431.1(19), 339.2(89), 283.2(5), 179.1(3), 177.2(10) |
| 401 | 20.27 | 711.3 | | MS2[711.3@35]: 591.2(4), 577.2(2), 565.3(100), 564.4(3), 549.3(18), 547.3(47), 433.1(2), 415.5(3) | 411 | 20.55 | 1065.3 | MS2[1065.3@35]: 1047.1(27), 945.0(20), 903.1(100), 899.1(25), 885.2(30), 531.0(18), 417.0(20) |
| 402 | 20.41 | 953.2 | | MS2[953.2@35]: 801.2(4), 783.0(13), 769.1(100), 617.2(6), 599.0(2),  447.1(2) | 412 | 20.57 | 318.9 ^a^ | MS2[318.9@35]: 318.1(36), 291.2(29), 290.1(100), 246.2(38) |
| 403 | 20.34 | 433.3 | | MS2[433.3@35]: 433.2(52), 418.2(7), 403.1(100), 385.2(10), 373.2(90) | 413 | 20.75 | 1049.4 | MS2[1049.2@35]: 629.0(40), 569.2(100) |
| 404 | 20.39 | 600.2 | | MS2[600.2@35]: 267.2(100), 252.4(8) | 414 | 20.78 | 606.9 | ---- |
| 405 | 20.64 | 600.2 | | MS2[600.3@35]: 267.2(100), 252.3(12) | 415 | 20.85 | 733.2 | MS2[733.2@35]: 557.1(100), 351.3(1), 309.0(1) |
| 406 | 20.40 | 969.5 | | MS2[969.5@35]: 971.6(3), 952.4(14), 951.3(19), 908.7(4), 589.1(2), 474.4(4), 352.2(9), 351.1(100) | 416 | 20.49 | 407.4 | MS2[407.4@35]: 363.2(100), 345.3(15), 335.3(53), 319.1(8), 308.9(4), 301.3(7), 291.3(16), 290.5(2) |
| 407 | 20.40 | 674.4 | | MS2[674.3@35]: 656.5(2), 655.5(2), 629.9(5), 554.2(2), 549.2(87), 531.1(100), 482.6(2), 399.5(4), 255.1(5) | 417 | 20.59 | 743.3 | MS2[743.3@35]: 699.2(100), 531.2(6) |
| 408 | 20.41 | 1131.6 | | MS2[1131.6@35]: 1113.3(11), 967.3(3), 497.3(100), 435.1(2), 339.0(5) | 418 | 20.67 | 505.1 ^a^ | MS2[505.2@35]: 459.0(100), 339.0(3), 293.1(10) |
| 409 | 20.42 | 571.4 | | MS2[571.4@35]: 572.2(36), 571.2(100), 556.2(3), 314.3(2), 313.2(31), 257.3(4), 243.2(2), 169.2(5) | 419 | 20.95 | 743.2 | MS2[743.2@35]: 699.2(100), 645.1(2), 508.3(2), 429.9(2) |
| 420 | 20.79 | 623.4 | | MS2[623.4@35]: 605.1(9), 587.3(59), 569.4(3), 561.5(4), 557.2(2), 551.3(2), 543.3(21), 539.4(2), 533.3(41), 525.4(5), 515.3(100), 501.4(8) | 425 | 21.55 | 549.3 | MS2[549.4@35]: 550.3(2), 549.2(11), 429.1(15), 417.1(33), 357.2(2), 327.2(2), 297.1(26), 269.2(4), 256.3(3), 255.2(100) |
| 421 | 20.91 | 669.3 | | MS2[669.3@35]: 549.2(26), 531.2(100), 523.4(2), 399.0(2), 255.2(8) | 426 | 21.03 | 591.2 | MS2[591.2@35]: 591.3(2), 547.3(11), 546.6(5), 439.1(21), 435.2(2), 411.2(6), 369.0(3), 343.0(2), 301.1(72), 296.9(9), 289.2(100), 282.2(3), 257.2(12), 255.2(2), 245.2(7), 215.2(25), 213.2(5), 202.0(6), 179.3(3) |
| 422 | 21.31 | 563.2 | | MS2[563.2@35]: 887.2(1), 598.5(1), 563.5(1), 562.7(2), 545.2(1), 535.5(2), 519.0(3), 508.0(1), 503.2(2), 501.5(2), 500.8(4), 494.3(1), 413.0(4), 393.2(1), 325.0(1), 307.2(15), 297.2(2),  255.2(100) | 427 | 21.15 | 783.3 | MS2[783.3@35]: 783.2(28), 765.0(22), 753.2(3), 737.1(24), 661.1(21), 643.2(26), 632.2(5), 631.2(100), 615.4(5), 613.2(20), 551.4(15), 509.2(3), 491.3(7), 483.3(8), 465.1(13), 423.1(7), 328.8(3), 313.2(6) |
| 423 | 21.06 | 549.3 | | MS2[549.3@35]: 549.2(15), 519.0(2), 429.1(14), 418.1(2), 417.2(41), 399.1(4), 387.0(3), 327.0(2), 297.1(29), 256.2(2), 255.2(100), 254.1(3) | 428 | 21.01 | 475.1 | MS2[475.1@35]: 428.6(5), 267.2(100) |
| 424 | 21.66 | 445.1 | | MS2[445.0@35]: 269.2(100), 175.0(10), 173.9(2) | 429 | 21.22 | 519 | MS2[519.0@35]: 519.7(5), 518.1(2), 501.4(3), 500.2(2), 472.8(100), 471.7(3), 361.6(2), 311.1(45), 231.6(3) |
| 430 | 21.38 | 1131.7 | | MS2[1131.6@35]: 1113.3(11), 967.3(3), 497.3(100), 435.1(2), 339.0(5) | 431 | 21.48 | 987.1 | ---- |
| 432 | 21.10 | 895.3 | | MS2[895.3@35]: 877.1(8), 847.1(14), 753.1(7), 699.2(100), 531.2(15) | 440 | 21.73 | 985.6 | MS2[985.6@35]: 923.0(31), 496.9(100), 351.1(63) |
| 433 | 21.47 | 783.4 | | MS2[783.4@35]: 765.2(100), 661.3(4), 643.3(37), 625.4(2), 587.1(5), 465.2(21), 313.2(3) | 441 | 21.84 | 783.3 | MS2[783.3@35]: 783.4(3), 774.2(2), 661.1(31), 643.1(11), 631.1(34),  617.2(38), 613.1(28), 601.0(2), 509.2(7), 491.1(16), 466.1(2), 465.2(100), 337.2(2), 313.3(6), 271.2(6) |
| 434 | 21.62 | 820.3 | | MS2[820.3@35]: 700.2(5), 695.1(11), 674.2(100), 656.2(61), 542.2(4), 531.3(4), 524.3(6) | 442 | 21.71 | 813.3 | MS2[813.3@35]: 549.2(100), 531.2(13), 417.1(2) |
| 435 | 21.68 | 571.3 | | MS2[571.3@35]: 572.2(44), 571.2(100), 556.2(2), 313.2(39), 169.2(6) | 443 | 21.90 | 637.0 | MS2[636.9@35]: 637.1(100), 635.9(95), 565.4(66), 547.1(41), 503.6(64), 341.1(33), 324.0(80) |
| 436 | 21.70 | 417.4 | | MS2[417.3@35]: 297.0(16), 255.2(100) | 444 | 21.71 | 669.3 | MS2[631.3@35]: 631.2(7), 613.1(2), 509.2(23), 491.1(2), 473.3(2), 465.2(100), 399.2(11), 313.3(16), 271.3(7),  211.3(6) |
| 437 | 21.71 | 631.3 | | MS2[813.3@35]: 549.2(100), 531.2(13), 417.1(2) | 445 | 21.85 | 891 ^a^ | MS2[891.0@35]: 445.0(100), 401.2(4), 269.1(40) |
| 438 | 22.29 | 417.2 | | MS2[417.3@35]: 255.2(100) | 446 | 21.89 | 755.4 | MS2[755.4@35]: 575.0(4), 563.2(2), 549.2(100), 531.2(68), 513.0(3), 417.3(5), 399.3(11), 255.1(7) |
| 439 | 22.18 | 741.1 | | MS2[741.1@35]: 740.3(25), 702.6(88), 626.2(68), 588.8(66), 521.8(100) | 447 | 22.02 | 1049.3 | MS2[1049.2@35]: 629.0(40), 569.2(100) |
| 448 | 22.11 | 783.3 | | MS2[783.2@35]: 765.1(100), 764.5(2), 747.2(2), 661.1(5), 643.2(38), 631.2(2), 617.2(2), 587.2(3), 491.2(2), 465.2(28), 313.3(2) | 455 | 22.48 | 491.2 | MS2[491.2@35]: 445.1(100), 292.8(2) |
| 449 | 22.05 | 615.3 | | MS2[615.3@35]: 615.0(78), 597.0(34), 586.0(5), 579.0(30), 571.0(2), 569.0(2), 493.0(100), 475.0(37), 463.0(3), 449.0(3), 417.0(17), 375.0(12), 341.0(4), 313.0(31), 271.0(15), 253.0(2), 211.0(2) | 456 | 21.91 | 1115.7 | MS2[1115.7@35]: 1169.5(12), 1097.2(18), 1071.1(12), 497.2(100), 338.9(5) |
| 450 | 22.09 | 921.3 | | MS2[921.3@35]: 903.1(7), 873.1(12), 779.1(7), 725.2(100), 724.4(4), 549.4(3), 531.2(13) | 457 | 22.38 | 965.2 | ---- |
| 451 | 22.25 | 921.3 | | MS2[921.3@35]: 873.0(3), 779.1(2), 725.1(100), 724.4(2), 549.2(4), 531.3(8) | 458 | 22.44 | 569.3 | MS2[569.3@35]: 569.0(4), 475.2(16), 460.2(2), 450.1(3), 449.1(56), 430.1(10), 429.2(100), 415.8(2), 414.2(13), 335.2(4), 310.1(5), 309.1(87), 294.4(6) |
| 452 | 22.26 | 417.3 | | MS2[417.1@35]: 255.2(100) | 459 | 22.47 | 1115.7 | MS2[1115.6@35]: 1097.5(25), 1096.9(9), 951.3(3), 497.1(100), 435.2(4), 339.3(6) |
| 453 | 22.29 | 702.5 | | MS2[702.5@35]: 684.3(100), 640.4(2), 627.3(79), 601.2(8), 443.1(3) | 460 | 22.03 | 969.5 | MS2[969.7@35]: 969.2(3), 951.4(4), 926.7(2), 907.6(8), 833.5(2), 793.5(2), 514.3(2), 351.1(100), 289.1(3) |
| 454 | 22.47 | 999.5 | | MS2[999.4@35]: 981.4(65), 937.3(14), 823.4(71), 677.3(6), 647.4(10), 497.2(100), 478.9(9), 371.2(13), 351.0(7), 321.1(10) | 461 | 22.02 | 799.4 | MS2[799.2@35]: 725.1(22), 605.2(2), 549.2(2), 531.2(100), 399.3(4), 255.2(8), 254.5(2) |
| 462 | 23.02 | 1065.3 | | MS2[1065.2@35]: 1146.4(34), 902.7(100), 869.2(64) | 471 | 23.00 | 493.3 | MS2[692.2@35]: 549.2(100), 546.2(9), 531.1(18), 255.1(4) |
| 463 | 22.66 | 593.3 | | MS2[593.2@35]: 545.0(93), 525.3(100) | 472 | 22.39 | 695.3 | MS2[695.3@35]: 575.2(4), 549.2(100), 531.2(59), 255.2(6) |
| 464 | 22.71 | 285.2 | | MS2[285.2@35]: 285.1(100), 270.1(34), 253.3(5), 191.1(5) | 473 | 22.53 | 725.3 | MS2[725.2@35]: 605.1(2), 575.2(4), 563.3(2), 549.2(100), 531.2(97), 429.4(2), 255.2(5) |
| 465 | 22.81 | 468.9 | | MS2[469.0@35]: 468.1(100), 432.6(4), 425.0(6), 405.2(5), 392.9(15), 254.3(6) | 474 | 22.49 | 853.5 | ---- |
| 466 | 22.81 | 839.5 | | MS2[839.6@35]: 838.2(2), 821.4(6), 795.3(2), 671.3(3), 663.5(6), 661.6(5), 556.9(2), 351.1(100), 333.4(2) | 475 | 23.01 | 990.5 | MS2[990.5@35]: 972.4(2), 946.4(100), 742.0(2), 703.5(2), 588.1(3) |
| 467 | 22.87 | 309 | | MS2[309.0@35]: 265.0(100) | 476 | 23.04 | 969.7 | MS2[969.6@35]: 969.8(1), 952.4(6), 951.5(13), 926.6(1), 908.6(1), 907.8(4), 902.4(1), 890.5(1), 833.6(2), 794.8(1), 793.5(10), 631.7(1), 594.3(2), 352.1(6), 351.1(100), 334.2(1), 333.0(7) |
| 468 | 22.95 | 741.1 | | ---- | 477 | 23.06 | 853.6 | MS2[853.6@35]: 835.3(7), 774.2(2), 747.0(2), 677.5(7), 659.5(3), 351.1(100) |
| 469 | 22.88 | 969.6 | | MS2[969.5@35]: 951.3(4), 907.5(8), 835.6(2), 793.7(5), 569.2(4), 351.1(100) | 478 | 23.06 | 987.2 | MS2[987.2@35]: 798.1(100) |
| 470 | 22.76 | 841.6 | | MS2[841.6@35]: 842.3(1), 824.7(1), 823.4(9), 810.4(3), 796.8(2), 787.8(2), 781.4(1), 779.6(1), 707.4(1), 665.4(5), 660.5(1), 647.4(1), 351.2(100), 289.2(2) | 479 | 23.11 | 695.3 | ---- |
| 480 | 23.21 | 733.1 | | MS2[733.1@35]: 1001.2(1), 733.4(2), 732.5(2), 715.1(2), 598.7(3), 557.1(100), 556.5(2), 505.9(1), 489.4(1), 444.7(1), 367.0(1) | 489 | 23.42 | 809.6 | MS2[809.5@35]: 780.5(2), 779.4(100), 735.5(8), 717.5(16), 659.4(10), 629.5(20), 585.5(3), 553.4(2), 471.4(5) |
| 481 | 23.24 | 725.3 | | MS2[725.2@35]: 725.0(2), 575.2(3), 563.3(2), 549.3(100), 531.2(71), 417.2(5), 399.2(5), 255.1(11) | 490 | 23.51 | 837.5 | MS2[837.5@35]: 819.5(12), 775.5(2), 661.5(12), 351.1(100), 333.0(2), 289.0(2) |
| 482 | 23.00 | 692.3 | | MS2[692.2@35]: 549.2(100), 546.2(9), 531.1(18), 255.1(4) | 491 | 23.53 | 569.2 | MS2[569.2@35]: 569.5(2), 476.1(2), 475.1(16), 449.2(52), 443.0(3), 430.1(5), 429.2(87), 414.3(10), 335.2(4), 310.1(2), 309.1(100), 294.3(9), 189.0(2) |
| 483 | 23.55 | 511.1 | | MS2[511@35]: 431(100), 269(6) | 492 | 23.58 | 627.3 | MS2[627.2@35]: 580.3(100), 338.9(72) |
| 484 | 23.20 | 507.2 | | MS2[507.2@35]: 461.2(100) | 493 | 23.73 | 563.3/609 | MS2[839.6@35]: 839.3(2), 821.5(6), 663.4(4), 488.0(2), 351.2(100) |
| 485 | 23.33 | 839.4 | | MS2[839.5@35]: 821.3(10), 779.2(3), 777.5(2), 663.3(7), 352.4(4), 351.1(100), 289.3(2) | 494 | 23.78 | 521.4 | MS2[997.4@35]: 835.3(21), 821.1(2), 791.5(2), 577.3(100), 565.3(28), 549.4(2), 445.3(2), 282.8(2) |
| 486 | 23.36 | 255.2 | | MS2[255.2@35]: 255.1(100), 153.1(35), 135.1(93), 119.2(9), 91.1(2) | 495 | 23.73 | 839.6 | MS2[839.6@35]: 839.3(2), 821.5(6), 663.4(4), 488.0(2), 351.2(100) |
| 487 | 23.37 | 791.0 | | MS2[791.0@35]: 666.5(100), 653.4(41), 639.3(94), 573.1(41) | 496 | 23.82 | 853.5 | MS2[853.5@35]: 835.4(3), 823.4(100), 822.7(2), 809.3(2), 805.5(3), 791.4(2), 719.5(3), 677.4(7), 351.2(40) |
| 488 | 23.38 | 255.2 | | MS2[255.2@35]: 255.1(100), 153.1(35), 135.1(93), 119.2(9), 91.1(2) | 497 | 23.56 | 999.5 | MS2[999.5@35]: 981.4(21), 919.5(3), 677.4(5), 497.2(100), 405.1(11), 339.2(9), 321.1(6) |
| 498 | 23.91 | 791.0 | | MS2[791.2@35]: 792.0(97), 772.3(27), 760.4(50), 755.3(84), 745.3(33), 728.2(47), 701.2(36), 634.6(56), 587.6(21), 550.5(34), 533.1(100), 477.1(37), 417.2(65),312.2(70) | 505 | 24.18 | 853.5 | MS2[853.5@35]: 836.1(2), 835.4(14), 831.7(5), 809.3(4), 792.5(5), 791.3(9), 765.1(2), 678.3(4), 677.5(16), 660.4(2), 659.6(4), 571.2(4), 501.3(10), 351.1(100) |
| 499 | 23.94 | 1117.8 | | MS2[1118@35]: 497(100), 437(2), 339(5) | 506 | 24.06 | 955.6 | MS2[956@35]: 981(2), 957(2), 939(5), 937(2), 927(2), 893(2), 779(3), 762(3), 352(9), 351(100), 350(2) |
| 500 | 23.95 | 853.5 | | MS2[853.5@35]: 853.8(8), 835.5(5), 809.1(4), 793.5(5), 791.3(7), 773.5(2), 709.6(4), 678.3(2), 677.4(22), 660.7(4), 571.5(6), 501.4(5), 351.1(100), 289.0(2) | 507 | 24.04 | 895.2 | MS2[895.2@35]: 877.2(2), 847.0(15), 753.0(3), 699.1(100), 531.2(32), 369.2(2) |
| 501 | 24.08 | 567.3 | | MS2[567.2@35]: 568.2(10), 567.2(26), 553.2(19), 552.1(100), 521.3(7), 473.2(3) | 508 | 24.25 | 858.8 ^a^ | MS2[858.8@35]: 841.0(48), 814.2(12), 712.2(34), 711.1(14), 697.1(13), 695.5(24), 694.0(100), 678.5(11), 586.2(8), 514.8(7), 500.8(6), 418.1(9), 398.9(17), 329.1(12) |
| 502 | 23.99 | 253.4 | | MS2[253.4@35]: 254.4(12), 253.4(100) | 509 | 24.22 | 716.4 | MS2[716.4@35]: 698.3(100), 672.6(2), 654.4(3), 641.4(82), 615.6(8), 514.3(2), 457.3(3) |
| 503 | 24.26 | 493.4 ^a^ | | MS2[493.2@35]: 447.2(100) | 510 | 23.94 | 859.4 | MS2[859.5@35]: 858.9(5), 837.3(9), 822.0(6), 813.3(100), 795.9(7), 795.2(4), 778.6(15), 769.0(6), 756.6(6), 698.6(4), 664.0(14), 662.2(5), 636.0(7), 608.3(4), 481.8(6) |
| 504 | 23.91 | 695.3 | | MS2[695.3@35]: 549.2(100), 531.2(58), 417.1(3), 399.5(2), 255.3(7) | 511 | 24.32 | 859.4 | MS2[859.5@35]: 813.4(100), 795.3(11), 724.1(4), 529.0(5) |
| 512 | 24.26 | 1115.7 | | MS2[1116@35]: 1098(10), 497(100), 339(7), 321(7) | 519 | 24.74 | 449.1 | MS2[449.0@35]: 402.9(100), 241.3(8) |
| 513 | 24.31 | 633 ^a^ | | MS2[633.0@35]: 633.2(21), 632.2(55), 618.2(26), 614.5(39), 603.1(22), 589.2(48), 588.3(100), 586.3(16), 480.4(42), 479.1(12), 441.3(58), 438.1(19), 398.3(25), 353.0(42), 330.0(30), 328.2(23) | 520 | 24.41 | 571.3 | MS2[521.5@35]: 522.4(30), 521.4(100), 401.4(4), 359.4(9), 358.3(6), 357.4(7) |
| 514 | 24.33 | 725.3 | | MS2[725.3@35]: 575.1(3), 549.2(100), 531.3(81), 417.3(6), 399.3(11),  255.2(11) | 521 | 24.61 | 791.0 | MS2[791.1@35]: 657.8(100), 546.3(22), 376.2(34) |
| 515 | 24.36 | 623.1 | | MS2[623.1@35]: 605.0(10), 577.8(28), 461.2(100), 442.3(18), 411.2(13), 289.4(11), 234.9(27), 221.3(13), 177.1(15) | 522 | 24.52 | 669.2 | MS2[669.2@35]: 549.2(26), 531.2(100), 399.3(2), 255.2(10) |
| 516 | 24.39 | 871.5 | | MS2[871.5@35]: 906.5(2), 870.1(2), 853.2(3), 811.5(2), 709.3(2), 605.4(5), 604.2(7), 603.2(100), 590.2(3), 589.2(51), 559.3(7), 504.2(2), 491.2(2), 427.5(3), 421.2(2), 352.3(4), 351.1(59), 271.0(3) | 523 | 24.53 | 853.5 | MS2[853.5@35]: 836.1(2), 835.4(14), 831.7(5), 809.3(4), 792.5(5), 791.3(9), 765.1(2), 678.3(4), 677.5(16), 660.4(2), 659.6(4), 615.3(2), 579.1(2), 571.2(4), 501.3(10), 351.1(100) |
| 517 | 24.4 | 283.1 | | MS2[283.3@35]: 284.2(7), 283.1(100), 269.3(2), 268.2(51) | 524 | 24.05 | 697.3 | MS2[697.3@35]: 731.3(2), 549.2(77), 532.1(3), 531.2(100), 513.2(5), 472.7(3), 417.1(3), 399.2(9), 255.2(9) |
| 518 | 24.43 | 991.4 | | MS2[991.4@35]: 960.2(100), 766.3(78) | 525 | 24.15 | 773.4 | MS2[773.3@35]: 773.0(4), 755.2(25), 713.1(16), 711.1(11), 631.1(4), 549.2(100), 548.4(2), 531.2(69), 517.3(4), 417.2(6), 399.1(8), 255.1(13) |
| 526 | 23.78 | 997.4 | | MS2[997.4@35]: 835.3(21), 821.1(2), 791.5(2), 577.3(100), 565.3(28), 549.4(2), 445.3(2), 282.8(2) | 536 | 24.74 | 449.1 ^a^ | MS2[449.0@35]: 402.9(100), 241.3(8) |
| 527 | 24.33 | 207.3 | | MS2[207.3@35]: 208.1(4), 207.2(100), 179.2(7) | 537 | 24.74 | 971.0 | MS2[971.0@35]: 953.3(100), 827.7(15), 779.1(13), 356.5(17) |
| 528 | 24.33 | 985.5 | | MS2[983.5@35]: 965.9(2), 965.3(23), 955.1(4), 947.3(2), 939.6(4), 921.3(18), 903.2(6), 863.2(2), 859.2(3), 849.4(3), 703.2(2), 661.3(2), 643.6(2), 553.3(8), 537.6(2), 497.2(100), 496.5(5), 479.3(3), 435.3(3), 424.2(3), 339.1(16), 321.1(3) | 538 | 24.76 | 507.3 | MS2[507.3@35]: 461.2(100), 460.0(1), 265.0(1), 163.1(1) |
| 529 | 24.41 | 521.4 | | MS2[521.5@35]: 522.4(30), 521.4(100), 401.4(4), 359.4(9), 358.3(6), 357.4(7) | 539 | 24.79 | 921.2 | MS2[921.2@35]: 902.9(5), 873.1(15), 725.2(100), 724.4(3), 549.3(3), 531.2(17) |
| 530 | 24.57 | 755.3 | | MS2[755.2@35]: 549.2(65), 531.2(100), 429.1(2), 255.1(4), 223.2(2) | 540 | 24.83 | 893.4 | MS2[893.3@35]: 549.2(100), 531.3(13), 417.1(3), 361.2(2) |
| 531 | 24.62 | 475.2 | | MS2[475.2@35]: 299.1(100), 284.3(16) | 541 | 24.92 | 632.8 | MS2[632.8@35]: 690.2(27), 632.7(49), 564.8(20), 526.5(20), 382.2(100) |
| 532 | 24.65 | 1101.7 | | MS2[1102@35]: 1083(21), 1040(13), 779(2), 497(100), 339(24), 321(3) | 542 | 24.84 | 837.4 | MS2[837.5@35]: 819.2(8), 818.3(4), 808.3(3), 793.4(4), 775.5(7), 761.3(2), 703.4(3), 661.7(6), 555.2(5), 351.1(100), 333.1(2) |
| 535 | 24.73 | 935.6 | | MS2[935.6@35]: 936.5(79), 935.6(100), 918.5(17), 917.5(53), 889.4(2), 774.6(16), 773.5(87), 756.4(2), 755.6(17), 727.4(3), 611.6(3), 565.5(2) | 543 | 24.90 | 1011.4 | MS2[1011.4@35]: 993.3(35), 689.6(23), 688.8(13), 671.6(22), 497.1(100), 435.1(9) |
| 544 | 24.98 | 311.2 | | MS2[311.2@35]: 293.1(100) | 546 | 24.99 | 811.3 | MS2[811.4@35]: 781.2(5), 549.2(100), 548.5(1), 531.3(32), 512.9(1), 501.3(1), 417.1(5), 399.2(3), 279.1(1),  255.1(4) |
| 545 | 25.01 | 871.5 | | MS2[871.5@35]: 853.3(4), 695.3(3), 605.2(8), 604.3(3), 603.2(100), 590.3(2), 589.3(54), 560.1(4), 559.2(6), 541.2(2), 505.2(3), 504.2(8), 491.2(2), 427.4(2), 351.1(60), 307.1(2) | 547 | 25.07 | 507.3 | MS2[507.3@35]: 507.4(1), 461.2(100), 460.2(1), 325.0(1), 264.9(1), 163.1(2) |
| 546 | 24.99 | 811.3 | | MS2[811.4@35]: 781.2(5), 780.2(1), 549.2(100), 548.5(1), 531.3(32), 530.3(1), 512.9(1), 501.3(1), 417.1(5), 399.2(3), 279.1(1), 255.1(4) | 549 | 25.07 | 521.4 | MS2[521.4@35]: 522.4(16), 521.4(100), 401.3(5), 360.4(4), 359.4(76), 358.4(12), 357.5(8), 340.4(2), 339.4(3) |
| 547 | 25.07 | 507.3 | | MS2[507.3@35]: 507.4(1), 461.2(100), 460.2(1), 325.0(1), 264.9(1), 163.1(2) | 550 | 25.10 | 985.6 | MS2[985.5@35]: 985.3(58), 823.3(37), 691.1(100) |
| 548 | 25.04 | 669.3 | | MS2[669.3@35]: 549.2(24), 531.2(100), 417.1(5), 399.2(7), 255.2(11),  254.1(2) | 551 | 25.15 | 692.3 | MS2[692.3@35]: 549.2(100), 531.3(27), 255.3(3) |
| 544 | 24.98 | 311.2 | | MS2[311.2@35]: 293.1(100) | 552 | 24.96 | 299.2 | MS2[299.2@35]: 299.2(7), 255.2(100) |
| 545 | 25.01 | 871.5 | | MS2[871.5@35]: 853.3(4), 695.3(3), 605.2(8), 604.3(3), 603.2(100), 590.3(2), 589.3(54), 560.1(4), 559.2(6), 541.2(2), 505.2(3), 504.2(8), 491.2(2), 427.4(2), 351.1(60), 307.1(2) | 553 | 25.2 | 853.5 | MS2[853.5@35]: 1244.4(3), 835.8(4), 835.2(14), 809.9(2), 808.7(5), 807.5(2), 792.6(2), 791.2(9), 739.3(2), 695.6(1), 678.7(6), 677.4(26), 659.1(1), 571.3(1), 502.6(1), 501.5(5), 471.3(2), 453.5(2), 351.7(1), 351.1(100), 289.1(2) |
| 554 | 25.33 | 983.5 | | MS2[983.6@35]: 965.5(28), 921.5(14), 903.8(6), 661.6(8), 553.3(2), 497.1(100), 479.0(10), 453.4(4), 451.1(2), 435.2(12), 338.4(9), 321.2(18) | 560 | 25.39 | 871.4 | MS2[871.5@35]: 871.3(2), 853.3(4), 852.3(2), 695.4(2), 665.1(2), 605.3(3), 603.2(100), 589.3(23), 559.3(4), 545.4(2), 504.5(3), 390.6(2), 351.2(50), 289.0(2) |
| 555 | 25.27 | 823.5 | | MS2[823.6@35]: 805.3(5), 761.4(6), 648.5(2), 647.2(4), 351.1(100), 289.2(3), 245.1(3) | 561 | 25.43 | 459.1 | MS2[459.1@35]: 283.1(100), 268.3(3), 175.0(11) |
| 556 | 25.30 | 711.3 | | MS2[711.3@35]: 591.0(2), 583.2(4), 565.3(100), 547.3(27), 451.1(2), 433.2(5), 415.3(13), 307.3(2), 271.2(12) | 562 | 25.44 | 561.2 | MS2[561.1@35]: 515.4(100), 514.3(20), 503.1(22), 445.0(40), 362.7(24) |
| 557 | 25.39 | 837.5 | | MS2[871.6@35]: 870.9(2), 854.2(4), 853.0(3), 807.7(2), 759.6(5), 735.2(2), 726.5(2), 605.3(4), 604.4(4), 603.2(100), 590.2(4), 589.2(59), 559.2(3), 519.0(2), 505.3(4), 504.2(5), 384.5(3), 351.1(37), 289.2(2) | 563 | 25.48 | 741.3 | MS2[741.3@35]: 613.1(2), 591.3(3), 579.2(9), 565.3(100), 564.7(2), 561.0(2), 547.2(76), 531.1(4), 529.3(3), 433.2(6), 415.3(14), 271.2(16), 270.2(3) |
| 558 | 25.41 | 823.5 | | MS2[823.5@35]: 806.1(2), 805.2(9), 761.3(4), 351.1(100), 333.3(2) | 564 | 25.50 | 695.3 | MS2[695.3@35]: 575.1(3), 549.2(100), 531.2(62), 255.2(3) |
| 559 | 25.36 | 855.4 | | MS2[855.5@35]: 873.2(3), 837.4(12), 811.1(2), 793.5(8), 721.5(3), 679.3(7), 351.1(100) | 565 | 25.50 | 865.5 | MS2[865.5@35]: 848.1(2), 847.3(10), 820.8(2), 803.5(2), 759.3(5), 689.3(19), 627.4(2), 599.0(2), 583.5(2), 568.9(2), 469.3(3), 351.1(100), 332.6(1), 289.2(2) |
| 566 | 25.72 | 1099.6 | | MS2[1099.6@35]: 497.2(100) | 567 | 25.55 | 999.7 | MS2[999.5@35]: 980.9(10), 955.5(16), 937.4(26), 823.1(7), 677.4(26), 659.2(6), 497.1(100), 479.1(9), 409.3(15), 320.7(18) |
| 568 | 25.66 | 985.5 | | MS2[985.5@35]: 823.5(100) | 575 | 25.93 | 725.3 | MS2[725.2@35]: 549.2(37), 531.2(100), 399.3(5), 255.2(5) |
| 569 | 25.67 | 955.6 | | MS2[956@35]: 938(3), 937(9), 912(2), 893(4), 867(3), 780(4), 761(2), 743(2), 597(2), 585(2), 352(2), 351(100),  289(2) | 576 | 25.80 | 975.3 | ---- |
| 570 | 25.68 | 729.3 | | MS2[729.3@35]: 685.2(100), 549.2(46), 531.2(72), 473.6(2), 417.2(8), 399.4(9), 255.2(12), 254.2(2) | 577 | 25.96 | 1149 | MS2[1148.8@35]: 1130.4(44), 987.2(62), 934.2(100), 933.3(52) |
| 571 | 25.74 | 591.3 | | MS2[591.3@35]: 591.2(10), 549.3(100), 531.2(24), 459.1(30), 429.3(5), 417.2(5), 399.1(10), 297.2(16), 255.2(57) | 578 | 25.98 | 695.3 | MS2[695.3@35]: 549.2(81), 531.2(100), 417.2(3), 399.2(5), 255.3(7) |
| 572 | 25.76 | 825.6 | | MS2[826@35]: 807(9), 649(4), 352(4), 351(100) | 579 | 25.91 | 853.6 | MS2[853.6@35]: 835.3(6), 810.2(2), 809.3(5), 791.5(5), 678.7(2), 677.4(22), 659.6(4), 615.3(1), 587.4(1), 502.1(1), 501.4(9), 457.4(1), 439.2(3), 371.3(3), 352.1(2), 351.1(100), 333.1(3), 288.6(1) |
| 573 | 25.76 | 697.2 | | MS2[697.2@35]: 660.3(43), 640.5(28), 550.3(100), 549.2(26), 533.2(65), 532.3(30), 499.2(33), 493.3(43) | 580 | 25.83 | 341.3 | MS2[341.2@35]: 323.1(100), 279.1(2) |
| 574 | 25.80 | 835.4 | | MS2[835.4@35]: 869.0(2), 834.3(1), 817.3(13), 794.7(1), 791.2(2), 788.1(1), 775.5(1), 773.4(4), 711.6(1), 701.4(1), 670.0(1), 659.3(10), 641.4(7), 483.3(2), 351.1(100), 333.2(2), 289.2(3), 276.7(2) | 581 | 25.99 | 985.5 | MS2[985.5@35]: 940.9(20), 923.6(6), 823.4(100), 791.2(12), 485.3(11) |
| 582 | 25.96 | 998.6 | | MS2[998.8@35]: 980.3(100) | 593 | 26.49 | 825.0 | ---- |
| 583 | 25.97 | 869.1 | | ---- | 594 | 26.64 | 927.0 | ---- |
| 584 | 25.83 | 371.4 | | MS2[371.4@35]: 372.3(19), 371.3(100), 370.7(3), 303.3(2), 285.3(2) | 595 | 26.67 | 271.3 | MS2[271.2@35]: 271.2(95), 177.2(22) 168.6(2), 165.3(3), 151.1(100), 119.3(3), 107.2(2), 93.4(4) |
| 585 | 26.01 | 385.3 | | MS2[385.3@35]: 386.2(15), 385.2(100), 371.1(2), 370.2(42), 367.2(2), 313.2(2), 298.2(2), 270.2(2) | 596 | 26.99 | 269.3 | MS2[269.3@35]: 270.2(4), 269.2(100), 237.4(8), 175.3(7), 149.1(3) |
| 586 | 26.05 | 1115.7 | | MS2[1115.7@35]: 1071.0(97), 953.4(100) | 597 | 26.76 | 1100.8 | MS2[1100.9@35]: 644.6(100) |
| 587 | 26.15 | 745.2 | | MS2[745.2@35]: 677.3(34), 676.4(56), 663.3(37), 608.4(100) | 599 | 26.82 | 895.5 | MS2[896@35]: 878(4), 877(10), 851(2), 835(2), 834(2), 833(8), 762(2), 720(4), 719(12), 702(2), 660(3), 352(10), 351(100), 350(2), 333(2) |
| 588 | 26.19 | 597.4 | | MS2[597.4@35]: 597.3(100), 596.6(3), 445.3(69), 385.2(5), 343.2(11), 301.2(3), 283.1(19), 241.1(4), 223.2(2), 169.1(2) | 600 | 26.50 | 821.4 | MS2[821.5@35]: 803.4(6), 791.3(66), 397.5(5), 351.1(100), 258.9(9) |
| 589 | 26.22 | 1032.9 | | MS2[1033.0@35]: 974.7(100) | 601 | 25.46 | 581.3 | MS2[581.3@35]: 537.3(100), 536.6(2), 519.2(2), 509.3(21) |
| 590 | 26.17 | 823.6 | | MS2[823.6@35]: 805.2(5), 761.4(2), 647.6(2), 351.1(100), 350.3(3), 333.2(4), 289.1(4) | 602 | 25.94 | 999.7 | ---- |
| 591 | 26.30 | 1017.1 | | MS2[1116.6@35]: 735.8(100) | 603 | 26.09 | 1117.6 | MS2[999.5@35]: 981.2(9), 909.4(8), 880.0(6), 879.2(100), 860.9(5), 837.4(58), 805.3(5), 642.9(8), 496.7(8), 485.5(7), 351.2(24) |
| 592 | 26.61 | 1115.6 | | MS2[1115.7@35]: 1115.5(51), 1097.4(100), 1053.2(17), 821.9(20), 807.4(25), 439.6(37) | 604 | 26.17 | 1085.6 | MS2[1085.5@35]: 1067.4(6), 1023.3(3), 585.2(4), 497.1(100), 450.7(4), 435.2(3), 339.3(17), 338.5(2) |
| 605 | 26.30 | 939.6 | | MS2[939.6@35]: 922.5(3), 921.4(7), 877.5(3), 874.3(2), 763.1(5), 352.1(4), 351.1(100), 333.1(2), 289.1(2) | 612 | 26.88 | 895.4 | MS2[895.5@35]: 877.3(5), 835.4(4), 833.2(7), 719.2(14), 661.1(3), 504.1(2), 483.5(5), 352.0(3), 351.1(100),  289.0(4) |
| 606 | 26.31 | 969.5 | | MS2[969.6@35]: 952.8(4), 951.5(75), 925.6(5), 908.4(7), 907.5(100), 823.4(11), 761.5(5), 661.4(2), 644.4(4), 643.4(96), 625.4(13), 599.5(20), 581.3(4), 553.5(89), 552.9(4), 509.5(6), 486.5(3), 485.5(92), 441.6(8), 423.4(9), 407.5(3), 355.5(15) | 613 | 26.84 | 569.2 | MS2[569.3@35]: 568.3(2), 523.0(24), 522.3(4), 493.0(100), 374.9(6), 345.2(3) |
| 607 | 26.35 | 837.5 | | MS2[837.5@35]: 819.4(11), 775.7(7), 661.5(5), 643.1(5), 351.1(100), 350.4(2), 333.3(2), 289.1(3) | 614 | 26.93 | 661.3 | MS2[661.4@35]: 643.2(100), 631.2(28), 613.1(6), 539.3(13), 521.2(17), 509.2(8), 495.2(2), 493.2(2), 343.0(4), 265.3(2), 223.2(2), 199.2(8) |
| 608 | 26.52 | 782.8 | | ---- | 615 | 26.90 | 833.5 | MS2[833.5@35]: 833.0(2), 815.3(13), 814.3(2), 811.0(2), 804.2(3), 789.4(5), 771.5(8), 752.0(2), 726.8(2), 657.3(22), 481.5(4), 351.1(100), 350.5(3), 263.0(2) |
| 609 | 26.58 | 985.0 | | MS2[985.0@35]: 985.2(100) | 616 | 27.00 | 1129.7 | MS2[1129.6@35]: 967.4(100), 949.2(22), 931.4(2), 905.3(4), 645.6(2), 497.1(10), 339.3(3) |
| 610 | 25.96 | 998.6 | | MS2[998.8@35]: 980.3(100) | 617 | 27.03 | 791.0 | ---- |
| 618 | 26.80 | 953.6 | | MS2[953.6@35]: 936.3(8), 935.3(11), 910.5(2), 892.6(5), 891.4(17), 777.4(5), 352.2(7), 351.1(100), 289.2(5) | 624 | 27.26 | 997.6 | MS2[997.6@35]: 821.2(100), 570.9(90) |
| 619 | 26.99 | 839.6 | | MS2[839.7@35]: 821.6(3), 795.5(8), 778.6(3), 777.5(18), 660.5(14), 659.5(85), 615.6(7), 584.6(2), 583.5(4), 571.6(2), 502.5(14), 501.5(100), 483.4(5), 457.5(8), 439.5(3), 371.4(6), 323.5(2) | 625 | 27.29 | 583.2 | MS2[629.1@35]: 629.3(100), 585.7(38), 493.0(38), 449.2(40), 349.9(66), 298.7(80) |
| 620 | 27.16 | 989.1 | | MS2[989.4@35]: 827.4(100) | 626 | 27.36 | 983.7 | MS2[983.5@35]: 863.3(2), 821.4(100), 803.3(15), 645.6(3), 351.1(7) |
| 621 | 27.31 | 985.6 | | MS2[985.4@35]: 985.5(6), 967.5(37), 941.5(6), 923.4(60), 909.6(2), 879.3(2), 851.2(4), 839.5(4), 715.2(5), 659.4(54), 641.3(4), 612.4(3), 569.5(5), 543.3(2), 529.4(2), 501.3(100), 483.2(10), 457.1(3), 423.2(2), 371.1(2), 323.6(3) | 627 | 27.39 | 697.3 | MS2[697.3@35]: 603.4(2), 576.5(2), 551.1(2), 550.3(5), 549.2(83), 532.2(4), 531.2(100), 429.1(3), 399.2(2), 255.1(9) |
| 622 | 27.06 | 837.5 | | MS2[837.5@35]: 871.8(2), 820.2(3), 819.2(5), 793.2(4), 775.4(15), 744.4(3), 661.5(21), 643.4(13), 485.2(8), 355.4(2), 351.1(100), 288.9(2) | 628 | 27.13 | 1119.2 | MS2[1119.3@35]: 947.8(100) |
| 623 | 27.05 | 999.6 | | MS2[999.4@35]: 981.5(25), 963.4(27), 875.4(16), 677.6(5), 497.2(100), 496.4(7), 435.5(17), 339.1(6) | 629 | 27.49 | 1057.2 | MS2[1057.0@35]: 613.1(100) |
|  |  |  | |  |  |  |  |  |
| 630 | 27.25 | 661.3 | | MS2[661.4@35]: 643.2(23), 631.3(9), 625.5(2), 613.2(2), 539.3(39), 521.2(5), 509.0(3), 495.3(100), 491.6(2), 357.1(2), 343.1(5), 283.0(2), 199.2(15) | 637 | 27.59 | 837.6 | MS2[837.6@35]: 819.3(19), 801.6(2), 794.3(2), 793.3(4), 775.4(21), 661.5(7), 643.4(8), 485.3(8), 351.1(100), 333.1(2), 307.0(4), 289.0(3), 276.4(2) |
| 631 | 27.44 | 939.6 | | MS2[939.6@35]: 922.4(7), 921.4(7), 878.3(3), 877.4(10), 769.2(4), 764.6(3), 763.3(6), 617.5(5), 352.2(3), 351.1(100), 289.1(2) | 638 | 27.61 | 995.5 | MS2[995.4@35]: 977.6(2), 959.6(3), 951.5(4), 672.6(4), 629.0(2), 542.4(2), 497.2(100), 479.1(4), 339.1(4) |
| 632 | 27.51 | 839.3 | | MS2[839.5@35]: 821.3(12), 795.3(3), 777.5(4), 763.8(2), 663.5(18), 645.1(2), 351.2(100) | 639 | 27.67 | 1131.7 | MS2[1131.6@35]: 1113.5(24), 1071.4(8), 1013.8(6), 997.3(10), 969.4(100), 955.5(3), 951.7(7), 907.5(12), 807.8(11), 781.0(6), 585.3(6), 497.0(39), 479.3(3), 338.8(3) |
| 633 | 27.49 | 971.6 | | ---- | 640 | 27.72 | 735.3 | MS2[735.2@35]: 735.2(11), 717.0(3), 688.3(2), 672.2(4), 666.7(2), 585.2(5), 539.0(23), 417.1(100), 259.0(9), 251.4(7) |
| 634 | 27.46 | 283.3 | | MS2[283.3@35]: 284.2(15), 283.2(100), 268.2(11) | 641 | 27.76 | 757.8 | MS2[757.6@35]: 676.5(2), 550.3(20), 549.2(100), 350.8(2) |
| 635 | 27.79 | 283.2 | | MS2[283.2@35]: 284.2(10), 283.2(67), 282.6(4), 281.9(2), 268.2(15), 239.2(7), 177.1(100), 176.3(2), 133.2(26), 131.1(3) | 642 | 27.66 | 695.4 | MS2[695.2@35]: 575.0(3), 549.2(100), 531.2(54), 399.4(4), 255.3(5) |
| 636 | 27.59 | 1117.5 | | MS2[1117.7@35]: 1117.5(62), 1099.5(100), 1087.3(3), 1055.6(2), 955.5(27), 937.5(15), 919.6(9), 901.6(6), 893.5(4), 847.5(15), 731.6(14), 713.3(2), 685.5(11), 617.4(2), 569.5(3), 455.7(3) | 643 | 27.74 | 895.4 | MS2[895.4@35]: 950.1(2), 877.4(9), 851.1(4), 837.4(3), 833.6(10), 789.1(2), 773.4(10), 764.2(2), 760.4(2), 731.7(2), 719.3(6), 659.4(5), 599.2(2), 552.9(2), 430.1(2), 352.1(3), 351.1(100), 289.1(6) |
| 644 | 27.90 | 725.3 | | MS2[725.3@35]: 575.2(4), 549.2(47), 531.2(100), 399.3(4), 255.2(7) | 653 | 27.94 | 997.6 | MS2[997.5@35]: 951.3(100), 821.5(76), 350.8(48) |
| 645 | 27.77 | 811.3 | | ---- | 654 | 28.00 | 779.6 | ---- |
| 646 | 27.97 | 537.1 | | MS2[537.3@35]: 538.1(7), 537.2(30), 536.5(2), 523.2(14), 522.2(100), 521.4(2), 402.2(9), 401.2(100), 400.5(7) | 655 | 28.05 | 601.2 | MS2[601.2@35]: 601.2(7), 539.1(30), 499.4(84), 498.8(3), 457.3(100), 439.3(20) |
| 647 | 27.83 | 1117.7 | | MS2[1117.7@35]: 1117.0(26), 955.5(100), 923.1(21) | 656 | 28.11 | 849.5 | MS2[849.5@35]: 850.1(3), 832.5(3), 831.4(5), 805.3(3), 789.1(2), 787.4(7), 778.4(3), 743.5(3), 691.0(2), 674.0(2), 673.2(7), 663.1(2), 629.4(3), 567.5(2), 520.4(2), 351.1(100) |
| 648 | 27.85 | 629.2 ^a^ | | MS2[629.2@35]: 585.1(39), 583.1(100), 582.5(12), 478.0(52), 429.3(48) | 657 | 28.11 | 361.2 | MS2[361.2@35]: 318.4(3), 289.3(100), 269.3(2) |
| 649 | 27.51 | 327.4 | | MS2[327.4@35]: 327.3(100), 309.3(4), 291.3(5), 229.4(6), 171.4(4) | 658 | 28.13 | 1099.6 | MS2[1099.5@35]: 1141.5(13), 1126.1(13), 1081.1(8), 978.4(4), 497.1(100), 338.9(18) |
| 650 | 27.82 | 215.2 | | MS2[215.2@35]: 215.3(100), 197.2(21), 153.2(10) | 659 | 28.17 | 692.3 | MS2[692.3@35]: 549.2(100), 531.2(29), 255.2(3) |
| 651 | 27.86 | 369.3 | | MS2[369.3@35]: 370.2(17), 369.1(100), 368.0(4), 354.1(2), 351.2(4), 339.1(2), 337.2(10), 325.3(2), 299.2(40), 284.1(8), 282.7(2), 191.1(4), 177.1(3) | 660 | 28.18 | 981.6 | MS2[982@35]: 982(2), 963(3), 847(2), 498(4), 497(100), 479(2), 381(2), 339(18), 321(5) |
| 652 | 27.87 | 327.4 | | MS2[327.4@35]: 327.3(100), 309.4(8), 291.4(8), 229.4(5), 171.3(9) | 661 | 28.03 | 791.0 | ---- |
|  |  |  | |  |  |  |  |  |
| 662 | 28.04 | 1025.6 | | MS2[1025.5@35]: 1007.1(9), 963.7(12), 947.2(10), 891.8(4), 703.7(3), 599.1(3), 510.2(3), 497.1(100), 339.0(13), 321.4(4), 320.7(4) | 669 | 28.65 | 697.5 | MS2[697.4@35]: 653.3(8), 565.3(100) |
| 663 | 28.20 | 371.4 | | MS2[371.3@35]: 372.3(15), 371.2(100), 370.3(4), 327.3(2), 303.4(16), 285.3(5) | 670 | 29.98 | 697.4 | MS2[697.4@35]: 653.3(7), 565.3(100) |
| 664 | 28.46 | 969.8 | | MS2[969.8@35]: 952.4(11), 951.5(14), 925.7(2), 908.6(6), 907.6(21), 835.2(2), 662.4(3), 644.6(7), 643.4(47), 599.4(7), 554.3(4), 553.5(21), 509.6(3), 486.4(20), 485.5(100), 441.4(7), 423.7(2), 356.5(2), 355.6(10 | 671 | 28.49 | 1030.4 | MS2[1030.4@35]: 880.5(61), 874.6(56), 869.4(100), 762.5(38), 696.6(85), 403.2(68) |
| 665 | 28.06 | 1030.7 | | MS2[1030.7@35]: 1030.1(62), 578.3(100) | 672 | 28.57 | 269.3 | MS2[269.3@35]: 270.2(16), 269.3(100), 251.2(1) |
| 666 | 28.45 | 853.5 | | MS2[853.5@35]: 835.9(2), 835.3(13), 809.3(9), 791.4(14), 677.5(6), 659.5(9), 501.4(13), 351.1(100), 333.1(3), 289.2(3) | 673 | 28.62 | 923.4 | MS2[923.4@35]: 921.9(2), 879.4(100), 837.4(78), 762.3(2) |
| 667 | 28.18 | 837.6 | | MS2[837.6@35]: 819.7(6), 775.5(16), 661.2(6), 643.4(3), 642.8(2), 631.4(2), 485.4(3), 351.9(4), 351.1(100), 333.3(2), 275.8(2) | 674 | 28.74 | 791.0 | MS2[791.1@35]: 762.2(21), 702.3(16), 662.2(29), 650.3(34), 627.6(32), 562.8(100) |
| 668 | 28.37 | 695.3 | | MS2[695.3@35]: 563.0(12), 554.5(4), 549.2(100), 531.2(5), 479.1(5), 296.9(4) | 675 | 28.77 | 997.5 | MS2[997.5@35]: 979.4(30), 675.5(26), 497.1(100), 435.5(24) |
|  |  |  | |  |  |  |  |  |
|  |  |  | |  |  |  |  |  |
| 676 | 28.87 | 969.7 | | MS2[969.7@35]: 952.6(5), 951.5(27), 907.7(7), 837.5(3), 807.5(4), 794.7(2), 793.6(6), 733.5(6), 685.8(2), 351.2(100), 350.0(2), 289.1(4) | 682 | 28.96 | 954.9 | MS2[955.1@35]: 955.2(25), 937.8(26), 937.2(6), 909.7(36), 907.6(10), 895.4(36), 893.2(68), 839.0(15), 823.2(21), 815.5(12), 792.5(43), 775.6(53), 757.3(100), 731.7(25), 730.9(19), 729.0(21), 701.9(11), 685.4(96), 641.2(9), 587.4(15), 579.5(19), 569.7(13), 553.2(28), 538.1(19), 523.7(35), 487.3(19) |
| 677 | 28.74 | 1101.7 | | MS2[1101.6@35]: 1102.0(34), 1084.0(100), 1083.0(13), 1073.0(4), 1040.0(14), 956.0(9), 938.0(14), 895.0(11), 894.0(42), 869.0(3), 793.0(2), 776.0(16), 758.0(42), 750.0(3), 731.0(8), 686.0(59), 618.0(5), 524.0(9), 523.0(10), 481.0(8) | 683 | 29.02 | 629.0 | MS2[629.2@35]: 683.7(19), 601.9(56), 583.0(100) |
| 678 | 28.83 | 468.2 | | MS2[468.1@35]: 760.2(2), 655.1(2), 585.4(25), 584.4(100), 470.5(2), 469.3(6), 468.2(46), 459.0(3), 446.5(2), 445.8(2), 437.0(5), 410.6(4), 409.9(8), 404.9(2), 379.9(3), 351.2(10), 175.1(2) | 684 | 29.07 | 879.5 | MS2[879.5@35]: 862.2(4), 861.3(4), 819.6(5), 817.3(4), 775.3(2), 757.4(3), 703.3(14), 686.4(2), 643.7(5), 539.6(2), 352.0(3), 351.1(100), 333.0(2), 289.2(2) |
| 679 | 28.83 | 351.3 | | MS2[351.3@35]: 352.2(14), 351.2(100), 323.3(2), 307.2(1), 294.3(1), 281.1(1), 268.2(15), 241.3(2) | 685 | 29.07 | 823.7 | MS2[823.7@35]: 805.3(2), 779.5(2), 761.5(9), 661.4(3), 644.3(2), 643.4(39), 599.5(2), 567.3(2), 553.4(14), 486.5(10), 485.5(100), 441.5(3), 423.5(2), 355.4(9) |
| 680 | 29.07 | 302.9 ^a^ | | MS2[303.0@35]: 303.1(29), 302.0(27), 257.2(100) | 686 | 29.07 | 983.6 | MS2[983.6@35]: 965.0(78), 807.6(39), 496.9(100) |
| 681 | 28.91 | 911.0 | | MS2[910.9@35]: 893.1(100), 891.6(47), 888.1(70), 880.9(19), 879.4(18), 873.6(9), 872.6(14), 867.3(12), 865.0(21), 862.1(25), 856.2(6), 850.1(15), 847.1(28), 820.8(48), 812.3(16), 796.1(19), 779.1(13), 745.5(14), 687.8(14), 643.1(18), 569.4(27), 541.3(10), 503.1(22) | 695 | 29.45 | 969.6 | MS2[969.5@35]: 951.4(22), 906.1(1), 893.1(4), 833.3(2), 793.5(6), 776.5(3), 774.9(2), 674.5(3), 553.3(8), 351.2(100) |
| 687 | 29.07 | 823.5 | | MS2[823.5@35]: 805.3(8), 779.5(3), 761.5(3), 647.3(3), 471.5(2), 351.1(100), 333.1(2), 289.1(2) | 696 | 29.45 | 953.5 | MS2[953.6@35]: 935.6(13), 498.3(9), 497.2(100), 496.4(5), 435.1(4), 407.3(2), 340.0(2), 339.0(16), 321.1(2) |
| 688 | 29.16 | 491.4 | | MS2[491.3@35]: 491.2(52), 490.6(2), 476.2(28), 449.2(2), 431.2(100), 416.3(5), 371.3(22), 356.3(4), 339.2(2), 217.2(2) | 697 | 29.53 | 967.4 | MS2[967.4@35]: 847.4(4), 846.7(2), 805.4(100), 788.4(2), 787.4(24), 629.5(2), 351.2(13 |
| 689 | 29.16 | 369.3 | | MS2[369.2@35]: 370.3(16), 369.2(100), 354.2(24), 337.5(2), 284.4(2) | 698 | 29.58 | 997.5 | MS2[997.5@35]: 997.5(8), 979.7(12), 979.0(10), 935.6(7), 833.4(5), 497.3(100), 339.6(14), 339.0(19) |
| 690 | 29.24 | 830.8 | | MS2[830.8@35]: 821.4(2), 758.4(2), 748.0(2), 718.9(2), 710.4(2), 697.3(6), 695.2(100), 676.7(3), 591.0(2), 575.7(2), 549.2(17), 532.5(2), 531.1(9) | 699 | 29.73 | 856.6 | ----- |
| 691 | 29.3 | 1027.6 | | MS2[1027.6@35]: 1009.5(25), 983.8(3), 683.7(3), 497.1(100), 405.3(10), 339.0(7) | 700 | 29.53 | 823.7 | MS2[823.7@35]: 805.3(2), 779.5(2), 761.5(9), 661.4(3), 644.3(2), 643.4(39), 599.5(2), 567.3(2), 553.4(14), 486.5(10), 485.5(100), 441.5(3), 423.5(2), 356.6(2), 355.4(9) |
| 694 | 29.43 | 849.5 | | MS2[849.3@35]: 831.1(25), 827.1(7), 789.9(9), 789.2(4), 786.9(11), 780.1(5), 731.3(12), 725.2(7), 673.5(44), 602.2(7), 502.9(10), 479.5(6), 448.9(7), 351.1(100) | 701 | 29.48 | 1115.7 | MS2[1115.7@35]: 953.4(100) |
| 702 | 29.52 | 227.3 | | MS2[227.3@35]: 227.2(27), 209.1(5), 183.3(36), 165.3(100), 111.2(2) | 710 | 29.92 | 939.5 | MS2[939.5@35]: 822.5(2), 819.6(1), 778.3(1), 777.4(100), 759.1(1), 609.3(1), 569.3(1) |
| 703 | 29.95 | 547.1 ^a^ | | MS2[547.1@35]: 547.3(27), 501.4(34), 499.2(40), 484.3(100), 410.0(8), 409.3(78), 344.4(24), 336.1(19), 289.2(33), 270.1(64) | 711 | 29.95 | 329.4 | MS2[329.4@35]: 329.3(100), 229.3(4) |
| 704 | 30.10 | 327.4 | | MS2[327.3@35]: 327.2(48), 311.1(1), 309.2(100), 291.3(24), 273.2(8), 231.1(1), 213.3(10), 201.4(20), 200.8(2), 171.3(38), 139.2(1), 137.4(1), 136.4(1) | 712 | 30.20 | 329.4 | MS2[329.3@35]: 329.3(100), 311.3(2), 229.4(4), 211.3(2), 171.2(3) |
| 705 | 29.68 | 939.7 | | MS2[939.7@35]: 778.5(8), 777.4(100), 627.5(2) | 713 | 30.16 | 953.7 | MS2[953.7@35]: 935.3(6), 595.1(2), 498.2(6), 497.2(100), 435.3(4), 407.1(3), 340.2(2), 339.1(11), 321.2(10) |
| 706 | 29.69 | 677.4 | | MS2[723.0@35]: 677.9(59), 658.0(100) | 714 | 30.31 | 967.6 | MS2[967.6@35]: 847.2(4), 805.4(100), 788.5(4), 787.4(22), 629.4(2), 351.2(10) |
| 707 | 29.70 | 431.3 | | MS2[431.3@35]: 431.2(51), 389.4(2), 371.2(100), 370.6(3), 311.4(10) | 715 | 30.06 | 997.6 | MS2[997.5@35]: 979.6(6), 979.0(15), 497.2(100), 435.2(3), 406.7(6), 338.8(4), 337.2(6), 321.3(6) |
| 708 | 29.86 | 839.5 | | MS2[839.4@35]: 821.4(8), 803.3(5), 795.0(3), 777.5(10), 772.9(2), 663.5(10), 481.4(4), 410.5(2), 351.1(100) | 716 | 30.01 | 984.9 | MS2[984.8@35]: 903.7(100) |
| 709 | 29.88 | 1105.6 | | MS2[1105.6@35]: 1087.4(36), 1073.3(32), 1061.7(26), 1043.4(14), 929.3(34), 915.6(39), 803.3(23), 351.1(100 | 717 | 30.38 | 637.4 | MS2[637.3@35]: 637.1(4), 619.3(3), 605.3(2), 543.3(6), 542.5(2), 517.2(45), 501.2(4), 499.1(3), 497.2(82), 482.2(10), 429.1(23), 414.2(4), 403.1(3), 377.2(100), 362.2(18), 335.1(2), 309.2(26), 294.3(7) |
| 718 | 30.39 | 1091.5 | | MS2[1091.6@35]: 1073.4(10), 1055.4(3), 1042.5(2), 1037.0(4), 965.4(100), 915.4(22), 905.4(5), 903.0(2), 893.2(5), 845.4(8), 821.4(6), 687.2(5), 447.4(4), 351.0(38), 333.3(8) | 723 | 30.24 | 983.5 | MS2[983.5@35]: 965.6(15), 939.5(14), 922.6(2), 921.5(6), 903.2(3), 877.6(5), 821.7(6), 803.3(36), 762.4(3), 759.4(83), 697.2(3), 674.2(3), 673.2(5), 645.3(11), 627.4(17), 526.1(3), 514.1(5), 513.1(100), 497.1(33), 494.7(2), 479.3(4), 469.4(9), 435.5(5), 354.9(4), 351.0(2), 321.3(3) |
| 719 | 30.43 | 619.1 ^a^ | | MS2[619.2@35]: 572.7(100) | 724 | 30.19 | 837.6 | MS2[837.6@35]: 820.3(4), 819.3(11), 793.0(3), 775.3(5), 662.5(3), 661.4(20), 485.8(2), 352.1(3), 351.1(100) |
| 720 | 30.46 | 845.7 | | MS2[845.6@35]: 966.5(12), 965.5(46), 923.5(3), 845.6(2), 767.2(2), 726.2(20), 725.2(100), 697.8(3), 573.4(2), 550.4(2), 549.2(6), 531.2(4) | 725 | 30.33 | 975.4 ^a^ | MS2[975.4@35]: 931.4(39), 927.5(34), 917.6(42), 853.0(100), 829.2(37), 813.4(69), 491.4(44) |
| 721 | 30.49 | 868.9 | | ---- | 726 | 30.50 | 849.4 | MS2[849.5@35]: 831.4(5), 787.6(3), 773.0(2), 673.3(14), 497.0(2), 351.1(100), 333.0(2) |
| 722 | 30.69 | 965.6 | | MS2[965.5@35]: 966.7(2), 948.5(2), 947.3(3), 498.3(12), 497.2(100), 435.3(5), 340.2(2), 339.2(10), 321.0(8) | 727 | 30.60 | 953.6 | MS2[953.6@35]: 936.4(4), 935.3(8), 917.8(2), 891.4(4), 498.1(8), 497.1(100), 435.2(6), 339.1(16), 321.1(6) |
| 728 | 30.69 | 969.6 | | MS2[969.6@35]: 969.3(2), 849.1(2), 807.4(100), 789.4(23), 631.7(4), 351.1(11) | 737 | 31.07 | 837.5 | MS2[837.5@35]: 819.4(14), 793.4(4), 775.5(3), 661.4(7), 351.1(100), 333.0(3) |
| 729 | 30.80 | 735.4 | | MS2[665.4@35]: 665.4(22), 664.6(8), 619.1(100), 596.2(9), 583.2(5), 501.3(20), 499.4(12), 457.5(12), 255.2(7) | 738 | 31.12 | 998.9 | MS2[998.9@35]: 999.2(100) |
| 730 | 30.85 | 887.3 | | MS2[887.3@35]: 887.4(1), 857.4(1), 790.5(1), 736.3(5), 735.3(100), 569.3(1), 565.2(1) | 739 | 31.21 | 255.3 | MS2[255.2@35]: 256.2(3), 255.2(93), 153.1(32), 135.2(100), 119.3(10), 91.2(3) |
| 731 | 30.85 | 837.6 | | MS2[837.6@35]: 819.4(4), 775.6(4), 661.5(8), 351.1(100) | 740 | 31.26 | 967.6 | MS2[967.5@35]: 949.6(11), 906.4(4), 905.4(14), 887.3(3), 645.5(3), 627.4(4), 498.3(8), 497.2(100), 339.1(11), 321.1(11) |
| 732 | 30.86 | 665.4 | | MS2[665.4@35]: 665.4(22), 664.6(8), 619.1(100), 596.2(9), 583.2(5), 501.3(20), 499.4(12), 457.5(12), 255.2(7) | 741 | 31.36 | 637.3 | MS2[637.3@35]: 637.2(5), 543.2(8), 517.2(40), 497.2(100), 482.1(8), 429.2(18), 414.3(3), 377.2(66), 362.2(11), 309.2(10) |
| 733 | 30.89 | 851.5 | | MS2[851.5@35]: 833.4(14), 789.5(3), 761.9(2), 715.3(2), 692.1(3), 675.5(12), 551.0(2), 493.2(3), 351.1(100), 332.8(5) | 742 | 31.44 | 967.6 | MS2[967.5@35]: 950.5(2), 803.4(2), 645.3(4), 498.2(6), 497.2(100), 496.6(5), 435.0(4), 407.2(2), 339.0(11), 321.2(10) |
| 734 | 30.95 | 833.3 | | MS2[833.4@35]: 834.3(3), 815.2(4), 657.5(6), 351.1(100), 332.6(2) | 743 | 31.45 | 969.6 | MS2[969.5@35]: 951.3(12), 907.4(4), 497.1(100), 496.5(2), 453.2(2), 435.2(15), 339.1(20), 320.8(4) |
| 735 | 31.01 | 997.5 | | MS2[997.5@35]: 675.2(23), 497.4(100), 496.7(32), 479.0(23), 339.1(19) | 744 | 31.58 | 983.6 | ---- |
| 736 | 31.04 | 881.6 | | MS2[881.6@35]: 864.4(2), 863.3(15), 837.6(3), 745.2(2), 705.4(25), 687.2(2), 352.2(5), 351.1(100) | 745 | 31.30 | 868.9 | ---- |
| 746 | 31.43 | 267.3 | | MS2[267.2@35]: 268.2(2), 267.2(100), 252.3(7) | 754 | 31.46 | 693.2 | MS2[693.3@35]: 693.3(100), 663.3(30), 656.9(6), 636.4(11), 633.3(9), 556.4(13), 551.3(17), 538.6(10), 528.0(19), 365.1(30) |
| 747 | 31.52 | 695.3 | | MS2[695.3@35]: 578.3(2), 531.2(100), 513.1(2), 417.1(13), 399.4(6), 255.2(15) | 755 | 31.83 | 791.5 | MS2[791@35]: 773(9), 615(3), 351(100), 261(3) |
| 748 | 31.62 | 839.5 | | MS2[839.5@35]: 821.3(5), 777.5(2), 663.6(4), 352.2(5), 351.1(100), 289.2(3) | 756 | 31.94 | 311.3 | MS2[311.3@35]: 312.1(18), 311.2(100), 297.2(7), 296.2(48), 239.2(9) |
| 749 | 31.68 | 637.4 | | MS2[637.4@35]: 637.1(2), 636.2(3), 622.3(3), 621.2(7), 605.1(2), 543.1(3), 517.2(16), 497.2(3), 429.2(65), 414.2(6), 377.1(2), 335.3(3), 309.1(100), 308.5(2), 294.3(7) | 757 | 31.98 | 1011.6 | MS2[1011.5@35]: 993.6(8), 967.1(2), 949.2(4), 877.4(9), 535.5(7), 497.1(100), 435.2(6), 381.0(9), 339.0(30), 320.8(3) |
| 750 | 31.85 | 823.5 | | MS2[823.5@35]: 805.4(8), 777.5(2), 761.2(2), 647.5(2), 643.7(2), 629.6(2), 553.6(3), 352.1(2), 351.1(100), 289.1(2) | 758 | 32.13 | 619.0 | MS2[619.0@35]: 562.1(49), 507.9(100) |
| 751 | 31.88 | 295.2 | | MS2[295.3@35]: 295.1(17), 251.1(100), 250.4(2), 207.3(4), 147.1(56) | 759 | 32.13 | 617.4 | MS2[617.4@35]: 617.3(23), 485.2(34), 467.4(2), 453.1(2), 429.3(3), 365.2(4), 323.3(100), 322.6(2), 201.4(2) |
| 752 | 31.60 | 629.1 | | MS2[629.0@35]: 629.3(100), 628.2(77), 585.6(26), 583.2(28), 565.9(19), 547.1(21), 537.9(37) | 760 | 32.16 | 735.4 | MS2[735.4@35]: 735.2(100), 734.6(5), 689.4(5), 621.0(3), 613.2(8), 584.8(2), 569.4(5), 417.2(83), 399.0(2), 295.1(3) |
| 753 | 31.83 | 735.3 | | MS2[735.4@35]: 735.3(100), 705.3(8), 614.3(2), 613.2(27), 595.3(2), 585.3(15), 567.2(9), 539.1(2), 521.3(2), 417.2(34), 295.2(2), 251.4(2) | 761 | 32.16 | 821.5 | MS2[821.5@35]: 803.2(12), 759.4(8), 645.3(2), 351.1(100), 259.0(2) |
| 762 | 32.17 | 329.4 | | MS2[329.4@35]: 329.3(100), 311.3(10), 293.4(3) | 773 | 32.11 | 793.6 | MS2[793.6@35]: 775.3(4), 749.5(3), 731.4(5), 707.3(3), 661.5(4), 644.5(3), 643.4(20), 625.3(4), 599.6(2), 553.5(18), 485.5(100), 441.5(9), 423.6(3), 355.5(6) |
| 763 | 32.33 | 837.5 | | MS2[837.6@35]: 819.5(9), 775.4(15), 713.6(2), 661.4(7), 643.2(3), 485.5(2), 351.1(100), 289.1(3) | 774 | 32.82 | 821.6 | MS2[821.6@35]: 803.4(13), 777.8(5), 759.5(11), 715.7(2), 697.6(2), 687.4(2), 645.4(8), 627.4(5), 351.1(100), 289.1(3) |
| 764 | 32.51 | 839.6 | | MS2[839.6@35]: 821.4(12), 795.5(3), 779.4(2), 777.3(3), 733.2(2), 663.4(8), 352.3(2), 351.1(100), 333.1(3) | 775 | 32.95 | 329.3 | MS2[329.4@35]: 329.3(100), 328.6(2), 311.4(14), 310.5(2), 293.4(2), 201.3(4), 171.3(4) |
| 765 | 32.46 | 1015.1 | | MS2[1015.1@35]: 391.3(100) | 776 | 33.02 | 637.3 | MS2[637.4@35]: 637.2(5), 619.2(2), 593.3(7), 578.1(2), 543.1(4), 517.2(6), 497.2(100), 496.5(2), 482.2(8), 475.1(10), 459.9(2), 449.2(44), 433.2(4), 429.2(3), 377.2(82), 362.3(7), 335.1(2), 309.1(7) |
| 766 | 32.16 | 999.1 | | MS2[999.7@35]: 999.4(100) | 777 | 33.28 | 517.5 | MS2[517.5@35]: 517.4(9), 499.3(3), 473.5(4), 456.4(8), 455.4(100), 454.7(2), 439.6(6) |
| 767 | 32.31 | 991.4 | | ---- | 778 | 33.55 | 967.5 | MS2[967.5@35]: 950.6(2), 949.5(12), 905.5(2), 881.1(2), 645.4(6), 498.2(7), 497.1(100), 435.2(5), 407.1(5), 339.1(19), 321.1(5) |
| 768 | 32.40 | 981.4 | | MS2[982@35]: 1001(2), 965(2), 963(4), 660(3), 659(4), 498(2), 497(100), 497(9), 435(6), 339(19), 321(2) | 779 | 33.13 | 807.5 | MS2[807.5@35]: 747.4(2), 717.4(2), 687.4(10), 645.4(100), 644.8(2), 627.5(8) |
| 769 | 32.47 | 957.4 | | ---- | 780 | 33.57 | 969.7 | MS2[969.7@35]: 951.5(6), 907.5(5), 894.6(2), 647.5(2), 497.2(100), 479.1(2), 435.2(7), 407.2(4), 339.2(15), 321.2(3) |
| 770 | 32.57 | 235.2 | | MS2[235.2@35]: 235.2(100), 234.6(5), 220.2(74) | 781 | 33.58 | 329.4 | MS2[329.4@35]: 329.3(100), 328.7(4), 311.3(10), 293.4(2), 201.4(5), 171.3(3) |
| 771 | 32.62 | 985.5 | | MS2[985.5@35]: 967.5(90), 627.3(14), 497.2(100) | 782 | 32.68 | 1221.7 | MS2[1221.6@35]: 1203.5(79), 1185.4(4), 1177.6(4), 1159.3(16), 1141.5(7), 1087.7(5), 1075.6(4), 1069.6(2), 1045.5(100), 1039.2(5), 1027.6(7), 965.5(35), 951.9(2), 947.6(4), 869.6(3), 789.4(10), 747.5(14), 351.2(14) |
| 772 | 32.68 | 637.4 | | MS2[637.3@35]: 637.4(6), 626.9(2), 622.2(8), 621.4(8), 605.0(3), 593.5(2), 587.3(2), 578.9(2), 565.9(2), 543.1(5), 517.1(61), 502.2(2), 501.2(4), 499.2(6), 497.2(23), 485.4(2), 483.5(3), 469.2(8), 467.2(2), 446.2(2), 429.1(57), 414.2(12), 403.1(2), 395.3(2), 377.3(24), 334.9(2), 325.1(2), 309.1(100), 294.3(12), 293.3(4), 257.3(4) | 783 | 33.58 | 357.5 | MS2[357.4@35]: 357.3(100), 356.3(5), 342.4(15), 329.4(20), 313.5(2), 301.3(71), 291.2(2), 285.4(4), 273.4(3), 261.1(4), 260.5(2), 243.4(5) |
| 784 | 33.69 | 499.6 | | ---- | 786 | 33.70 | 295.2 | MS2[295.2@35]: 295.1(100), 277.2(29), 276.6(2), 251.4(2), 233.3(39), 147.2(6) |
| 785 | 33.64 | 852.7 | | MS2[852.8@35]: 898.1(15), 897.5(100), 853.4(17), 807.6(21), 745.1(42), 583.1(27), 537.4(30) | 787 | 34.00 | 325.2 | MS2[325.2@35]: 325.2(100), 307.3(17), 275.3(43), 263.3(50), 248.4(3), 231.2(4), 177.2(5), 147.2(4) |
| 788 | 34.2 | 700.8 | | MS2[701.0@35]: 701.3(41), 700.5(29), 664.0(12), 550.5(100), 548.9(16), 547.9(24), 532.5(10), 531.9(27), 497.3(35), 488.4(15), 447.5(28) | 795 | 34.34 | 339.3 | MS2[339.3@35]: 339.2(13), 338.3(2), 311.3(100), 295.4(11) |
| 789 | 34.31 | 498.6 | | MS2[498.6@35]: 499.6(22), 498.6(100) | 796 | 34.31 | 839.5 | MS2[839.6@35]: 821.5(12), 795.5(3), 777.5(12), 770.3(3), 753.3(2), 705.6(2), 663.3(4), 351.2(100), 350.6(7), 289.0(3) |
| 790 | 34.07 | 967.5 | | MS2[967.6@35]: 950.4(3), 949.5(9), 905.5(3), 804.5(2), 803.3(2), 645.6(3), 627.9(2), 583.4(3), 537.9(2), 497.2(100), 479.3(6), 435.2(6), 405.3(2), 339.0(13), 321.0(5) | 797 | 34.35 | 821.5 | MS2[821.5@35]: 803.5(7), 777.4(4), 760.5(2), 759.4(22), 645.4(6), 627.4(6), 352.3(3), 351.1(100), 289.1(4) |
| 791 | 34.64 | 807.6 | | MS2[807.5@35]: 789.5(8), 745.5(3), 351.1(100), 332.9(2) | 798 | 34.49 | 475.3 | MS2[475.3@35]: 475.2(56), 460.1(14), 433.2(5), 415.2(100), 400.2(10), 355.3(22), 340.0(3) |
| 792 | 34.08 | 969.5 | | MS2[969.6@35]: 951.5(18), 925.6(4), 907.4(14), 889.3(3), 647.5(3), 629.4(3), 497.2(100), 479.4(2), 478.7(2), 435.1(2), 339.1(9), 321.1(2) | 799 | 34.96 | 369.3 | MS2[369.3@35]: 369.2(77), 354.0(8), 351.2(29), 337.3(74), 327.0(2), 300.2(6), 297.2(3), 285.2(7), 256.1(2), 191.1(100), 177.1(3), 176.2(8), 165.2(2), 151.4(2) |
| 793 | 34.28 | 823.5 | | MS2[823.5@35]: 805.4(4), 804.7(3), 761.5(7), 717.6(2), 647.4(7), 351.1(100) | 800 | 34.54 | 957.3 | ---- |
| 794 | 34.21 | 367.3 | | MS2[367.3@35]: 368.2(14), 367.2(100), 352.2(6), 309.3(2), 298.2(11) | 801 | 34.58 | 837.4 | MS2[837.5@35]: 819.3(4), 775.4(14), 768.6(1), 661.4(11), 643.2(8), 566.5(2), 530.7(5), 523.4(3), 485.4(2), 351.1(100), 333.0(3), 288.9(4) |
| 802 | 34.59 | 955.6 | | MS2[955.7@35]: 956.6(2), 938.4(10), 937.5(33), 912.6(3), 911.4(8), 897.3(2), 894.5(32), 893.6(100), 809.5(8), 747.7(5), 630.6(3), 629.5(28), 612.4(2), 611.2(9), 567.6(2), 540.6(4), 539.6(26), 497.4(5), 483.2(3), 471.6(17) | 811 | 35.53 | 1221.7 | MS2[1221.6@35]: 1203.5(24), 1177.4(2), 1159.5(6), 1087.6(7), 1045.5(100), 965.3(10), 923.7(3), 869.4(6), 747.3(7), 350.8(8) |
| 803 | 34.74 | 359.3 | | MS2[359.3@35]: 360.3(6), 359.2(100), 295.4(2), 279.5(2) | 812 | 35.42 | 823.7 | MS2[823.5@35]: 805.4(10), 761.4(12), 689.3(2), 647.4(9), 351.1(100),  289.2(2) |
| 804 | 34.80 | 807.5 | | MS2[807.5@35]: 789.4(17), 763.2(4), 745.5(3), 631.4(7), 537.7(2), 352.1(2), 351.1(100), 333.1(4) | 813 | 36.15 | 515.4 | MS2[515.4@35]: 515.3(36), 497.3(3), 471.4(22), 453.4(100), 438.3(12), 409.5(2) |
| 805 | 34.97 | 707.1 | | ---- | 814 | 36.17 | 822.9 | MS2[823.1@35]: 823.3(5), 805.1(17), 803.8(11), 795.5(17), 779.4(20), 754.3(23), 744.8(8), 718.7(5), 693.9(6), 686.5(16), 630.8(5), 615.7(5), 565.6(7), 531.2(6), 385.1(5), 362.1(4), 351.2(100), 277.8(5) |
| 806 | 35.20 | 739.2 | | MS2[739.2@35]: 775.6(66), 739.6(100), 713.0(91), 703.3(52), 671.1(71) | 815 | 35.92 | 807.6 | MS2[807.5@35]:807.3(4),763.6(4), 745.5(21), 645.4(2), 628.4(6), 627.4(100), 609.4(5), 583.5(16), 551.4(5), 539.4(2), 537.5(49), 493.5(2), 469.5(7) |
| 807 | 35.38 | 605.3 | | MS2[605.3@35]: 605.2(33), 590.2(100), 573.2(7), 511.2(3), 485.2(60), 483.3(2), 467.4(9), 401.2(56), 386.3(6), 307.1(2) | 816 | 35.93 | 569.1 | MS2[569.1@35]: 551.1(2), 449.0(4), 285.1(100), 283.1(30), 270.1(2), 253.3(3) |
| 808 | 35.43 | 219.3 | | MS2[219.3@35]: 219.2(100), 204.2(4), 175.2(1), 163.3(1) | 817 | 36.01 | 283.1 | MS2[283.1@35]: 283.2(10), 280.0(2), 257.1(100), 240.3(4), 239.3(39) |
| 809 | 35.60 | 353.3 | | MS2[353.3@35]: 354.3(19), 353.3(100), 352.6(6), 308.3(2) | 818 | 36.17 | 415.2 | MS2[415.2@35]: 397.1(23), 309.1(100) |
| 810 | 35.81 | 341.2 | | MS2[341.3@35]: 341.1(6), 323.2(14), 297.2(2), 235.2(4), 231.2(9), 217.2(2), 167.2(100), 165.1(2), 149.3(6), 137.2(4) | 819 | 36.30 | 321.3 | MS2[321.3@35]: 21.3(100), 266.4(1) |
| 820 | 36.46 | 357.3 | | MS2[357.3@35]: 358.2(11), 357.2(100), 293.3(2), 277.3(2) | 824 | 36.91 | 353.3 | MS2[353.3@35]: 354.2(3), 353.2(100), 338.2(19), 321.4(8), 284.1(2), 191.2(7) |
| 821 | 36.47 | 349.1 | | MS2[349.1@35]: 269.3(100) | 826 | 36.62 | 823.5 | MS2[823.5@35]: 805.3(9), 797.9(2), 779.8(3), 761.5(7), 717.4(2), 647.4(10), 629.5(3), 352.2(4), 351.2(100), 333.1(3), 261.1(2) |
| 822 | 36.48 | 849.6 | | MS2[849.6@35]: 850.5(100), 849.4(54), 832.4(31), 831.5(75), 813.2(4), 789.1(6), 787.4(3), 770.7(3), 674.4(13), 673.6(73), 672.9(2), 656.5(3), 655.6(7), 638.5(8), 637.3(4), 614.9(2), 613.3(3), 554.0(3), 552.3(6), 551.3(58), 470.5(7), 469.5(57), 468.8(6), 451.3(2) | 825 | 36.56 | 849.6 | MS2[849.6@35]: 850.5(16), 849.6(12), 832.4(16), 831.3(43), 814.3(4), 803.7(3), 788.6(2), 787.6(5), 769.6(5), 741.0(2), 729.4(3), 674.6(6), 673.6(54), 656.5(3), 655.7(34), 646.3(4), 637.4(18), 613.7(2), 552.4(20), 551.5(77), 496.8(2), 470.6(12), 469.5(100), 467.6(3), 355.5(2) |
| 823 | 36.61 | 700.8 | | MS2[700.8@35]: 701.3(41), 700.3(100), 612.9(23), 610.6(18), 578.8(29), 551.1(26), 426.5(42), 335.4(29) | 827 | 36.88 | 953.5 | MS2[953.6@35]: 936.4(3), 935.5(10), 934.8(2), 908.8(2), 891.3(4), 631.4(4), 498.2(14), 497.2(100), 478.9(2), 435.1(5), 407.1(2), 381.2(3), 339.1(15), 321.1(6) |
|  |  |  | |  |  |  |  |  |
| 828 | 37.02 | 881.0 | | MS2[881.0@35]: 881.2(57), 879.8(31), 861.6(24), 845.4(100), 844.4(38), 764.7(37), 752.7(31), 725.0(41), 716.8(62), 700.8(34), 687.5(31), 666.3(20), 646.5(22), 608.8(34), 409.6(30) | 834 | 38.02 | 339.2 | MS2[339.2@35]: 339.2(5), 245.2(100), 177.1(3), 161.1(12), 117.2(8) |
| 829 | 37.32 | 803.5 | | MS2[803.5@35]: 1316.1(5), 804.5(46), 803.4(85), 786.4(18), 785.3(82), 775.3(6), 757.1(2), 742.4(5), 741.4(25), 727.5(13), 654.3(6), 653.3(44), 629.4(4), 628.4(9), 627.3(50), 612.1(3), 611.4(10), 599.4(3), 539.6(3), 497.4(4), 470.6(2), 469.4(100), 416.9(2), 268.0(2), 227.0(2) | 835 | 38.38 | 339.2 | MS2[339.2@35]: 339.2(73), 338.3(11), 321.2(25), 295.2(4), 293.3(12), 245.3(4), 233.3(3), 229.2(46), 179.2(2), 177.3(8), 167.2(100), 166.3(6), 165.1(4), 161.2(2), 149.3(5), 148.2(3), 137.3(3) |
| 830 | 37.05 | 353.3 | | MS2[353.3@35]: 354.2(2), 353.2(100), 352.3(4), 338.2(19), 337.6(1), 321.3(1), 308.4(1), 284.2(1), 259.3(1), 165.2(1) | 836 | 38.29 | 353.3 | MS2[353.3@35]: 354.3(2), 353.2(100), 338.3(18), 259.3(1), 218.2(1) |
| 831 | 37.43 | 353.3 | | MS2[353.3@35]: 353.2(100), 352.0(2), 339.1(1), 338.2(2), 323.3(1), 321.4(16), 320.6(1), 309.3(1), 284.2(2), 282.2(1), 243.3(8), 229.2(4), 227.3(5), 217.3(1), 177.2(4), 175.3(2), 165.3(1), 125.2(3) | 837 | 38.30 | 517.5 | MS2[517.5@35]: 518.5(2), 517.4(9), 499.3(3), 473.5(3), 456.4(5), 455.4(100), 439.4(5) |
| 832 | 37.76 | 353.3 | | MS2[353.3@35]: 354.2(16), 353.2(100), 309.4(1), 285.3(3), 284.2(7), 267.3(1) | 838 | 40.48 | 407.3 | ---- |
| 833 | 37.86 | 367.3 | | MS2[367.3@35]: 367.2(100), 352.2(3), 349.3(1), 323.4(1), 309.3(6), 297.2(4), 284.2(1) | 839 | 38.62 | 367.3 | MS2[367.3@35]: 368.2(26), 367.3(100), 352.4(2) |
| 840 | 38.69 | 353.4 | | MS2[353.4@35]: 354.3(21), 353.3(100), 352.5(1), 298.3(1) | 846 | 37.77 | 735.7 | MS2[835.6@35]: 836.5(29), 835.4(26), 818.5(31), 817.5(100), 577.6(3), 537.3(12), 456.3(4), 455.6(64), 453.4(8) |
| 841 | 38.81 | 385.2 | | MS2[385.2@35]: 385.2(1), 384.3(2), 367.1(1), 341.2(100), 340.5(1), 339.2(1), 259.4(1), 193.4(5), 147.2(5) | 847 | 37.96 | 517.5 | MS2[517.5@35]: 517.4(9), 499.4(2), 473.4(3), 456.5(7), 455.4(100), 439.5(4) |
| 842 | 38.98 | 721.3 | | MS2[721.3@35]: 7 721.5(100), 720.5(56), 707.3(3), 703.3(59), 688.6(14), 685.3(7), 683.8(14), 677.5(36), 663.2(9), 662.2(12), 660.8(5), 659.6(23), 632.5(8), 631.4(17), 626.0(10), 595.2(18), 591.0(13), 581.5(17), 573.5(8), 569.5(11), 559.0(17), 554.2(8), 532.2(19), 526.8(16), 503.1(24), 484.9(11), 429.9(9), 365.3(10), 323.2(14), 315.0(12) | 848 | 37.97 | 399.5 | MS2[399.4@35]: 400.4(22), 399.4(100) |
| 843 | 37.11 | 822.9 | | MS2[823.0@35]: 804.6(3), 785.9(2), 778.4(7), 760.3(15), 759.5(2), 742.8(7), 646.4(14), 352.1(39), 351.2(100) | 849 | 37.99 | 323.3 | ---- |
| 844 | 37.69 | 823.6 | | MS2[823.5@35]: 805.3(15), 780.6(2), 761.4(9), 647.4(5), 351.1(100), 333.4(3), 289.2(3) | 850 | 38.09 | 849.6 | MS2[849.6@35]: 831.3(20), 803.4(100), 790.3(2), 789.4(38), 759.6(4), 673.6(2), 645.5(60), 627.5(38), 537.6(12), 469.5(6) |
| 845 | 37.95 | 803.5 | | MS2[803.5@35]: 803.4(49), 785.3(41), 784.6(4), 775.2(5), 741.4(49), 725.8(5), 723.5(2), 695.8(2), 669.6(2), 653.3(59), 627.5(66), 497.6(4), 469.5(100),  252.9(2) | 851 | 38.04 | 835.6 | MS2[835.6@35]: 817.4(2), 673.5(6), 655.4(2), 637.4(14), 565.4(4), 552.5(2), 551.4(33), 507.3(2), 470.4(4), 469.5(100), 425.6(4) |
| 852 | 38.07 | 807.5 | | MS2[807.5@35]: 809.6(2), 808.3(10), 807.4(4), 806.1(2), 789.3(12), 785.1(4), 763.7(5), 747.0(2), 745.3(14), 720.0(2), 718.2(5), 673.6(2), 631.4(13), 523.6(2), 351.1(100), 333.1(4), 289.4(2),  259.2(5) | 857 | 38.34 | 995.7 | MS2[995.7@35]: 995.7(21), 977.8(6), 977.2(20), 959.3(6), 951.5(18), 887.2(14), 831.5(7), 673.2(19), 593.1(12), 537.3(8), 497.1(100), 479.1(5), 339.0(19), 321.3(8) |
| 853 | 37.92 | 969.5 | | ---- | 858 | 38.59 | 997.6 | MS2[997.6@35]: 997.2(13), 979.3(10), 935.5(17), 833.3(20), 789.7(6), 497.2(100) |
| 854 | 38.17 | 894.9 | | MS2[894.9@35]: 877.6(77), 862.8(41), 849.3(100), 825.0(38), 813.2(63), 809.5(99), 803.5(58), 763.2(62), 750.4(35), 709.4(38), 633.5(22), 621.9(28), 423.2(54) | 859 | 38.66 | 821.4 | MS2[821.4@35]: 885.1(3), 839.1(4), 803.2(11), 780.9(3), 777.5(2), 759.1(6), 757.5(2), 684.4(3), 657.9(3), 645.5(4), 627.0(2), 511.0(1), 495.6(2), 351.1(100), 332.9(3), 288.8(2) |
| 855 | 38.21 | 219.3 | | MS2[219.2@35]: 220.2(4), 219.2(100), 202.2(2), 201.2(36), 175.3(6) | 860 | 38.31 | 823.0 | ---- |
| 856 | 38.83 | 337.4 | | MS2[337.4@35]: 338.3(10), 337.3(100),336.6(3), 305.3(4), 243.3(3) | 861 | 38.49 | 849.6 | MS2[849.6@35]: 850.4(23), 849.5(23), 832.5(8), 831.4(49), 813.7(2), 787.5(10), 759.4(3), 743.6(2), 688.5(2), 687.5(4), 674.5(8), 673.5(36), 656.4(2), 655.6(10), 627.4(4), 583.5(4), 566.5(2), 565.7(7), 552.5(14), 551.4(69), 523.3(2),  507.4(6), 495.9(2), 495.2(4), 470.6(9), 469.5(100), 468.8(2), 467.5(7), 425.5(5), 355.3(2) |
|  |  |  | |  |  |  |  |  |
| 862 | 38.68 | 953.6 | | MS2[953.5@35]: 935.3(9), 909.1(2), 498.1(9), 497.1(100), 435.2(4), 339.1(9), 321.1(4) | 866 | 38.91 | 851.5 | MS2[851.6@35]: 852.3(3), 851.2(2), 833.4(30), 832.2(2), 806.4(11), 805.3(100), 804.3(3), 791.4(18), 789.3(2), 761.5(3), 725.6(2), 648.6(4), 647.6(30), 629.5(16), 628.9(2), 597.5(2), 583.3(4), 552.8(2), 471.6(7), 351.1(13) |
| 863 | 38.72 | 851.6 | | MS2[851.5@35]: 852.4(27), 851.6(13), 850.6(6), 834.5(16), 833.3(100), 832.3(9), 808.5(3), 807.4(11), 805.4(5), 804.4(7), 791.5(11), 790.5(8), 789.5(6), 689.5(7), 675.7(10), 657.6(4), 646.3(4), 639.3(3), 567.2(2), 554.6(5), 553.7(11), 471.6(12), 469.7(2), 453.0(2), 361.1(7), 352.4(2) | 867 | 39.15 | 835.5 | MS2[835.5@35]: 836.4(19), 835.5(68), 834.5(4), 826.5(3), 818.3(3), 817.4(100), 816.5(21), 789.5(2), 776.7(2), 775.4(3), 774.3(3), 659.7(3), 641.7(4), 638.7(3), 631.5(2), 623.5(2), 622.5(2), 612.3(2), 537.5(40), 536.6(4), 535.5(3), 481.7(4), 473.1(2), 455.8(4), 360.8(4) |
| 864 | 38.87 | 849.6 | | MS2[849.6@35]: 849.0(2), 831.4(14), 821.3(6), 803.4(100), 802.7(2), 789.4(9), 645.4(22), 627.5(19), 551.4(2), 537.3(4), 470.6(2), 351.2(27) | 868 | 39.66 | 411.6 | MS2[411.6@35]: 411.2(100), 395.5(21), 387.9(23), 382.7(14), 381.1(33), 365.1(39), 354.2(13), 353.4(11), 349.0(8), 336.3(27), 326.9(9), 325.1(10), 317.0(20), 285.1(8) |
| 865 | 38.72 | 803.6 | | MS2[803.6@35]: 803.4(36), 786.4(11), 785.3(43), 742.5(9), 741.4(12), 731.1(3), 727.3(5), 672.7(2), 671.5(3), 654.4(2), 653.5(33), 646.5(3), 628.4(8), 627.3(36), 623.3(2), 619.4(5), 611.4(3), 583.2(3), 539.4(11), 497.1(2), 470.3(8), 469.4(100), 425.5(4), 271.2(2), 227.4(2) | 869 | 38.82 | 805.4 | MS2[805.4@35]: 832.4(1), 805.5(2), 787.4(16), 761.1(1), 745.2(1), 743.6(5), 729.3(1), 671.7(1), 630.4(1), 629.2(4), 611.1(1), 351.2(100), 333.2(2), 289.2(4) |
| 871 | 38.86 | 645.7 | | MS2[645.7@35]: 646.4(4), 645.4(100), 644.7(2), 627.1(2), 601.4(45), 583.3(3), 515.4(20) | 877 | 39.30 | 967.5 | MS2[967.5@35]: 950.3(4), 949.4(24), 907.4(30), 835.5(14), 804.4(12), 803.4(100), 759.5(4), 743.5(6), 691.4(2), 646.5(4), 645.5(67), 627.6(34), 583.5(2), 537.6(3), 497.1(2), 469.4(2), 351.2(2) |
| 872 | 39.07 | 925.3 | | ---- | 878 | 39.33 | 593.4 | MS2[593.4@35]: 593.3(72), 413.2(100), 315.2(25), 277.4(5), 241.3(13), 223.4(3) |
| 873 | 39.06 | 812.9 | | MS2[812.7@35]: 821.4(100), 805.1(2), 804.2(15), 803.3(26), 767.2(2), 761.3(2), 760.5(43), 759.4(74), 725.0(3), 685.0(2), 646.6(2), 645.6(2), 628.5(2), 627.4(10), 577.1(4), 469.3(5), 333.1(3) | 879 | 39.34 | 833.7 | MS2[833.7@35]: 834.5(2), 815.4(17), 788.3(3), 787.4(100), 773.4(16), 629.4(40), 611.5(15), 593.3(2), 567.2(2), 521.5(2), 453.7(2) |
| 874 | 39.26 | 849.6 | | MS2[849.5@35]: 850.5(2), 849.0(5), 832.2(4), 831.5(11), 741.6(3), 673.5(5), 627.6(2), 469.4(6), 352.1(2), 351.2(100) | 880 | 39.46 | 439.3 | MS2[439.3@35]: 439.2(32), 395.2(100), 377.3(4), 337.3(30), 319.4(4), 310.1(5) |
| 875 | 39.20 | 691.0 | | MS2[691.1@35]: 691.0(100), 672.5(48), 645.8(54), 550.2(44), 548.1(46), 452.2(55), 261.0(53) | 881 | 39.50 | 677.3 | MS2[835.6@35]: 817.4(18), 789.4(100), 788.5(3), 775.5(16), 631.5(33), 630.4(2), 613.5(7), 595.5(4) |
| 876 | 38.91 | 967.6 | | MS2[967.6@35]: 967.5(5), 949.4(100), 907.2(4), 836.4(9), 835.4(45), 803.4(19), 792.8(7), 792.1(11), 791.4(36), 773.4(6), 743.4(6), 645.6(7), 627.6(13), 609.5(9), 552.2(5), 551.4(19), 538.6(4), 523.4(5), 469.6(9) | 882 | 39.81 | 480.3 | MS2[480.3@35]: 480.2(13), 479.5(3), 436.2(100), 293.2(39) |
| 883 | 39.14 | 337.3 | | MS2[337.4@35]: 338.2(5), 337.3(100), 305.4(3), 268.2(3), 243.4(4) | 894 | 40.20 | 595.4 | MS2[595.4@35]: 595.3(85), 415.2(100), 315.2(24), 241.2(9) |
| 884 | 39.55 | 335.4 | | MS2[335.4@35]: 336.3(16), 335.3(100) | 895 | 40.46 | 573.1 ^a^ | ---- |
| 885 | 39.86 | 811.4 | | ---- | 896 | 40.88 | 564.4 | MS2[564.3@35]: 504.3(100) |
| 886 | 41.09 | 665.5 | | MS2[665.5@35]: 585.1(2), 385.2(4), 359.2(100), 341.2(2), 323.4(2) | 897 | 41.27 | 457.5 | MS2[457.5@35]: 458.5(7), 457.4(100), 385.5(3) |
| 887 | 39.50 | 835.6 | | ---- | 898 | 41.31 | 515.2 | ----- |
| 888 | 39.53 | 445.0 | | MS2[445.0@35]: 409.5(2), 401.1(7), 400.3(1), 399.0(2), 381.1(100), 380.0(1), 351.0(4), 209.1(2), 173.3(2) | 899 | 41.52 | 468.2 | MS2[468.3@35]: 469.2(17), 468.2(100), 428.0(1), 405.2(1), 378.9(1), 255.3(1), 254.3(8), 234.2(1) |
| 889 | 39.73 | 673.6 | | ---- | 900 | 41.72 | 468.3 | MS2[468.3@35]: 469.1(16), 468.2(100), 448.3(1), 398.1(1), 254.2(9), 234.1(1), 172.9(1) |
| 890 | 39.92 | 351.3 | | MS2[351.3@35]: 352.3(9), 351.2(100), 283.3(16), 265.4(2) | 901 | 39.94 | 339.2 | MS2[339.0@35]: 245.3(100), 177.2(5), 161.0(22), 117.3(11) |
| 891 | 40.04 | 971.5 | | MS2[485.4@35]: 486.6(3), 485.3(30), 484.3(7), 467.2(3), 455.4(100), 441.5(4), 440.4(3), 439.4(2), 393.3(2) | 902 | 40.59 | 421.3 | MS2[421.3@35]: 422.2(7), 421.2(100), 420.5(2), 352.3(1) |
| 892 | 40.12 | 391.4 | | MS2[391.4@35]: 392.2(8), 391.2(100), 390.5(32), 221.1(4), 203.2(10) | 903 | 41.20 | 665.5 ^a^ | MS2[665.5@35]: 585.2(2), 385.1(3), 359.3(100), 341.3(2), 323.4(2), 279.5(2) |
| 893 | 40.34 | 423.3 | | MS2[423.4@35]: 423.2(13), 229.2(100), 193.2(40), 174.2(2) |  |  |  |  |

^a^ detected [M+HCOO]^-^ ion peaks; ^b^ detected [M+H]^+^ ion peaks

**Elucidation of the chemical structures of eight potential active constituents by LC-MS and NMR.** The structures of 8 constituents (**Fig. 2B**) were elucidated by LC-MS, ^1^H and ^13^C NMR and by comparison with the data in literatures.

Compound **833** showed deprotonated molecule at *m/z* 367.1197 [M − H]^–^ (molecular formula C_21_H_20_O_6_). ^1^H NMR showed the diagnostic signals of ABX coupling system at C-3 of coumarin, *i.e.*, δ_H_ 7.14 (d, *J* = 8.3 Hz, 1H, H-6ʹ), 6.37 (dd, J = 8.3, 2.3 Hz, 1H, H-5ʹ), and 6.39 (d, *J* = 2.3 Hz, 1H, H-3ʹ). The signals at δ_H_ 3.36 (d, *J* = 7.0 Hz, 2H, H-1ʺ), 5.22 (t, *J* = 7.0 Hz, 1H, H-2ʺ), 1.79 (s, 3H, CH_3_), and 1.68 (s, 3H, CH_3_) indicated the presence of isopentenyl group. Two aromatic proton signals at δ_H_ 7.96 (s, 1H) and δ_H_ 6.58 (s, 1H) was attributed to H-4 and H-8, respectively. Additionally, one methoxyl signal at δ_H_ 3.83 (s, 3H) was observed. Based on the above data and by comparing with the data in literature [^1^](#_ENREF_1), compound **833** was identified as glycycoumarin.

Compound **836** showed deprotonated molecule [M − H]^–^ at *m/z* 353.1339 (molecular formula C_20_H_18_O_6_) by LC-MS, and exhibited the NMR signals as follows: characteristic isoflavone signal at δ_H_ 8.00 (s, 1H, H-2), *meta*-coupled aromatic proton signals of A ring at δ_H_ 6.22 (d, *J* = 2.1 Hz, 1H, H-6) and 6.33 (d, *J* = 2.2 Hz, 1H, H-8), the isopentenyl group signals at δ_H_ 3.38 (d, *J* = 7.1 Hz, 2H, H-1ʺ), 5.26 (tt, *J* = 7.2, 1.4 Hz, 1H, H-2ʺ), 1.77 (d, *J* = 1.3 Hz, 3H, CH_3_), 1.66 (d, *J* = 1.4 Hz, 3H, CH_3_). In addition, compound **836** shown the *meta*-coupled aromatic proton signals at δ_H_ 6.87 (d, *J* = 2.1 Hz, 1H, H-2ʹ) and 6.72 (d, *J* = 2.0 Hz, 1H, H-6ʹ). Finally, compound **836** was identified as glycyrrhisoflavone by comparing with the data in literature [^2^](#_ENREF_2). Similarly, compounds **840**, **890** and **902** were confirmed as licoisoflavone A, licoisoflavone B, and isoangustone A, respectively, by comparing with their ^1^H and ^13^C NMR spectrum with the related literatures [^3-5^](#_ENREF_3).

Compound **838** showed deprotonated molecule [M − H]^–^ at *m/z* 407. Its ^1^H NMR spectra revealed the typical proton signals of flavanone at δ_H_ 5.30 (dd, *J* = 12.5, 3.1 Hz, 1H, H-2), 3.04 (dd, *J*=17.1, 12.4 Hz, 1H, H-3) and 2.72 (dd, *J* = 17.1, 3.2 Hz, 1H, H-3). In addition, characteristic aromatic proton signals of ABX coupling system at δ_H_ 7.19 (d, *J* = 2.3 Hz, 1H, H-2ʹ), 6.78 (d, *J* = 8.2 Hz, 1H, H-5ʹ) and 7.12 (dd, *J* = 8.2, 2.4 Hz, 1H, H-6ʹ), as well as one aromatic proton at δ_H_ 5.92 (s, 1H, H-6), were observed. The proton signals at δ_H_ 3.19 – 3.16 (m, 2H, H-1ʺ), 3.31(2H, H-1ʺʹ), 5.33 (dp, *J* = 5.8, 1.5 Hz, 1H, H-2ʺ), 5.15 (tt, *J* = 7.3, 1.4 Hz, 1H, H-2ʺʹ), 1.74 (s, 3H), 1.71 (s, 3H), 1.62 (s, 3H), 1.57 (s, 3H) manifested the existence of two isopentenyl groups. Finally, compound **838** was identified as euchrestaflavanone A by comparing with the data in literature [^6^](#_ENREF_6).

Compound **883** appeared the molecular formula of C_21_H_22_O_4_, and proved to be chalcone by the characteristic proton signals at δ_H_ 7.57 (d, *J* = 15.6 Hz, 1H, H-*α*) and 7.98 (d, *J* = 15.6 Hz, 1H, H-*β*). The aromatic proton signals at δ_H_ 7.95 (d, *J* = 8.8 Hz, 2H, H-2ʹ,6ʹ) and 6.88 (d, *J* = 8.8 Hz, 2H, H-3ʹ,5ʹ) indicated the presence of AAʹBBʹ coupling system. Other two aromatic proton signals at δ_H_ 6.46 (s, 1H, H-3) and 7.50 (s, 1H, H-6) were assigned to H-3 and H-6, respectively. Other proton signals were also observed, including one methoxyl group proton signal at δ_H_ 3.88 (s, 3H), double bond proton signals at δ_H_ 6.25 (dd, *J* = 17.9, 10.3 Hz, 1H, H-2ʺ), 4.99 (dd, *J* = 17.9, 1.4 Hz, 1H, H-3ʺ) and 4.99 (dd, *J* = 10.3, 1.4 Hz, 1H, H-3ʺ), and two methyl proton signals at δ_H_ 1.49 (s, 6H). Therefore, compound **883** was identified as licochalcone A [^7^](#_ENREF_7).

The ion at *m/z* 423.1837 [M − H]^–^ ( molecular formula C_25_H_28_O_6_) for compound **893** was provided by HR-ESI-MS. The ^1^H NMR spectrum proton signals at δ_H_ 4.14 (dd, *J* = 8.7, 5.3 Hz, 1H, H-3), 4.60 (dd, *J* = 11.2, 8.8 Hz, 1H, H-2) and 4.51 (dd, *J* = 11.2, 5.2 Hz, 1H, H-2) indicated it was a isoflavanone. Other three aromatic proton signals at δ_H_ 5.93 (s, 1H, H-8), δ_H_ 6.34 (d, *J* = 8.4 Hz, 1H, H-5ʹ), and 6.82 (d, *J* = 8.4 Hz, 1H, H-6ʹ) were observed. The proton signals at δ_H_ 3.20 (d, *J* = 7.3 Hz, 2H, H-1ʺ), 3.35 (d, *J* = 7.5 Hz, 2H, H -1ʺʹ), 5.23 – 5.14 (m, 2H, H-2ʺ,2ʺʹ), 1.77 (s, 3H), 1.74 (s, 3H), 1.67 (s, 3H), and 1.66 (s, 3H) indicated the presence of two isopentenyl groups. These data were in agreement with those of glisoflavanone [^8^](#_ENREF_8).

**Table S3** ^1^H-NMR and ^13^C-NMR spectrum of compounds **833** and **836**.

| Position | Glycycoumarin (**833**) | | | Glycyrrhisoflavone (**836**) | |
| --- | --- | --- | --- | --- | --- |
|  | ^1^H | ^13^C | ^1^H | | ^13^C |
| **2** |  | 163.9 | | 8.00 (s, 1H) | 154.9 |
| **3** |  | 122.1 | |  | 125.3 |
| **4** | 7.96 (s, 1H) | 139.4 | |  | 182.5 |
| **4a** |  | 108.4 | |  | 106.4 |
| **5** |  | 160.1 | |  | 164.0 |
| **6** |  | 115.7 | | 6.22 (d, *J* = 2.1 Hz, 1H) | 100.3 |
| **7** |  | 161.5 | |  | 166.2 |
| **8** | 6.58 (s, 1H) | 99.2 | | 6.33 (d, *J* = 2.2 Hz, 1H) | 94.9 |
| **8a** |  | 155.0 | |  | 159.9 |
| **1ʹ** |  | 120.9 | |  | 123.2 |
| **2ʹ** |  | 157.5 | | 6.72 (d, *J* = 2.0 Hz, 1H) | 122.3 |
| **3ʹ** | 6.39 (d, *J* = 2.3 Hz, 1H) | 104.2 | |  | 144.8 |
| **4ʹ** |  | 157.3 | |  | 146.0 |
| **5ʹ** | 6.37 (dd, J= 8.3, 2.3 Hz, 1H) | 108.1 | |  | 129.9 |
| **6ʹ** | 7.14 (d, *J* = 8.3 Hz, 1H) | 132.9 | | 6.87 (d, *J* = 2.1 Hz, 1H) | 115.0 |
| **1ʺ** | 3.36 (d, *J* = 7.0 Hz, 2H) | 23.8 | | 3.33 (d, *J* = 7.5 Hz, 2H) | 29.5 |
| **2ʺ** | 5.22 (t, *J* = 7.0 Hz, 1H) | 124.0 | | 5.34 (tp, *J* = 7.5, 1.5 Hz, 1H) | 124.1 |
| **3ʺ** |  | 132.4 | |  | 133.1 |
| **4ʺ, 5ʺ** | 1.79 (s, 3H),  1.68 (s, 3H) | 18.1,  26.0 | | 1.73 (s, 6H) | 18.0, 26.1 |
| **-OCH_3_** | 3.83 (s, 3H) | 63.8 | |  |  |

**Table S4** ^1^H-NMR and ^13^C-NMR spectrum of compounds **840** and **890**.

| Position | Licoisoflavone A (**840**) | | | Licoisoflavone B (**890**) | |
| --- | --- | --- | --- | --- | --- |
|  | ^1^H | ^13^C | ^1^H | | ^13^C |
| **2** | 8.04 (s, 1H) | 157.1 | | 8.07 (s, 1H) | 157.2 |
| **3** |  | 124.0 | |  | 123.3 |
| **4** |  | 183.3 | |  | 183.0 |
| **4a** |  | 106.1 | |  | 106.2 |
| **5** |  | 163.8 | |  | 163.8 |
| **6** | 6.26 (d, *J* = 2.1 Hz, 1H) | 100.6 | | 6.26 (d, *J* = 2.0 Hz, 1H) | 100.7 |
| **7** |  | 166.5 | |  | 166.6 |
| **8** | 6.39 (d, *J* = 2.2 Hz, 1H) | 95.0 | | 6.39 (d, *J* = 1.9 Hz, 1H) | 95.1 |
| **8a** |  | 159.9 | |  | 159.9 |
| **1ʹ** |  | 112.2 | |  | 113.2 |
| **2ʹ** |  | 155.8 | |  | 152.9 |
| **3ʹ** |  | 118.6 | |  | 112.5 |
| **4ʹ** |  | 158.4 | |  | 156.1 |
| **5ʹ** | 6.43 (d, *J* = 8.3 Hz, 1H) | 108.9 | | 6.37 (d, *J* = 8.3 Hz,1H) | 110.0 |
| **6ʹ** | 6.85 (d, *J* = 8.3 Hz, 1H) | 129.4 | | 6.95 (d, *J* = 8.3 Hz, 1H) | 131.9 |
| **1ʺ** | 3.38 (d, *J* = 7.1 Hz, 2H) | 23.8 | | 6.74 (d, *J* = 10.0 Hz, 1H) | 118.5 |
| **2ʺ** | 5.26 (tt, *J* = 7.2, 1.4 Hz, 1H) | 124.6 | | 5.65 (d, *J* = 9.9 Hz, 1H) | 130.2 |
| **3ʺ** |  | 131.6 | |  | 77.1 |
| **4ʺ, 5ʺ** | 1.77 (s, 3H)  1.66 (s, 3H) | 26.1,  18.1 | | 1.41 (s, 6H) | 28.2, 28.2 |

**Table S5** ^1^H-NMR and ^13^C-NMR spectrum of compounds **893**, **838** and **902**.

| Position | Glisoflavanone (**893**) | | | Euchrestaflavanone A (**838**) | | Isoangustone A(**902**) | |
| --- | --- | --- | --- | --- | --- | --- | --- |
|  | ^1^H | ^13^C | ^1^H | | ^13^C | ^1^H | ^13^C |
| **2** | 4.60 (dd, *J* = 11.2, 8.8 Hz, 1H)  4.51 (dd, *J* = 11.2, 5.2 Hz, 1H) | 71.7 | | 5.30 (dd, *J* = 12.5, 3.1 Hz, 1H) | 80.5 | 7.98 (s, 1H) | 154.6 |
| **3** | 4.14 (dd, *J* = 8.7, 5.3 Hz, 1H) | 47.7 | | 3.04 (dd, *J*=17.1, 12.4 Hz, 1H)  2.72 (dd, *J* = 17.1, 3.2 Hz, 1H) | 44.1 |  | 123.5 |
| **4** |  | 200.0 | |  | 198.4 |  | 182.5 |
| **4a** |  | 103.5 | |  | 103.6 |  | 106.3 |
| **5** |  | 163.8 | |  | 163.3 |  | 160.7 |
| **6** |  | 109.1 | | 5.92 (s, 1H) | 96.5 |  | 113.2 |
| **7** |  | 166.4 | |  | 166.2 |  | 163.7 |
| **8** | 5.93 (s, 1H) | 96.7 | |  | 109.2 | 6.38 (s, 1H) | 94.0 |
| **8a** |  | 161.8 | |  | 161.7 |  | 157.7 |
| **1ʹ** |  | 116.0 | |  | 129.6 |  | 125.2 |
| **2ʹ** |  | 157.0 | | 7.19 (d, *J* = 2.3 Hz, 1H) | 129.0 | 6.71 (d, *J* = 2.2 Hz, 1H) | 115.0 |
| **3ʹ** |  | 118.1 | |  | 133.4 |  | 144.7 |
| **4ʹ** |  | 155.1 | |  | 156.6 |  | 146.0 |
| **5ʹ** | 6.34 (d, *J* = 8.4 Hz, 1H) | 127.7 | | 6.78 (d, *J* = 8.2 Hz, 1H) | 115.8 |  | 129.9 |
| **6ʹ** | 6.82 (d, *J* = 8.4 Hz, 1H) | 128.0 | | 7.12 (dd, *J* = 8.2, 2.4 Hz, 1H) | 126.1 | 6.87 (d, *J* = 2.1 Hz, 1H) | 122.4 |
| **1ʺ, 1ʺʹ** | 3.35 (d, *J* = 7.5 Hz, 2H)  3.20 (d, *J* = 7.3 Hz, 2H) | 26.1,  26.1 | | 3.19 – 3.16 (m, 2H)  3.31(2H) | 22.6,  29.4 | 5.34 (dddd, *J* = 8.7, 5.8, 2.8, 1.4 Hz, 1H)  5.23 (dddd, *J* = 8.7, 5.8, 2.9, 1.5 Hz, 1H) | 22.4,  29.4 |
| **2ʺ, 2ʺʹ** | 5.23 – 5.14 (m, 2H) | 124.2, 124.2 | | 5.33 (dp, *J* = 5.8, 1.5 Hz, 1H)  5.15 (tt, *J* = 7.3, 1.4 Hz, 1H) | 124.1, 123.9 | 3.35 – 3.32 (m, 4H) | 124.1, 123.5 |
| **3ʺ, 3ʺʹ** |  | 132.3, 131.8 | |  | 131.7,  131.4 |  | 133.0, 132.2 |
| **4ʺ, 4ʺʹ**  **5ʺ, 5ʺʹ** | 1.77 (s, 3H)  1.74 (s, 3H)  1.67 (s, 3H)  1.66 (s, 3H) | 18.1, 18.0  23.7, 23.4 | | 1.74 (s, 3H)  1.71 (s, 3H)  1.62 (s, 3H)  1.57 (s, 3H) | 18.1, 26.1  18.0, 26.1 | 1.78 (s, 3H),  1.73 (s, 6H),  1.66 (s, 3H) | 18.0, 26.0 18.5, 26.0 |

**Table S6** ^1^H-NMR and ^13^C-NMR spectrum of compounds **883**.

| Position | Licochalcone A (**883**) | | |
| --- | --- | --- | --- |
|  | ^1^H | ^13^C |  |
| **C=O** |  | 192.0 | |
| **α** | 7.57 (d, *J* = 15.6 Hz, 1H) | 119.5 | |
| **β** | 7.98 (d, *J* = 15.6 Hz, 1H) | 142.4 | |
| **1** |  | 115.9 | |
| **2** |  | 160.7 | |
| **3** | 6.46 (s, 1H) | 101.0 | |
| **4** |  | 161.6 | |
| **5** |  | 128.6 | |
| **6** | 7.50 (s, 1H) | 130.3 | |
| **1ʹ** |  | 131.7 | |
| **2ʹ** | 7.95 (d, *J* = 8.8 Hz, 1H) | 132.2 | |
| **3ʹ** | 6.88 (d, *J* = 8.8 Hz, 1H) | 116.5 | |
| **4ʹ** |  | 163.6 | |
| **5ʹ** | 6.88 (d, *J* = 8.8 Hz, 1H) | 116.5 | |
| **6ʹ** | 7.95 (d, *J* = 8.8 Hz, 1H) | 132.2 | |
| **1ʺ** |  | 41.2 | |
| **2ʺ** | 6.25 (dd, *J* = 17.9, 10.3 Hz, 1H) | 149.2 | |
| **3ʺ** | 4.99 (dd, *J* = 17.9, 1.4 Hz, 1H)  4.99 (dd, *J* = 10.3, 1.4 Hz, 1H) | 110.8 | |
| **4ʺ, 5ʺ** | 1.49 (s, 6H) | 27.7 | |
| **-OCH_3_** | 3.88 (s, 3H) | 56.2 | |


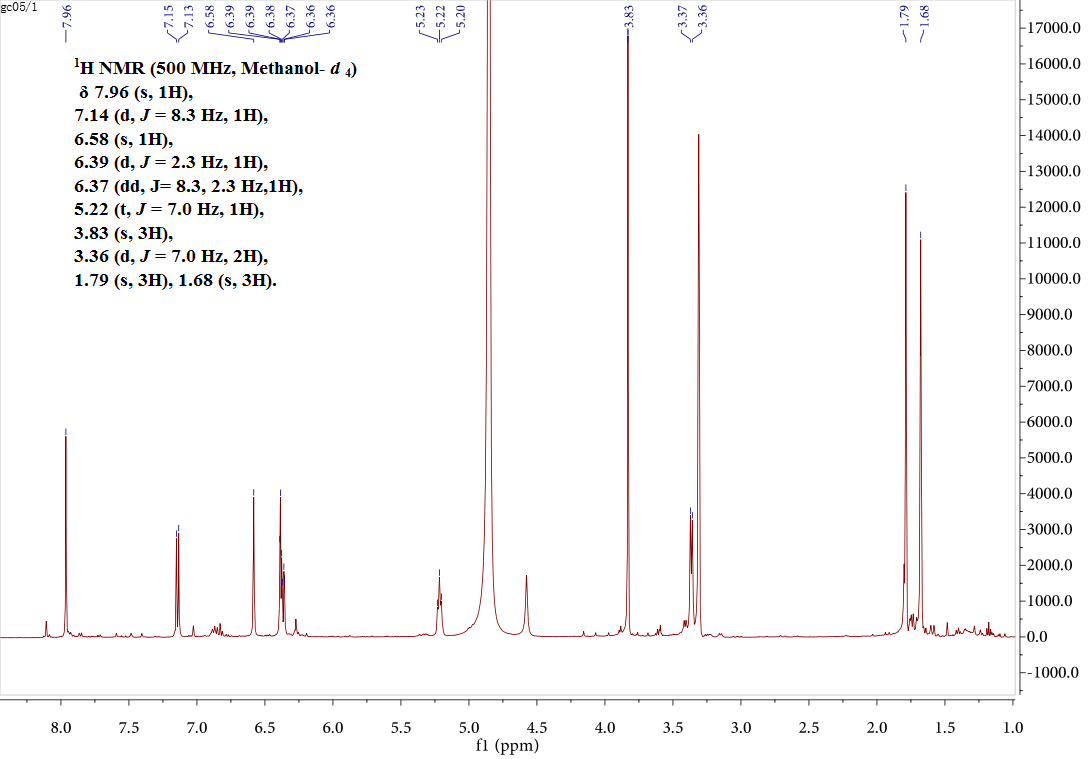


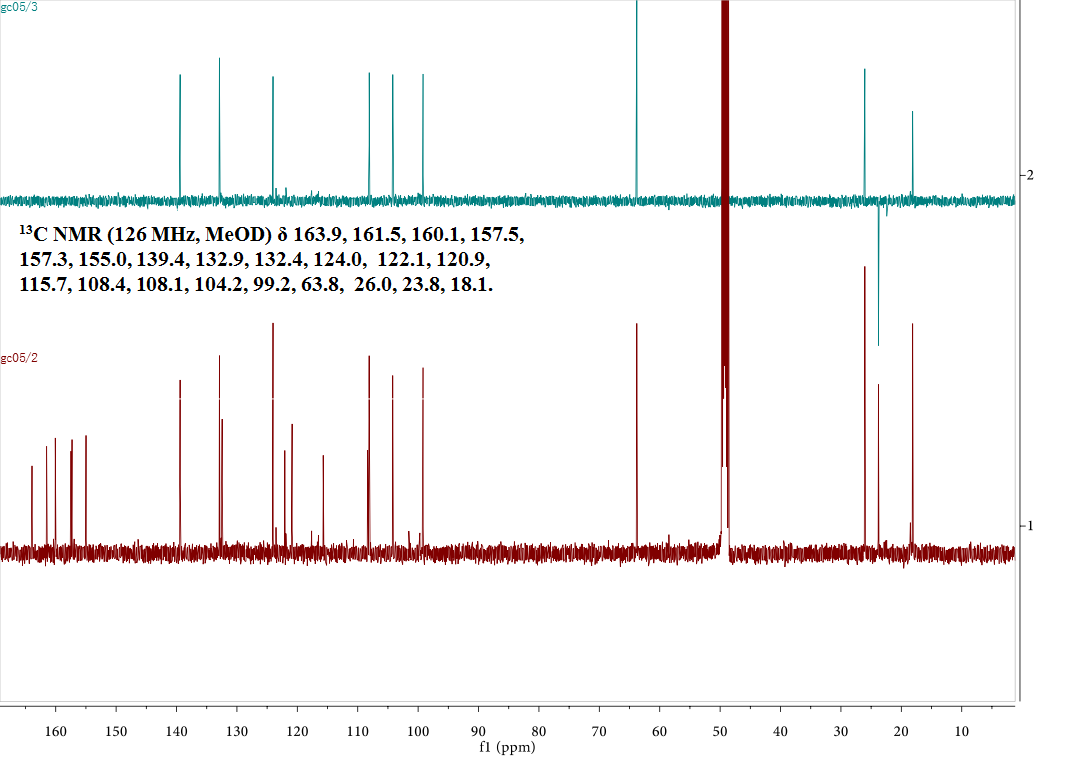


**Figure S1** ^1^H, ^13^C and DEPT 135 NMR spectrum of compound **833**


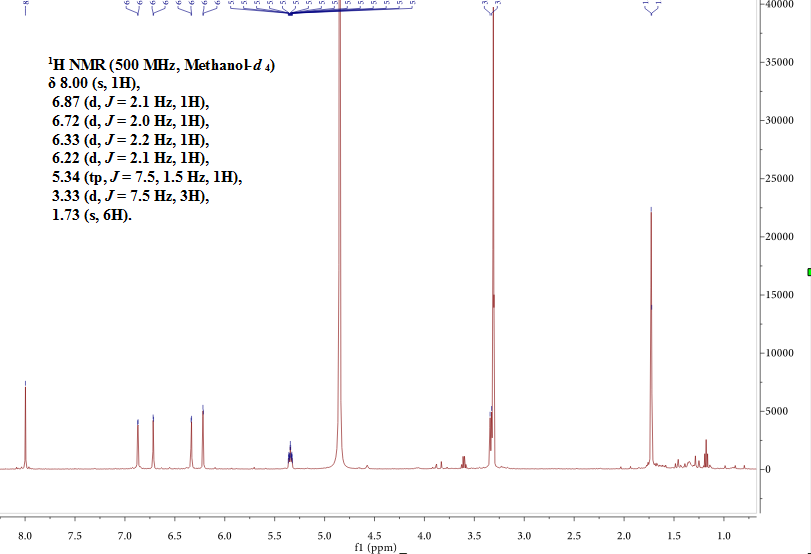


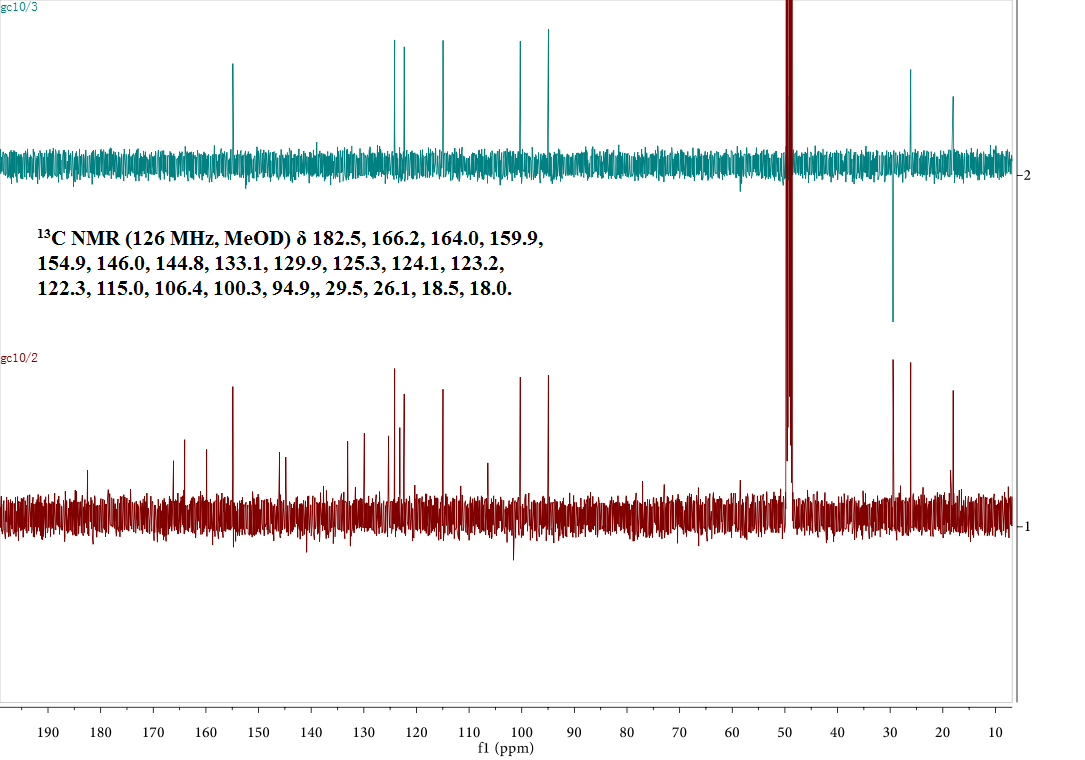


**Figure S2** ^1^H, ^13^C and DEPT 135 NMR spectrum of compound **836**


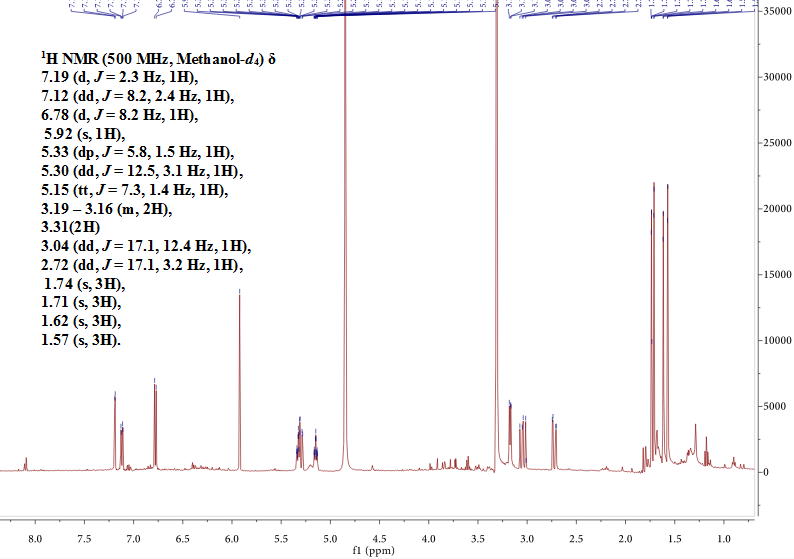


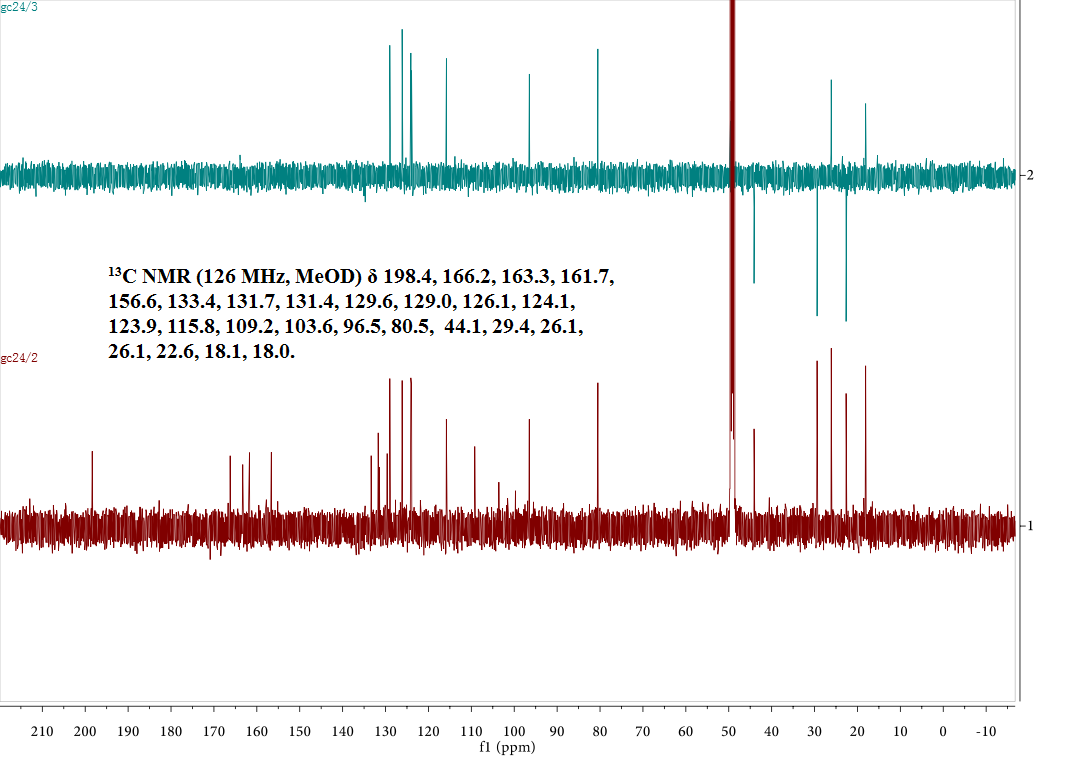


**Figure S3** ^1^H, ^13^C and DEPT 135 NMR spectrum of compound **838**


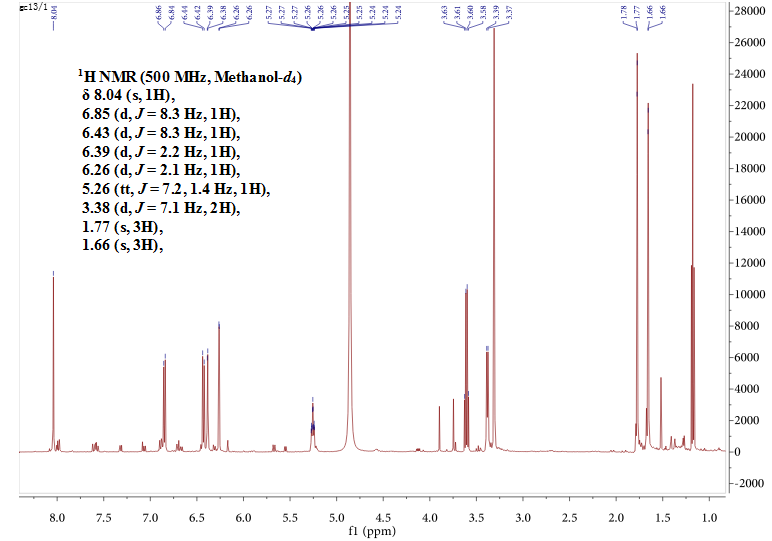


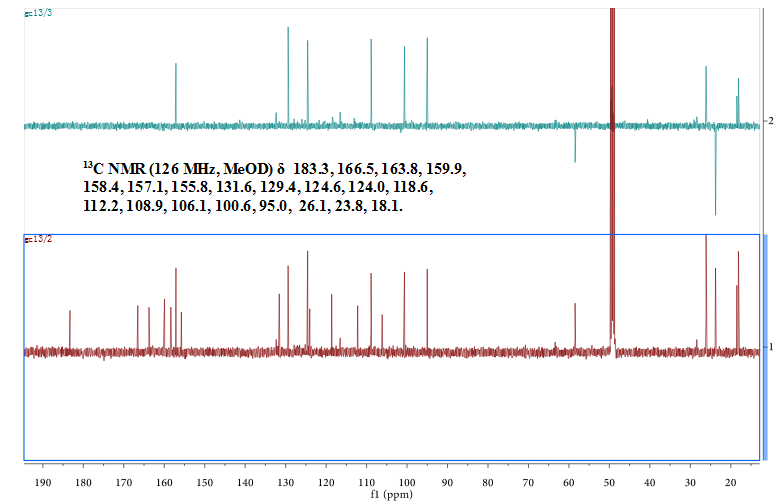


**Figure S4** ^1^H, ^13^C and DEPT 135 NMR spectrum of compound **840**


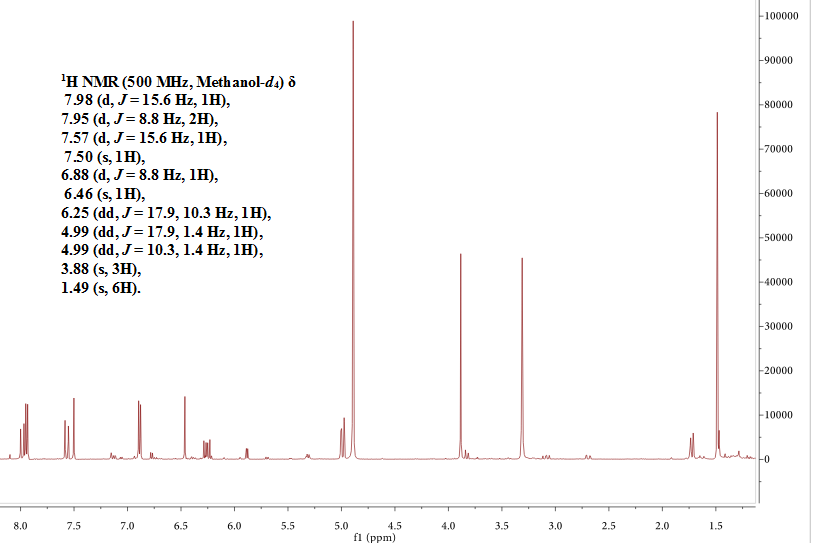


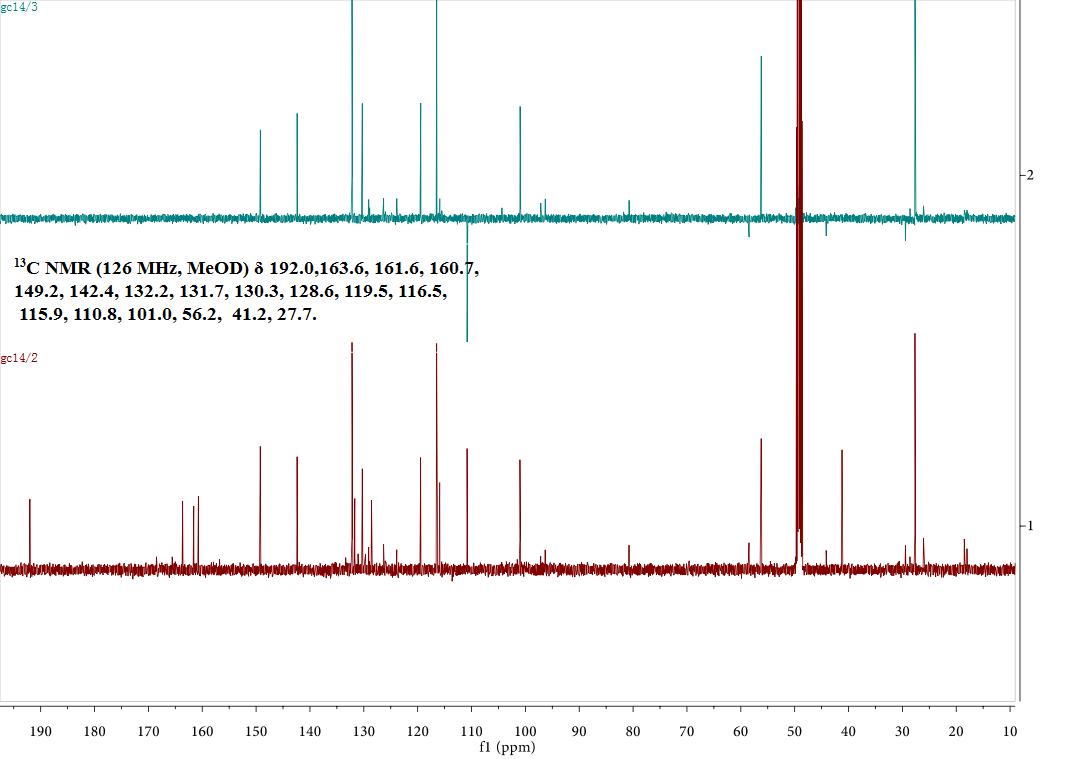


**Figure S5** ^1^H, ^13^C and DEPT 135 NMR spectrum of compound **883**


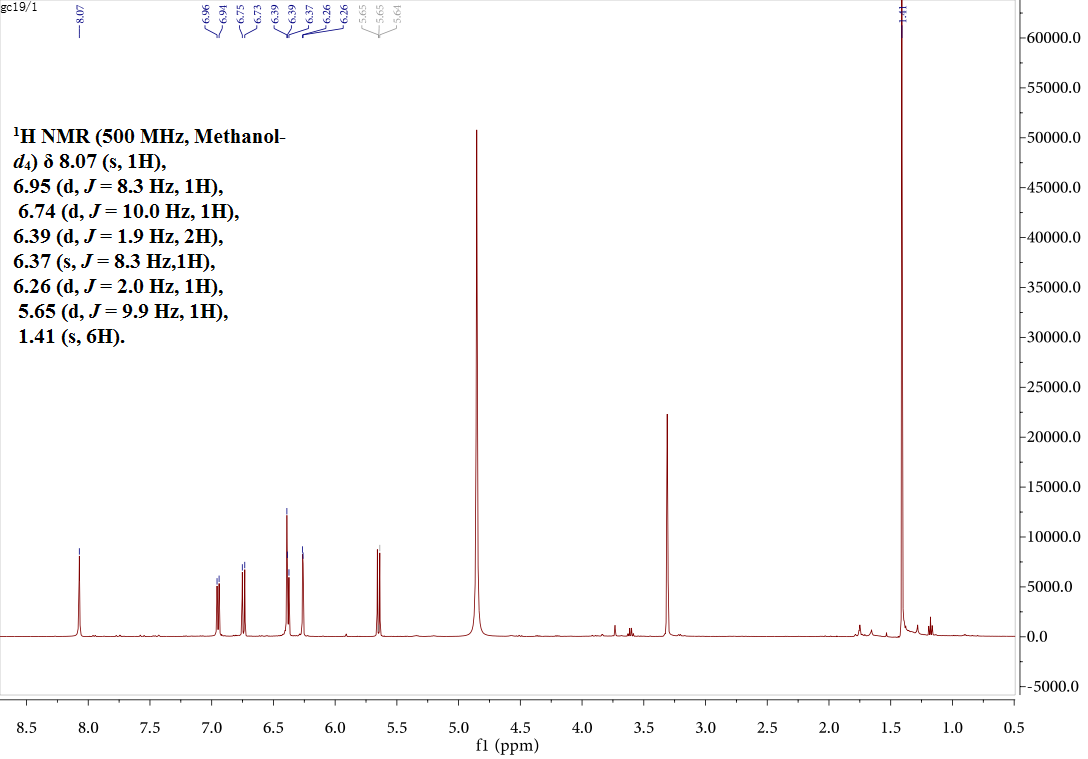


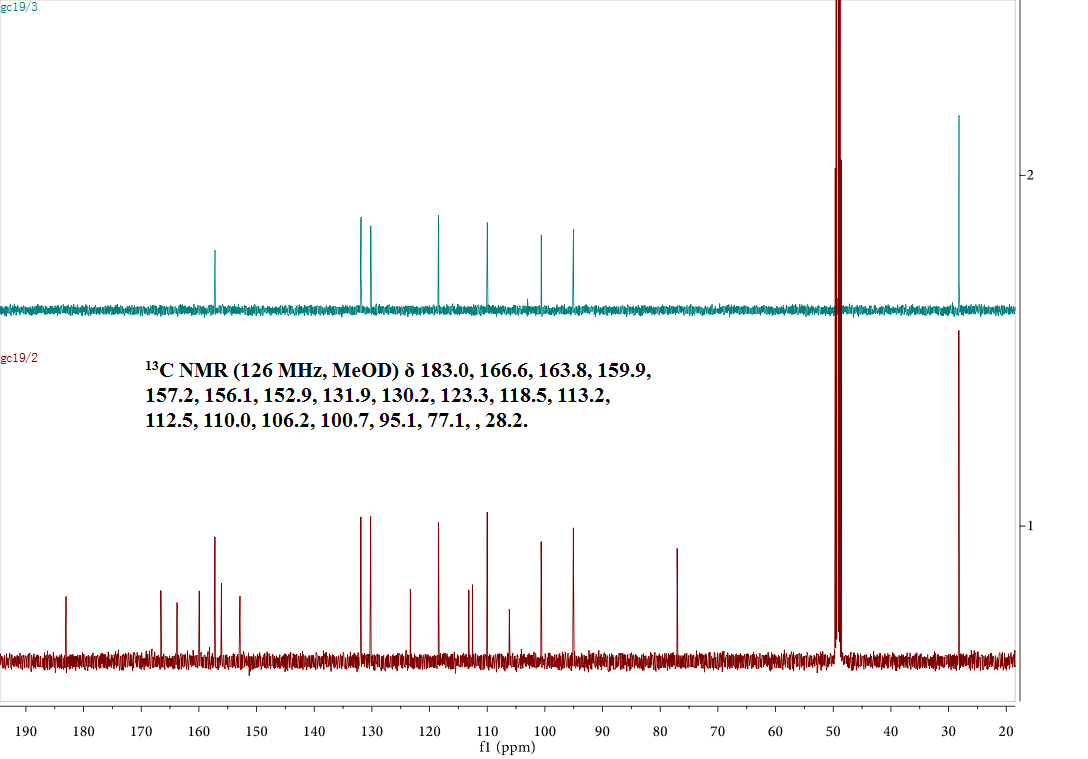


**Figure S6** ^1^H, ^13^C and DEPT 135 NMR spectrum of compound **890**


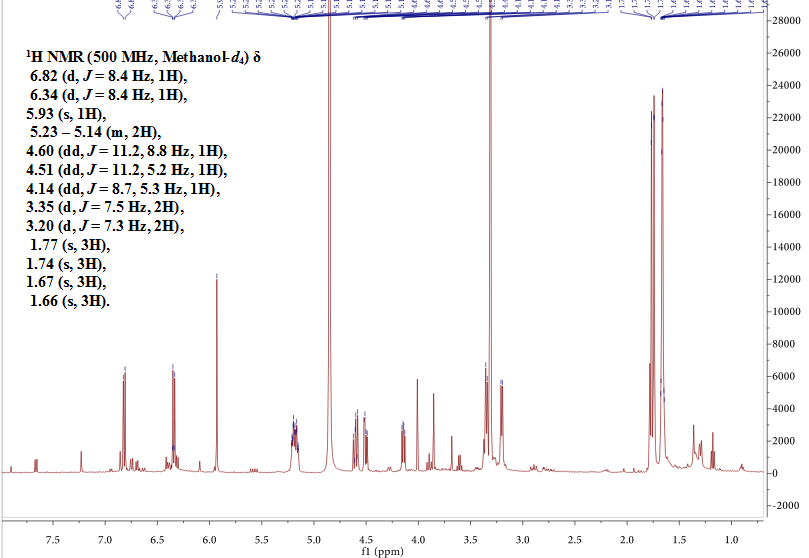


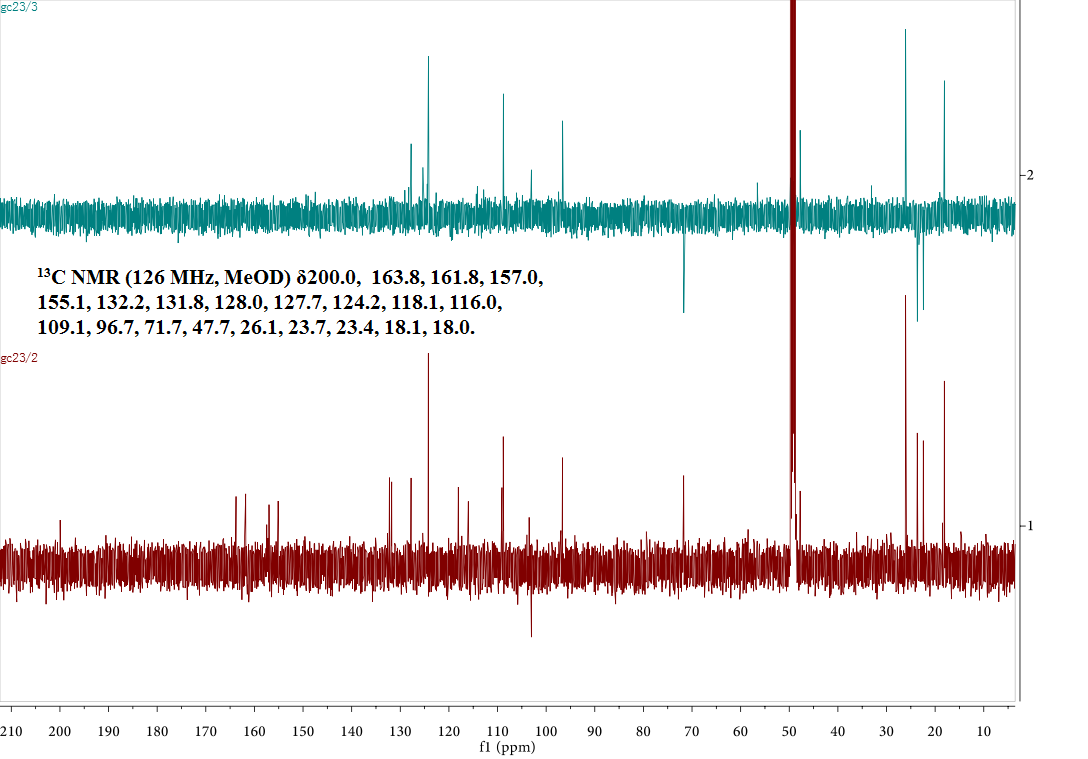


**Figure S7** ^1^H, ^13^C and DEPT 135 NMR spectrum of compound **893**


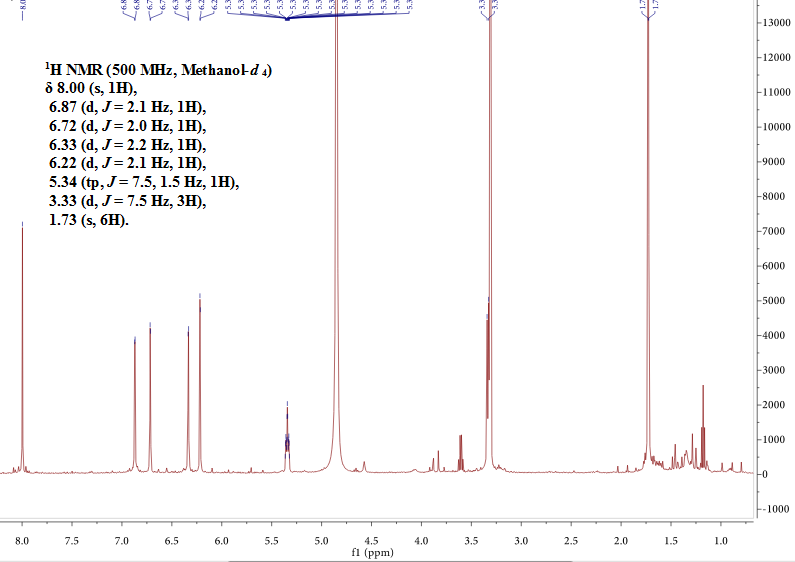


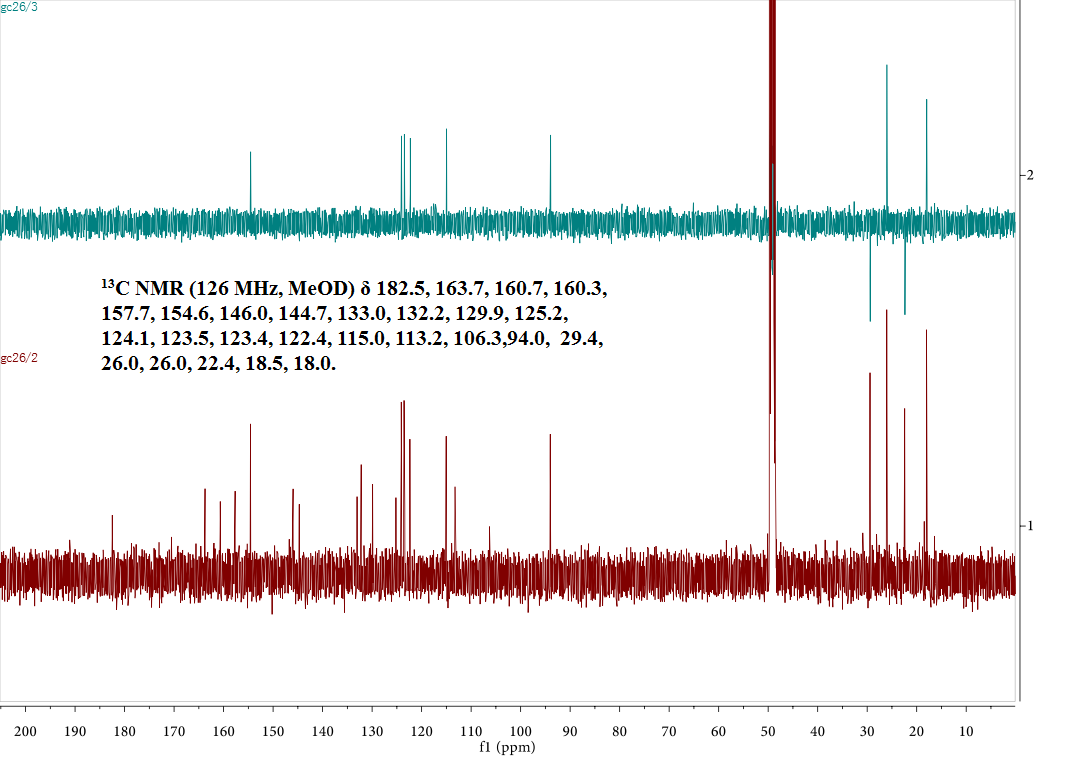


**Figure S8** ^1^H, ^13^C and DEPT 135 NMR spectrum of compound **902**

1. Fu, Y., Chen, J., Li, Y.J., Zheng, Y.F. & Li, P. Antioxidant and anti-inflammatory activities of six flavonoids separated from licorice. *Food Chem.* **141**, 1063-1071 (2013).

2. Salem, M.M. & Werbovetz, K.A. Isoflavonoids and other compounds from Psorothamnus arborescens with antiprotozoal activities. *J. Nat. Prod.* **69**, 43-49 (2006).

3. Zhang, Y.M. et al. Isoflavones from Glycyrrhiza eurycarpa. *Yao Xue Xue Bao* **32**, 301-304 (1997).

4. Wang, Q., Miao, W., Xiang, C., Guo, D. & Ye, M. Chemical constituents in flavonoids from root of Glycyrrhiza uralensis. *Chinese Traditional and Herbal Drugs* **45**, 31-36 (2014).

5. Lee, Y.S. et al. Rapid identification and preparative isolation of antioxidant components in licorice. *J. Sep.Sci.* **33**, 664-671 (2010).

6. Nakahara, K. et al. Prenylated flavanones isolated from flowers of Azadirachta indica (the neem tree) as antimutagenic constituents against heterocyclic amines. *J. Agr. Food Chem.* **51**, 6456-6460 (2003).

7. Simmler, C. et al. Metabolite Profiling and Classification of DNA-Authenticated Licorice Botanicals. *J. Nat. Prod.* **78**, 2007-2022 (2015).

8. Hatano, T. et al. Phenolic constituents of licorice part 9 - Minor flavonoids from licorice. *Phytochemistry* **55**, 959-963 (2000).
